# Quantitative volatile organic compound sensing with liquid crystal core fibers

**DOI:** 10.1016/j.xcrp.2021.100661

**Published:** 2021-12-22

**Authors:** Katrin Schelski, Catherine G. Reyes, Lukas Pschyklenk, Peter-Michael Kaul, Jan P.F. Lagerwall

**Affiliations:** 1 Department of Physics and Materials Science, University of Luxembourg, 162a Avenue de la Faiencerie, 1511 Luxembourg, Luxembourg; 2 Institute of Safety and Security Research, University of Applied Sciences Bonn-Rhein-Sieg, von-Liebig-Straße 20, 53359 Rheinbach, Germany; 3 Lawrence Livermore National Laboratory, Livermore, CA, USA

**Keywords:** volatile organic compound (VOC) sensing, gas sensor, liquid crystal, electrospinning, core-sheath fibers, non-woven fiber mats, wearable technology

## Abstract

Polymer fibers with liquid crystals (LCs) in the core have potential as autonomous sensors of airborne volatile organic compounds (VOCs), with a high surface-to-volume ratio enabling fast and sensitive response and an attractive non-woven textile form factor. We demonstrate their ability to continuously and quantitatively measure the concentration of toluene, cyclohexane, and isopropanol as representative VOCs, via the impact of each VOC on the LC birefringence. The response is fully reversible and repeatable over several cycles, the response time can be as low as seconds, and high sensitivity is achieved when the operating temperature is near the LC-isotropic transition temperature. We propose that a broad operating temperature range can be realized by combining fibers with different LC mixtures, yielding autonomous VOC sensors suitable for integration in apparel or in furniture that can compete with existing consumer-grade electronic VOC sensors in terms of sensitivity and response speed.

## Highlights

• Fully quantitative demonstration of VOC sensing with liquid crystal (LC) core fibers
• Threshold VOC concentration depends sensitively on the clearing point of the LC
• Response time is reduced from minutes to seconds by pre-exposure to VOC
• Strategy for realizing competitive autonomous VOC sensors based on LC core fibers

## Graphical abstract

**Figure:**
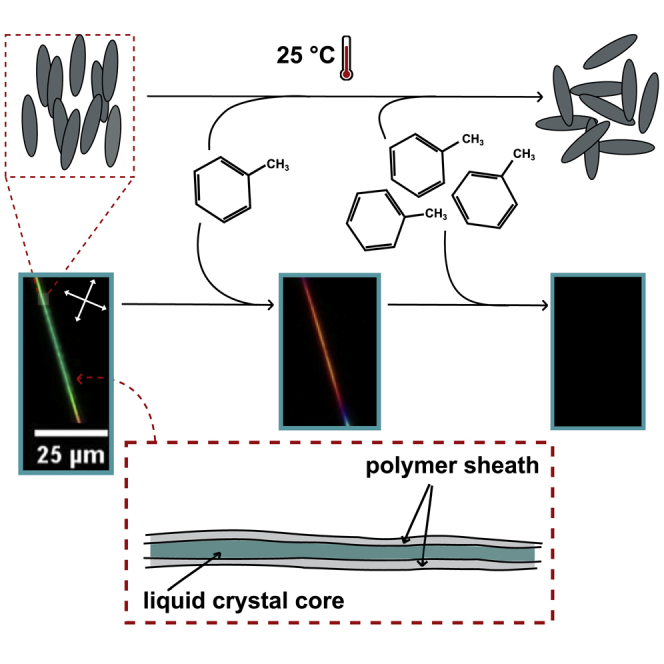

## Teaser

Schelski et al. demonstrate that liquid crystal core-functionalized polymer fibers produced by electrospinning can sense volatile organic compounds (VOCs) rapidly, quantitatively, and fully autonomously, with highly repeatable performance. Their textile form factor is ideal for integrating in clothing or furniture, enabling new opportunities in ubiquitous VOC monitoring, at home and in public spaces.

## Introduction

The air that we breathe is a complex mixture of many chemicals, with the fraction composed by volatile organic compounds (VOCs) being of particular interest. The presence of certain VOCs in exhaled air can be a sign of severe health conditions,^1^ and there is thus a strong interest in quantitative monitoring of VOCs in air for medical diagnostics.^2^ Airborne VOCs can indicate imminent danger, being released by bacterial growth in food^3^^,^^4^ or by explosive devices;^5^ hence, their detection can alert people of spoiled meals or of attempted terrorist attacks. Moreover, they can have serious health effects upon inhalation, causing disease and/or allergenic reactions. The increasing awareness of these negative health effects has led to a surge in public interest in detecting VOCs, and there are now several electronic products for home usage on the market. Unfortunately, they rarely distinguish between different VOCs, as this is highly challenging for consumer-grade electronics-based sensors.^6^, ^7^, ^8^ Better selectivity may be provided from specific chemical interactions between a target VOC and a dye or other responsive material, which reports the VOC presence through a change in appearance.^9^, ^10^, ^11^ An interesting class of such optically VOC-responsive materials is given by liquid crystals (LCs),^12^^,^^13^ which despite their liquid nature exhibit long-range orientational order along a direction called the “director.”^14^ This gives them properties normally seen only in crystalline solids, such as birefringence and, in case of chiral LCs, structural color.^15^ LCs have been shown to signal exposure to toluene,^16^, ^17^, ^18^, ^19^, ^20^ acetone,^19^^,^^21^ NO_2_,^22^ CO_2_,^23^^,^^24^, O_2_,^24^ amines,^25^ cyclohexane and acetic acid,^26^ chloroform and ethanol,^27^^,^^28^ isopropanol,^29^ tetrahydrofuran, methanol, tetrachloroethylene,^27^ pyridine, hexane, and benzene^21^ and to VOCs mimicking the nerve gas sarin.^30^, ^31^, ^32^, ^33^ Apart from making selectivity to specific VOCs easier than for typical consumer-grade electronic sensors, the non-electronic response of LCs, being fundamentally a VOC-induced change of optical characteristics driven by thermal energy alone, renders them ideal for autonomous sensors that require no power source.

A problem for LC-based sensors is, however, to find a convenient form factor, as the liquid nature renders an unprotected LC sensitive to mechanical impact, and an open flat LC sample has only one surface exposed to the VOC, giving a low surface-to-volume ratio, which increases the response threshold concentration and response time. An interesting approach to address these challenges is to encapsulate the LC into the core of thin fibers with a polymeric sheath produced by electrospinning.^14^^,^^16^ This ensures a high surface-to-volume ratio with access to VOC on all sides, as well as a convenient containment, mechanically protecting the LC while still allowing VOCs to permeate through the thin polymer sheath, and giving access to the optical properties of the LC. A further attractive aspect is the textile form factor, ideal for incorporation in apparel as wearable devices or within home/office furniture.

**Figure 1:**
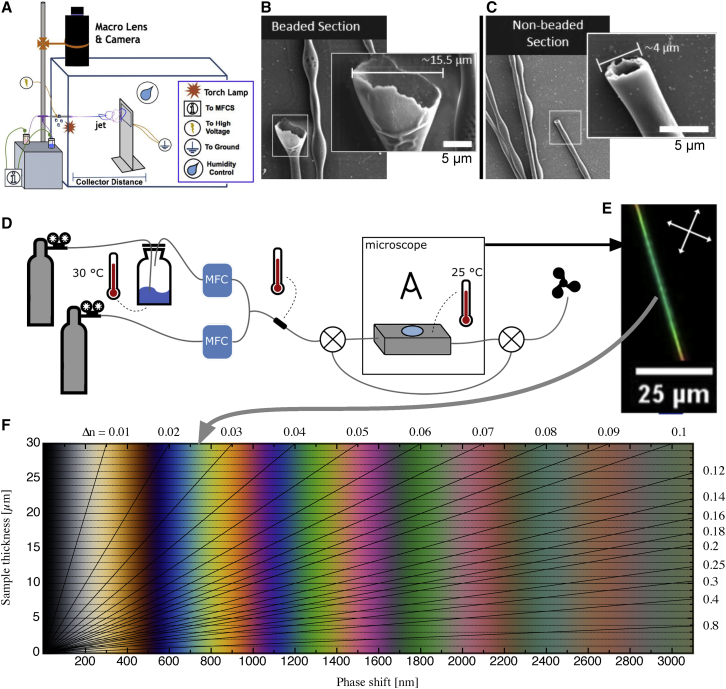
Concept graphic for quantitative VOC sensing using LC-functionalized electrospun fibers The key components of our horizontal electrospinning setup are shown in (A); MFCS is a pneumatic flow control unit. Two examples of fractured fibers imaged by scanning electron microscopy (SEM) show cross sections of beads (B) and non-beaded fiber segments (C), respectively. The hollow interior was filled with LC prior to evacuation for SEM imaging. During VOC sensing tests (D), the fibers are kept in a gas-tight cell with glass windows, placed on a polarizing optical microscope (POM), and connected to a mixed flow of inert carrier gas and VOC at controlled concentration. The LC birefringence (Δ*n*) gives the fiber a characteristic color in POM (E), the identification of which in the Michel-Lévy diagram (F) allows us to establish Δ*n*. Since Δ*n* reflects the LC orientational order *S*, this allows us to quantify the VOC impact on *S*, which is the basis for measuring the VOC concentration.

In brief (Figure 1A), electrospinning^34^ relies on the charging of a drop of polymer solution or melt (we use the former) by a strong electric field until the attraction to a grounded or oppositely charged collector is so strong that it overcomes the surface tension. A thin jet ejects and moves rapidly, under strong simultaneous stretching, toward the collector, eventually forming the dry fiber if the polymer molar mass is high enough. If not, the Rayleigh instability breaks the jet into droplets (electrospray) before solidification. The LC is often incorporated as a distinct core already at the spinneret level^35^ using a coaxial spinning geometry,^36^ but it may also be dissolved in the polymer solution, relying on phase separation during solvent evaporation for the appearance of the coaxial geometry.^37^ We refer readers interested in the details pertaining to LC-functionalized fiber spinning to the now quite rich body of prior research (see, e.g.,^19^^,^^35^^,^^37^, ^38^, ^39^, ^40^, ^41^, ^42^, ^43^, ^44^, ^45^, ^46^, ^47^).

Much of the work so far focused on fabricating the fibers reproducibly, as the many steps of the procedure entail challenges from a general materials science and physical chemistry point of view. These range from the complex phase behavior when the LC comes into contact,^48^ or is co-dissolved,^19^^,^^39^ with polymer solutions, followed by the strongly non-equilibrium situation as solvent evaporates under the sometimes counterintuitive impact of humidity in the air, affecting the process from the emergence of the Taylor cone at the spinneret^46^ to the wetting of the collected fibers on the substrates.^43^ Even the effects on the LC self-assembly by the strong cylindrical confinement are far from trivial.^35^^,^^38^^,^^40^, ^41^, ^42^ This focus has left the demonstrations of the gas sensing functionality largely on a qualitative level, with only two reports showing semiquantitative (arbitrary units) data.^19^^,^^20^

Moreover, almost all experiments have been done with the same single-component LC, 4-cyano-4-pentylbiphenyl (5CB),^16^^,^^18^, ^19^, ^20^ exhibiting a nematic LC phase at room temperature, with a nematic-isotropic transition (clearing) at *T*_*NI*_ = 35.6°C. Since these studies relied on triggering the nematic-isotropic transition by the VOC exposure, the role of *T*_*NI*_ clearly plays an important role, yet its impact has not been investigated. One study used cholesteric LC mixtures with higher clearing points,^23^ but in that case the response was in terms of a change in the pitch of the cholesteric helix, without triggering clearing. In this study, we spin coaxial fibers with core consisting of 5CB or the 5CB-based nematic mixture E7 (*T*_*NI*_ ≈ 58°C), and we follow a path toward quantitative analysis that we initiated earlier.^20^ We carefully and continuously monitor the magnitude of birefringence (Δ*n*) of the fiber-encapsulated LC in response to varying VOC concentration. While we confirm the response to toluene, isopropanol, as well as cyclohexane, we focus primarily on toluene, correlating Δ*n* to the concentration of toluene vapor (*c*_*tol*_) as measured by a calibrated laboratory-grade electronic toluene sensor.

## Results

### Polarizing microscopy on unexposed electrospun fibers

**Figure 2:**
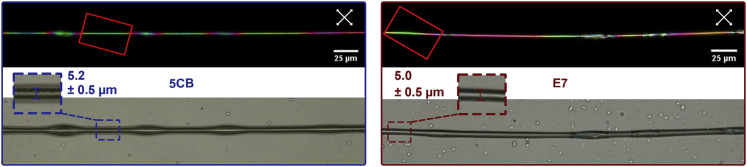
Pristine electrospun PVP sheath fibers filled with 5CB and E7, respectively Between crossed polarizers (upper row; white double arrows indicate polarizer orientations), interference colors due to LC birefringence can be observed. The red rectangles mark the regions monitored during toluene exposure in Figures 3 and 4. The lower row shows the same fiber sections without analyzer, allowing the distinction between beaded and non-beaded parts. These images are also used to measure the diameters (see insets; accuracy limited to ±0.5 μm by resolution of POM). The outermost dark-gray region surrounding the 5CB-filled fibers is not the fiber edge but a thin coating on the substrate. Most likely, this coating formed as condensation water spread on the glass substrate,^43^ bringing some dissolved PVP with it. Scale bars, 25 μm (valid for images with and without analyzer).

Before exposing our fibers to VOCs, we investigate them in the pristine state in polarizing optical microscopy (POM). The morphology of LC-filled fibers often varies somewhat, e.g., with “beads” of locally increased diameter (Figure 1B) appearing with semi-regular intervals along a fiber that is otherwise cylindrical (Figure 1C). We see this with E7 as well as with 5CB core (see Figure 2 for representative examples). While non-beaded sections have an average diameter of ∼4 μm, beads vary in size, from the most common marginal expansion to more extreme cases where the local bead diameter surpasses 10 μm.

When viewed at 25°C between crossed polarizers, the LC-filled fibers show intense interference colors reflecting the relatively high Δ*n* of the encapsulated LC. This is in strong contrast to homogeneous fibers of pure polyvinylpyrrolidone (PVP), which appear dark in POM, since the type of PVP used in our study does not crystallize (see Figure S1). Occasionally, weak birefringence of pure PVP fibers can be observed and arises due to the strong stretching of the jet in-flight, aligning the polymer chains to some extent along the final dry fiber.^49^ However, this alignment may not occur in the LC-filled fibers: the strain experienced by the drying polymer solution is very different in the coaxial geometry, around a non-volatile core, and the tensile strain along the jet is shared between core and sheath. With coaxial fibers we may thus expect low Δ*n* for the sheath, which, together with the fact that the imaged PVP is much thinner than in pure PVP fibers (because the same volume of PVP is distributed around the LC core), leads to negligible impact from the sheath in POM. Indeed, as will be clear from later figures, a 5CB- or E7-filled fiber imaged in POM appears black if the core goes isotropic. Any birefringence remaining in the polymer sheath can thus safely be ignored in comparison to that of the LC core. This is important for the validity of the quantitative measurements described below.

The director, and thus the optic axis, of nematic LCs incorporated within electrospun fibers generally orients along the fiber. This can be verified by investigating the fibers in POM, orienting them along and perpendicular, respectively, to the optic axis of an inserted first-order λ plate (Note S1). Upon rotating the fibers in the POM without the λ plate we can also confirm to what extent the director orientation is uniform within the fibers: in Figures S3E and S3F we find that the straight segments between beads appear perfectly dark every time the fiber is oriented along one of the crossed polarizers, while the beads never go dark, regardless of fiber orientation. They only shift their interference color as the sample is rotated. This indicates that the director orientation is uniform in the segments between beads, while in beads, particularly large ones, we have a (partially) twisted director field, where the optic axis at the top of the fiber is not parallel to that at the bottom, hence precluding extinction. Its optics is significantly more challenging to analyze quantitatively than that of a uniform director field. For this reason, the gas sensing measurements presented below do not show data from the beaded sections of the fibers, as they cannot be directly compared with the classic Michel-Lévy chart describing the interference color of birefringent samples with uniform optic axis directions.

**Figure 3:**
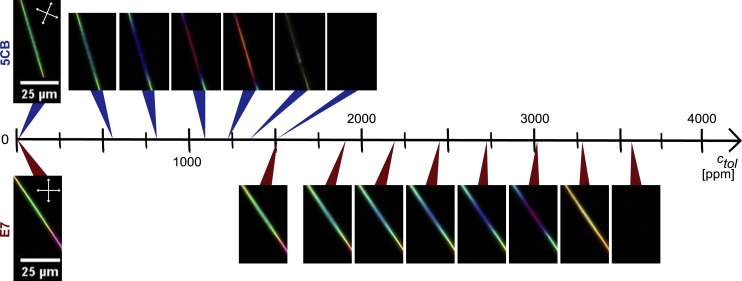
Optical response to toluene exposure Representative non-beaded segments of two electrospun fibers (within red rectangles in Figure 2) filled with 5CB (top) and E7 (bottom), respectively, responding to their first exposure to toluene vapor, at successively increasing concentration *c*_*tol*_ (without purging with pure nitrogen in between), at a temperature of 25°C. The diameters, including the PVP sheath, are 5.2 ± 0.5 μm (5CB core) and 5.0 ± 0.5 μm (E7 core). Each image shows the appearance between crossed polarizers (white double-headed arrows) at the end of a 1-min exposure at constant *c*_*tol*_. The white scale bar applies to all images, extracted from video recordings of the experiments (Video S1).

**Figure 4:**
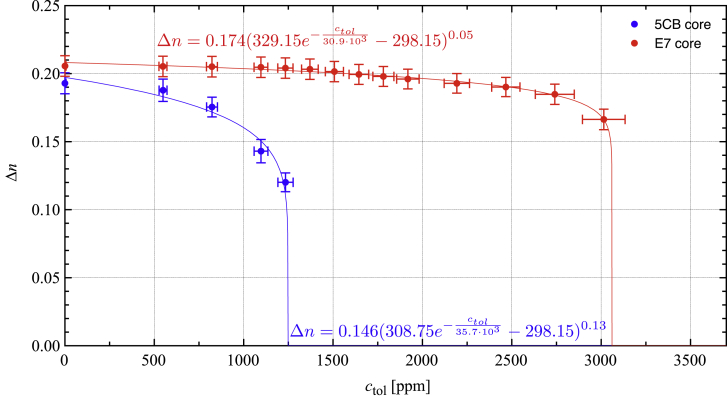
Quantitative analysis of birefringence as a function of toluene exposure Birefringence Δ*n* of segments of 5CB- (blue) and E7-filled (red) fibers (same as in Figures 2 and 3) as a function of toluene concentration *c*_*tol*_ in the atmosphere (first exposure), at a temperature of 25°C. The blue and red curves are best fits of Equation 2 to the experimental data. Each data point is an average of all Δ*n* values during the last 15 s of 1 min of exposure at constant *c*_*tol*_. Each individual Δ*n* value is calculated following Equation 1, assuming *d* to be 70% of the outer fiber diameter. Error bars (see Supplemental experimental procedures) for the concentrations arise from the calibration of the gas sensing setup, described and shown in Supplemental experimental procedures

The untwisted regions between beads are, alternatively, very well suited for such a comparison, forming the basis for our quantitative analysis. We thus limit this analysis to the fiber segments in red rectangles in Figure 2, showing the exact same segments that are analyzed in more detail in Figures 3 and 4. With matching diameters between the two fiber types, those segments are well suited to compare the behavior of the two LCs exposed to toluene vapor (Figure 1D). Furthermore, because Δ*n* at room temperature is only slightly greater in E7 than in 5CB, the fact that the segments appear with similar color confirms that the two selected sample sections have a similar cross section not only in the sample plane but also perpendicular to it, along the viewing direction. As will become clear below, the green color of these segments in their unexposed state corresponds to a second-order green in the Michel-Lévy chart (see gray arrow in Figures 1E and 1F), thus an optical retardation of *r* ≈ 700 nm.

### The response to controlled toluene vapor exposure

In contrast to the previous reports of VOC response of LC-functionalized fibers, which mainly studied entire fiber mats showing varying scattering,^16^ brightness between crossed polarizers,^18^^,^^19^ or color changes,^23^ in this study we use a tailor-made gas exposure cell mounted on a polarizing optical microscope, inspired by Hunter et al.^33^ and proposed in 2018,^20^ to investigate the response at the level of a single LC filled fiber exposed to a specified concentration of VOC (see Figure 1 and Supplemental experimental procedures for details).

When fibers filled with either 5CB or E7 are exposed to toluene vapor, a gradual change in interference color in POM proceeds under increasing *c*_*tol*_ (see Figure 3) The progression of color shift is leftward in the Michel-Lévy chart (see Figure 1F), thus toward reduced Δ*n*. The color changes from the original second-order green to second-order blue, then entering the first order with purple, red, orange, and yellow, before the fiber suddenly turns black, indicating a complete loss of birefringence, i.e., the core has undergone the first-order phase transition from nematic to isotropic. Importantly, this transition is fully reversible, and the LC regains its birefringence once the toluene is removed. Note that the color change is perfectly smooth and continuous throughout the process up to the nematic-isotropic transition, with the images shown being representative snapshots from videos tracking the experiment (see Video S1 for the case of the E7-filled fiber).

Video S1. E7-filled fiber exposed to toluene This video serves as an example of the smooth continuous color transition of a single E7-filled fiber under toluene vapor of stepwise increasing concentrations. A more detailed description can be found in Note S6.

The experiments are successfully repeated about 30 times with fibers produced from several electrospinning processes. In very rare cases, fibers that are apparently malformed are encountered, recognized by the fact that the original texture does not recover after complete clearing and following removal of the analyte (see Note S2). The birefringence returns but with a highly non-uniform texture. Since this phenomenon is rare (studying on the order of 100 fibers, we see this irreversible behavior only in 3), we did not investigate it further. We hypothesize that the sheath may not be fully intact in these fibers, allowing LC flow outside the core during the phase transition, changing the LC configuration irreversibly.

Interestingly, while the sequence of color change is qualitatively identical for 5CB- and E7-filled fibers, we see in Figure 3 that it is shifted to significantly higher *c*_*tol*_ with E7 in the core. For the fiber filled with 5CB (upper row), the first clearly detectable color shift (from the first to the second photo) can be seen at *c*_*tol*_ ≈ 0.6 × 10^3^ ppm, whereas for the one filled with E7 (bottom row), the corresponding threshold is more than 1,000 ppm higher, at *c*_*tol*_ ≈ 1.9 × 10^3^ ppm. In fact, at *c*_*tol*_ = 1.50 × 10^3^ ppm, where the 5CB-filled fiber turns black, indicating the loss of nematic order and Δ*n* = 0, the E7-filled fiber still shows no visible change in POM appearance. For the E7-filled fiber to lose its birefringence, an exposure to *c*_*tol*_ = 3.6 × 10^3^ ppm is required. We also observe that the *c*_*tol*_ range from the first detectable color change to the loss of nematic order is almost twice as large for E7 (*c*_*tol*_ ≈ 1.7 × 10^3^ ppm) compared to 5CB (*c*_*tol*_ ≈ 0.9 × 10^3^ ppm).

To make the analysis of the response quantitative, we turn the detected interference colors into numerical values for Δ*n* at each value of *c*_*tol*_. The full procedure is described in detail in Supplemental experimental procedures. The red, green, and blue values (*R*, *G*, *B*) in the digital image file are first converted into hue values (*H*) of the *HSL* (hue, saturation, lightness) color space. Translation into the optical retardation (*r*) by comparison with a Michel-Lévy chart with reliable color representation^50^ follows. Since hue values cannot express different shades of gray, the analysis is only performed for retardation values greater than *r* = 300 nm. Assuming that any variation of the LC core diameter (*d*) due to toluene exposure in the experiment can be neglected, we can then calculate the birefringence Δ*n* (dimensionless) as:

(1) $$\text{Δ}n=\frac{r}{1000d},$$

where we for practical convenience consider the values of *r* in nm and *d* in μm.

It remains to establish *d*, which is not simply equal to the outer fiber diameter as seen in POM. Based on scanning electron microscopy (SEM) investigations of fractured LC-filled fibers of the same type as used in this study, Reyes concluded as a rough estimate that the LC core in non-beaded electrospun fibers with PVP sheath and 5CB core comprises 60%–80% of the total fiber diameter.^49^ We thus estimate that *d* for both the 5CB and E7 fibers equals ∼70% of the outer fiber diameter measured by optical microscopy in the non-beaded sections. We confirm that this is a reasonable assumption by comparing our Δ*n* produced by Equation 1 for unexposed fibers with values reported in the literature. For the unexposed 5CB- and E7-filled fibers at a temperature of *T* = 25°C we obtain $\text{Δ}n_{5CB}=0.193\pm 0.008$ and $\text{Δ}n_{E7}=0.206\pm 0.008$, fitting well with earlier reports of $\text{Δ}n_{5CB}^{litt}=0.197$ and $\text{Δ}n_{E7}^{litt}=0.227$ at *T* = 20°C.^51^ While the match for 5CB is excellent, our slightly lower value for E7 suggests that we may have overestimated *d* slightly for this fiber, yet the discrepancy is fully acceptable. When calculating error bars for the data shown below, any error introduced by the estimation of *d* is not considered, since it is a systematic error affecting all absolute values to the same extent, thus not the relative changes as *c*_*tol*_ is increased.

We are now in a position to provide a fully quantitative depiction of the toluene sensing response. The plot in Figure 4 shows Δ*n* of the 5CB- and E7-filled fibers, respectively, as a function of the *c*_*tol*_ values to which they are exposed. Since the fibers need some time to reach complete response (see the next section for an analysis of the temporal behavior), i.e., to reach the equilibrium state under a new level of toluene exposure, the Δ*n* value at each plotted data point is obtained as an average of all Δ*n* values measured during the last 15 s out of the full 60 s of exposure (see Supplemental information for details).

The LC orientational order parameter *S*—and thus Δ*n*—is theoretically predicted^52^ to follow a power law dependence on *T* of the type *S* = *a* + *b*(*T*_NI_ − *T*)^β^. Mean-field behavior would predict β = 0.5, but experiments indicate that β ≈ 0.25 better describes the real temperature dependence.^52^ Hypothesizing that the primary function of toluene exposure is to depress the clearing point *T*_*NI*_, we therefore fit a function,

(2) $$\text{Δ}n=a+b{\left(T_{NI}^{0}e^{-\frac{c_{tol}}{\sigma }}-T\right)}^{\beta }\;,$$

to our experimental data, where we let $T_{NI}^{0}$ be the clearing point in the absence of toluene exposure and we assume, as a first simple model, an exponential decrease of *T*_*NI*_ with increasing *c*_*tol*_, with a characteristic concentration σ. Fitted to the toluene response data, this function reproduces the data very well (Figure 4), albeit with even lower values of β, on the order of β = 0.1. We fix $T_{NI}^{0}=35.6^{\circ }$C for 5CB while for E7, being a mixture, we release $T_{NI}^{0}$ as a fitting parameter around 58°C, obtaining a value $T_{NI}^{0}=56^{\circ }$C from the fitting. The measuring temperature is fixed at *T* = 25°C. The characteristic concentration σ, representing the sensitivity of the LC to toluene exposure, is similar but not identical for 5CB and E7.

While we focus our work on non-beaded fiber segments, to enable the quantitative analysis, we also make qualitative observations of the response of beads, in particular concerning the loss of birefringence at *T*_*NI*_. We find that the critical *c*_*tol*_ value to clear the LC is similar in and between beads, at least for bead diameters no larger than twice that of the unbeaded segment. For lower *c*_*tol*_, during which the LC is in the nematic state, the bead response is complicated by the twist and, most likely, topological defects related to reversal of twist handedness.^53^, ^54^, ^55^ A detailed investigation of this response will be a topic for future studies.

As discussed later, the underlying principle of the fiber response is not specific to toluene but may occur in comparable manner in the presence of other VOCs. A few simple experiments with vapors of isopropanol (Note S3) and cyclohexane (Note S4), respectively, on 5CB-filled fibers qualitatively show that both gases initiate a color change following the Michel-Lévy chart, as toluene does. However, isopropanol dissolves the PVP sheath, leading to leaking out of the LC filling.

### Temporal aspects of the response and test of repeatability with an individual fiber

**Figure 5:**
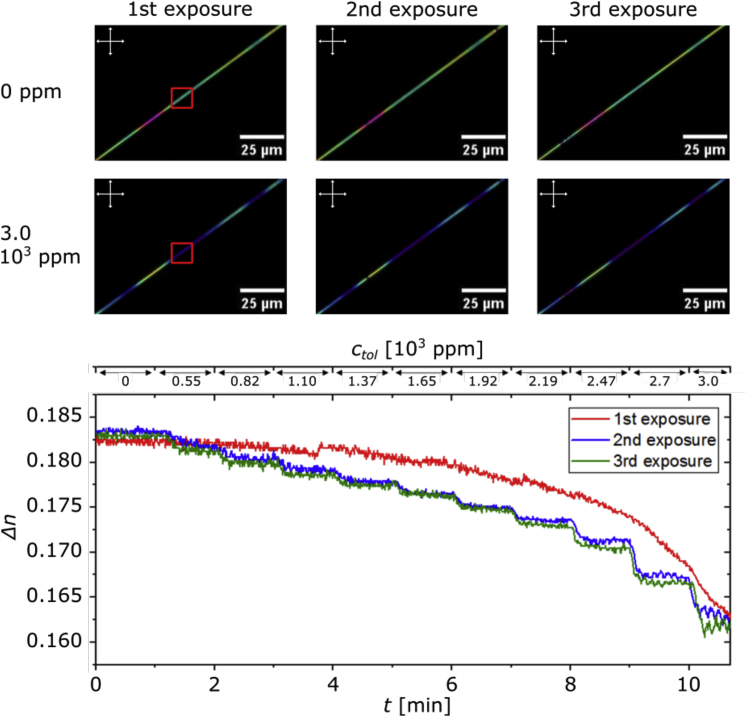
Response during repeated VOC exposures (Top) An E7-filled fiber exposed to increasing *c*_*tol*_ (accuracy, ∼5%) during three consecutive experiments, viewed between crossed polarizers. After each run, the sample chamber is flushed with pure nitrogen for 1 min, followed by ∼1.5 h of pause before the next run is initiated. Images are extracted from videos of each entire experiment and show, for each run, the fiber appearance prior to exposure or during pure nitrogen flow, and at *c*_*tol*_ = 3.0 × 10^3^ ppm, respectively. (Bottom) Δ*n* of the segment marked with a red rectangle in the images above, plotted against time *t* while every minute *c*_*tol*_ is increased in discrete steps (the values are indicated along the top). In the segment, the outer fiber diameter is 5.3 ± 0.5 μm.

As mentioned in the previous section, the vanished LC birefringence reappears when removing toluene from the atmosphere, something we accomplish by flushing the sample cell with pure nitrogen. To test whether the response to toluene exposure is quantitatively reproducible, two individual fibers filled with 5CB and E7, respectively, are exposed to increasing *c*_*tol*_ during three consecutive experiments, with nitrogen flushing in between (see Supplemental experimental procedures for details). A fiber filled with E7, shown in Figure 5 during three consecutive runs at *c*_*tol*_ = 0 and at *c*_*tol*_ = 3.0 × 10^3^ ppm, respectively, here serves as an example. The experiment is repeated twice for each LC type, yielding similar results regarding repeatability.

A first comparison of the POM pictures does not show any major differences between the responses of the individual runs: Δ*n* ≠ 0 reappears after nitrogen flushing, and the fiber in the absence of toluene always shows the same color as before the first exposure. However, looking at Δ*n* plotted over time *t* (measured in the segment marked with a red rectangle in the images from the first exposure), the first run clearly differs from the following ones. While there is a continuous, smooth decrease in Δ*n* throughout the entire first run (the small jump after 3.7 min is most likely an artifact from the conversion from *R*, *G*, *B* color representation into Δ*n*) despite the fact that *c*_*tol*_ is increased in discrete steps, the decrease in Δ*n* mimics the steps in *c*_*tol*_ during the second and third runs.

Table 1 expresses this phenomenon in numbers and allows comparison between the runs. We define a response time (*t*_*r*_) as the time needed after a step change in *c*_*tol*_ for the fiber to go from 10% to 90% of its saturated equilibrium response at the new *c*_*tol*_ level. With this definition we cannot determine *t*_*r*_ for the first run, because it is much greater than the 60-s step length over which *c*_*tol*_ is kept constant, i.e., the response does not reach 90% of saturation before *c*_*tol*_ is changed. In contrast, *t*_*r*_ is well within the step window during runs 2 and 3, and it remains nearly unchanged between these runs. To find their respective 10% and 90% levels, we assume that the last 15 s of the full minute of exposure to constant *c*_*tol*_ shows the equilibrium state, thus defining the level corresponding to 100% response. Additionally, a single experiment on a 5CB-filled fiber exposed to toluene vapor suggests an even higher repeatability of at least 10-fold after pre-exposure (see Note S5).

**Table 1:** Response time as function of repeated exposure

$\text{Δ}c_{tol}$ [$10^{3}$ ppm]	$t_{r}^{1}$ [s]	$t_{r}^{2}$ [s]	$t_{r}^{3}$ [s]
0.82 → 1.10	>60	14 ± 2	12 ± 2
1.65 → 1.92	>60	19 ± 3	17 ± 3
2.47 → 2.7	>60	12 ± 2	10 ± 2

Response times $t_{r}^{i}$ of the E7-filled fiber shown in Figure 5 for three representative step changes in *c*_*tol*_ and three consecutive repeated exposures, numbered *i* = 1, 2, 3. Concentration values are given with accuracies of ∼5%.

## Discussion

By mapping the variation in Δ*n* as a function of *c*_*tol*_, we not only turn the LC-functionalized fibers into true continuous sensors, capable of quantifying the concentration of a VOC to which they are exposed, but we also demonstrate that the range in *c*_*tol*_ over which the fibers respond depends greatly on the clearing point, *T*_*NI*_, of the LC used. Since E7 to 50% consists of 5CB, and since the other three components (7CB, 8OCB, and 5CT) are chemically very similar, the difference in response behavior is unlikely to reflect different specific molecular interactions between LC and analyte. Likewise, the fact that both fiber types are spun following identical procedures under identical conditions, using the same polymer, and as both fiber samples appear with very similar morphology during POM investigations, we consider any impact of the fiber sheath and overall fiber morphology on the response differences to be negligible. We can also rule out that temperature variations during exposure would have impacted our observations, as our sensing experiments are performed under constant gas flow with the fibers contained in a specially designed cell with temperature control, maintaining the fibers at *T* = 25°C.

The higher *T*_*NI*_ of E7 compared to 5CB is often said to mean a greater stability of the nematic order, and it is this that gives rise to the different response range: toluene dissolving into the LC acts as an impurity that disturbs the long-range orientational ordering of the nematic phase, required for birefringence. The more impurity is added, the more *T*_*NI*_ is depressed compared to the pure LC. Since *T*_*NI*_ of 5CB is near room temperature already for the undisturbed LC, the clearing point is brought close enough to room temperature already at the low (compared to E7) toluene exposure level of *c*_*tol*_ ≈ 0.6 × 10^3^ ppm, and with *c*_*tol*_ = 1.50 × 10^3^ ppm, *T*_*NI*_ is reduced to 25°C, thus clearing the core material at the temperature of our experiments. Even if the relative impact of toluene exposure is comparable with E7, the starting point there is a more robustly ordered nematic, requiring much greater *c*_*tol*_ to bring down the clearing point far enough from the original *T*_*NI*_ ≈ 58°C to observe an effect at a temperature of 25°C.

Future extensions need to carry out the quantitative gas exposure experiments as a function of temperature, in order to produce a richer set of data that allows reliable fitting of β and σ in Equation 2, and testing whether an exponential decay of *T*_*NI*_ with increasing *c*_*tol*_ is appropriate or if another function better describes the response. However, the excellent fits in Figure 4 strengthen the hypothesis that the effect of the toluene is primarily to suppress the clearing point. An interesting conclusion from these observations is that an LC core with lower *T*_*NI*_ than that of 5CB would give fibers that respond to lower VOC exposure levels, reaching truly competitive sensor performance. The regulatory permissible exposure limit (PEL) for toluene given by the Occupational Safety and Health Administration (OSHA) of the US for an 8-h time-weighted average (TWA) is 200 ppm, with 300 ppm as an acceptable ceiling concentration.^56^ Recommended limits are half as high. PELs released by the European Commission (Commission Directive 2006/15/EC^57^) are even lower, giving 50 ppm for TWA and 100 ppm for short time exposure. Setting *a* = 0, *b* = 0.15, β = 0.1, and σ = 3.5 × 10^3^ ppm in Equation 2, in line with our fitting results, we can estimate that the response threshold of LC-functionalized fiber sensors would meet such criteria at *T* = 25°C if an LC mixture with *T*_*NI*_ ≈ 28°C is used.

The problem with this strategy of working with *T*_*NI*_ near ambient temperature is that the temperature window of operation becomes very narrow: on a warm day the LC may have cleared even without any exposure. To exploit this opportunity one would thus need to combine multiple LCs in a single sensor. A useful strategy would be to combine patches of mats spun with different LCs in the fiber cores, the different *T*_*NI*_s of which cover the full operation temperature range with relatively small intervals. Since LC mixtures can be used (as demonstrated by the successful use of E7 as core), such tuning of *T*_*NI*_ is easily achieved, with commercially available and quite low-cost materials. In this way, we could ensure that one subset mat is always at operation conditions that ensure high sensitivity regardless of temperature. Since the nematic-isotropic transition is fully reversible, the compound sensor could be continuously in use as the temperature varies throughout the day, throughout the year, or in zones of operation with varying climate.

A garment-integrated sensor of this type would typically be extended along one dimension, with a temperature axis along it that guides the user to the right patch to observe. Even better, if the nematic core LCs are exchanged for suitably adapted cholesteric mixtures, the temperature responsiveness of the cholesteric reflection color^40^ could dynamically provide this kind of guidance, while simultaneously rendering the use of POM for readout unnecessary. We will come back to the latter aspect below.

The strong reduction in response time *t*_*r*_ after the first toluene exposure run in Figure 5 shows that the exposure to toluene introduces structural or compositional modifications to the fiber sheath that are irreversible on the timescale of the full experiment. Interestingly, these modifications are highly beneficial, because they are apparently completed after the first exposure, ensuring repeatable behavior during subsequent experiments, and they do not negatively impact the response. Quite on the contrary, they lead to a dramatically faster response, reducing *t*_*r*_ from minutes to seconds. What could these sheath changes be and why do they have such beneficial impact? Toluene is a poor solvent for PVP, unable to dissolve the polymer used in our study ($\bar{M_{w}}\approx 10^{6}$g/mol). However, the interactions are still favorable enough for PVP to form a gel when immersed in liquid toluene (see Figure S2). In our experiments, we can thus assume that toluene condensing on the fiber sheath swells the PVP. As the exposure level increases, more and more toluene is incorporated, until the sheath approaches a state of a compact gel. Above a certain threshold we may expect channels of toluene that connect the outside atmosphere with the LC core.

While purging the fibers with pure nitrogen, toluene fully leaves the LC core, within experimental accuracy, as suggested by the fact that we recover the pristine optical properties. It is more difficult to know whether also the PVP sheath is free of toluene, as complete drying of solvent-swelled polymers is a slow process. However, here we have a very thin layer of PVP with a high surface-to-volume ratio, exposed to a rather poor solvent, and hence we do think that most of the toluene leaves also the sheath. If the removal of toluene is sufficiently fast, the polymer may have no chance to relax from its swelled state, leaving nanoscopic pores throughout the sheath, which would enable much faster access of toluene vapor to the LC core during subsequent exposures. As a reference, extraction of the 10% by mass gelled PVP in Figure S2, and drying at room temperature overnight in a desiccator left a stiff, porous PVP film. If a similar porous structure would prevail in the sheath of the toluene-exposed fibers, it would explain the much reduced *t*_*r*_. Future experiments should test this by high-resolution electron microscopy on fibers before and after exposure.

Another explanation may be in a change in sheath composition rather than structure. In our previous work,^23^ we found that E7-filled PVP fibers liquefy completely at *T* ≈ 73°C, suggesting a much reduced glass transition temperature *T*_g_ compared to that of pure PVP fibers, which do not lose their shape even at 200°C. This means that E7 can enter the sheath, functioning as a plasticizer and softening the PVP considerably. By swelling the sheath with toluene during the first gas sensing experiment, additional LC is likely to mix into the sheath, remaining there even after the toluene has evaporated. This then softens the sheath further and also increases the solubility of toluene during subsequent exposures, which would be another way of explaining the reduced *t*_*r*_. A differential scanning calorimetry (DSC) analysis of pristine mats and mats exposed once to toluene may be a good way of testing this hypothesis: if the first toluene exposure leaves the PVP mixed with more LC than in the pristine fibers, *T*_*g*_ should be reduced.

Long-term studies are needed to test whether the dramatic reduction in *t*_*r*_ is transient, which may be the case if toluene remains in the PVP sheath between runs, or permanent, which would be expected if the sheath is free of toluene but rendered either porous via rapid solvent evaporation, or plasticized with additional LC. Support of the change being permanent, with no toluene remaining, is given by the fact that the original fiber appearance was recovered immediately after 1 min of nitrogen purging, with identical appearance 1.5 h later, when the next run was started.

**Figure 6:**
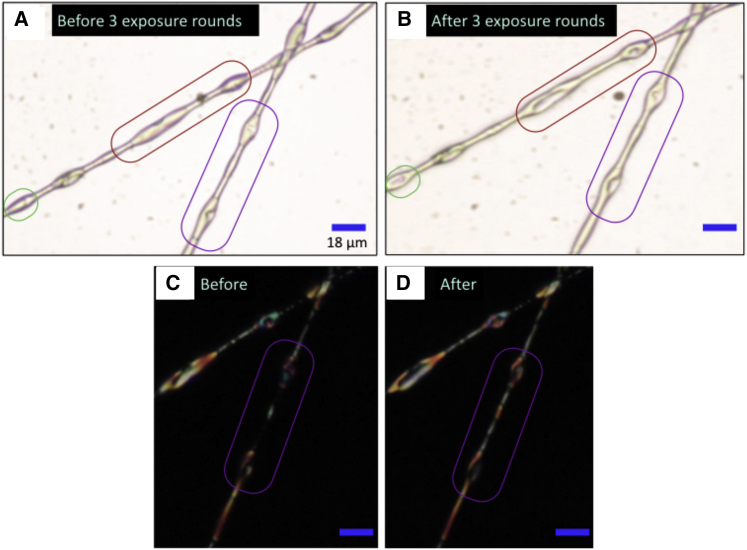
Irreversible changes to PVP sheath upon toluene exposure Two beaded 5CB-filled fibers with discontinuous cores before (A) and immediately after (B) three rounds of toluene exposure. The red and purple outlined regions in (B) show areas where the fiber sheath deflates after toluene exposure. Additionally, LC moves from beads to non-beaded regions, as seen with the fibers between crossed polarizers in (C) and (D).

Moreover, we have confirmed long-term impact on the PVP sheath of toluene exposure on a microscopic scale, by studying the morphology of discontinuously filled fibers with strongly beaded character by POM before and after repeated toluene exposure (see Figure 6). We show the exact same fiber regions without (A and B) and with analyzer (C and D), clearly revealing irreversible shape changes in the beads in the form of depressions that are large enough to detect optically. Comparing C and D, we note that the region between the beads has more uniform LC filling after the toluene exposure. These two effects suggest that the PVP sheath was softened during toluene exposure, as expected during solvent swelling, allowing capillary forces from the LC to suck LC from the beaded regions to cavities in the cylindrical ones, giving rise to the depressions in the beads and filling the fibers more uniformly. The effect on the sheath of the initial exposure to toluene suggests that fibers intended for use in gas sensing should always be pre-exposed to any vapors with which they may come into contact and which may swell the polymer.

A simple way of bringing down the clearing point of a nematic LC is to add a chiral dopant, which forms an isotropic liquid on its own at room temperature, such as the commonly used 5CB-related molecule CB15. The chiral dopant has the additional benefit of turning the nematic into a chiral nematic, or cholesteric, phase, inducing a helical modulation of the orientational order. With enough chiral dopant, the pitch of the helix can be short enough to give rise to visible Bragg reflection, rendering the mixture strongly colored. By mixing a chiral dopant such as CB15 with a high-*T*_*NI*_ mixture such as E7, one can easily achieve a cholesteric mixture that reflects visible colors across a wide temperature range, and also tune *T*_*NI*_ accordingly. This will thus be an interesting path to explore, on the one hand bringing down *T*_*NI*_ enough to get highly sensitive response to VOC exposure, and on the other hand switching from a response amounting to a change in Δ*n*, which normally requires a POM for analysis, to a change in reflection color, which could be detected by the naked eye. The ideal situation is one where the helix pitch varies from deep red to deep violet in the temperature range just below *T*_*NI*_, because that might provide a sensor mat segment that both indicates when the temperature is appropriate for it to be used as a VOC sensor, and a VOC response that is easily readable to the naked eye.

We see two further alternatives to meeting the crucial requirement of removing the dependence on a POM for the analysis. First, one can use the classic “guest-host” principle^58^ of colored LCDs (common, e.g., in railway stations) in which a dichroic dye is mixed into a nematic LC host. The appropriate dye has a mesogen-like shape, such that it is aligned along the LC director, maximizing its absorption and thus the color effect when light passes through the fiber. If an analyte causes a transition to the isotropic phase, the alignment control is lost, and the color intensity should decrease, giving a response that can be visible to the naked eye. With a smart combination of dichroic dyes,^58^ the effect may be enhanced to give strong contrast. The second possibility might be to make a layered composite that encompasses the key elements of the POM setup that we are using here, but in a wearable format. While this is clearly more challenging, linear polarizers have been made by electrospinning,^59^ and hence the LC-functionalized fibers could be placed between two such sheets of polarizing fibers to create the crossed-polarizer geometry, while still ensuring good contact with the air in order that the VOC can enter the fibers. The lamp of the POM would most naturally be replaced by ambient light, and the detector and colorimetric analysis could be conveniently carried out by using organic photovoltaic cells on the inside of the composite structure: Kang et al.^60^ demonstrated a self-powered electronic sensor of NO_2_ by sandwiching a film that changes its transparency in response to NO_2_ exposure over an organic photovoltaic cell, tuned for operation in the wavelength range of maximum response of the film. The same principle might be used to turn the variations in Δ*n* of our LC core into an electrical signal, fueled by ambient light.

The next critical issue is to add selectivity to the response. Since the response we are dealing with here is simply related to the action of a VOC as impurity in the LC, reducing the degree of orientational order, any solute with this effect (e.g., acetone, chloroform, ethanol) will have a similar impact, as demonstrated qualitatively on the examples of isopropanol (Note S3) and cyclohexane (Note S4). To bring in selectivity, we thus need to mix in an additive that reacts specifically to a certain analyte in such a way that its interaction with the LC changes, causing the phase to change in a way that can easily be detected in the presence of this analyte. A powerful choice is the already mentioned chiral dopant that turns a nematic host phase into a cholesteric exhibiting selective reflection, if a chiral dopant is identified that changes its helical twisting power (HTP) when exposed to the target analyte. Several teams have demonstrated this principle, using such cholesteric mixtures to selectively detect CO_2_, NO_2_, O_2_, and H_2_O.^22^, ^23^, ^24^^,^^61^ On its own, this may not bring the desired high sensitivity, since the analyte-induced change in HTP is a bulk effect, and even for samples with high surface-to-volume ratio such as fibers, the required VOC concentration may be high. However, as the HTP is dependent not only on the dopant but also on the host, not least on the elastic constants, which are strongly related to its orientational order, it may be possible to combine this approach with a mixture that has low clearing point, in order to get high sensitivity and selective response simultaneously.

One of the most successful approaches to achieve selective and sensitive VOC response with LCs is to use a VOC-responsive aligning layer.^12^^,^^32^^,^^33^ If this layer changes its influence on the LC, imposing tangential rather than normal alignment or vice versa, upon exposure to a certain analyte, this can give a strong optical response at very low VOC concentration, since only the bounding surface needs to be saturated, not the bulk LC. This approach may be transferred to LC-filled fibers if the specific aligning agent (for instance perchlorate salts^32^^,^^33^) can be made to aggregate at the interface between the polymer sheath and LC. Alternatively, a simpler approach may be to make use of the VOC-induced change in the order parameter studied in this paper in combination with an alignment layer that changes its aligning impact as the LC order parameter reduces upon approaching the clearing transition. We recently demonstrated such an effect using spherical shells of nematic 5CB and E7, stabilized against aqueous phases using the amphiphilic block copolymer Pluronic F-127.^62^ While the alignment is tangential deep in the nematic phase, it turns normal as the nematic-isotropic transition is approached. Spinning LC-filled fibers with a block copolymer such as F-127 incorporated in the sheath, or adsorbing at the LC-sheath interface, may be easier than incorporating inorganic salts, as the F-127 can be co-dissolved with the sheath polymer. With a color-reflecting cholesteric LC core, the change from tangential to normal alignment is easily recognized by eye, since only tangential alignment provides the geometry that yields color reflection. Also, with modifications of the amphiphilic block copolymer structure that incorporate analyte-responsive moieties, a specific response might be achieved.

Finally, a critical requirement is to replace the PVP in the sheath of the fibers studied here with a better suited polymer. PVP is highly humidity sensitive and mechanically delicate, precluding the use of PVP sheath fibers in garments. The reason that it is frequently used in LC-core fiber spinning is that it is easy to spin fibers with ethanol-dissolved PVP around LC cores such as 5CB, while other polymers, dissolved in other solvents, are much more challenging to spin with good LC filling. In a parallel research thrust we are currently exploring a method to spin LC-filled fibers with a polymer sheath that can be crosslinked to achieve stability. Using carefully selected solvents and polymers we obtained very promising results, which will be reported separately. Once fibers can be spun that have a sufficient filling of LC yet use a polymer sheath that is robust enough to incorporate into garments, it remains to be seen whether the pre-exposure to the gaseous analyte that had such a beneficial effect in increasing response speed can still be used. Overall, the impact of variations of the air quality apart from VOCs, in addition to humidity also including issues such as suspended particulates, needs to be investigated in future studies. While there are thus many challenges that remain to be addressed, we hope this study also shows that there are many promising routes to achieve wearable VOC sensors based on LC-functionalized fibers that may find practical use.

## Experimental procedures

### Resource availability

#### Lead contact

Further information and requests for resources and reagents should be directed to and will be fulfilled by the lead contact, Jan P. F. Lagerwall (jan.lagerwall@lcsoftmatter.com).

#### Materials availability

This study did not generate new unique materials.

### Materials

To produce a suitable homogeneous polymer solution for the fiber sheath, 12.5 wt% PVP ( $\bar{M_{w}}=1.3\times 10^{6}$g/mol; Sigma-Aldrich) was dissolved in ethanol (≥99.8%; Merck) by stirring overnight at room temperature. The nematic LC 5CB was obtained from Yantai Xianhua Chem-Tech, and the nematic LC mixture E7 was obtained from Synthon Chemicals. Gas bottles of premixed toluene test gas (216 mol ppm in nitrogen) and of pure nitrogen, respectively, were purchased from Messer. Additionally, liquid toluene (≥99.5%; Merck), cyclohexane (Merck), and isopropanol (Merck) were used.

### Electrospinning setup and parameters for fiber production

**Figure 7:**
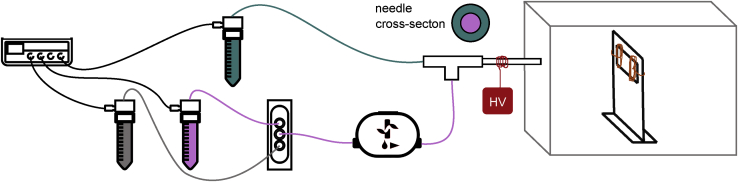
Electrospinning setup Schematic sketch of electrospinning setup with coaxial needle for production of LC-filled PVP fibers. A microfluidics pressure control unit is used to pump PVP solution (turquoise), LC (pink), and/or ethanol (gray, for cleaning) through the system. High voltage (HV) is applied to the spinneret’s needle and allows fiber formation on a grounded collector, placed in an insulating chamber. Images from Fluigent are used with permission.^63^

Figure 7 is a schematic representation of the electrospinning setup, with a microfluidic pumping system and an insulating chamber of polymethylmethacrylate (PMMA), containing the coaxial spinneret (see Supplemental experimental procedures) and collector. The latter had two ring-shaped copper wires connected to ground, fixed to a PMMA holder, allowing the collection of free-hanging fibers. The metallic outer needle of the spinneret was connected to the positive pole of the high-voltage power supply (Gamma High Voltage ES30P-5 W/DAM) to apply voltages between 7.75 and 8.5 kV. The distance between the needle tip and collector was 10 cm. To flow the LC (5CB and E7, respectively) and PVP solution, specially designed reservoirs (Fluigent P-CAP and Fluiwell-4C) were pressurized using a pressure control unit (Fluigent MFCS-EZ, maximum pressure 1,034 mbar) to 700–750 mbar for LC and 700 mbar for PVP solution. The resulting flow rate *u* of the LC was measured using a microfluidic flow control unit (Fluigent Flow Unit M), yielding 18–38 μL/min. For the polymer solution, the flow rate was determined to ∼18 μL/min by an external calibration (see Supplemental experimental procedures for details). Furthermore, a two-way bidirectional valve (Fluigent, 2-SWITCH) allowed easy, half-automated cleaning of the setup after use. Temperature (22.5°C–23.5°C) and relative humidity (31%–40%) were monitored by a thermo-hygrometer (TFA Dostmann 30.5002), and a digital camera (Zarbeco Z505-OR2) with a macro lens monitoring the Taylor cone. The whole setup was placed in a fume hood.

### Gas sensing setup and experimental procedure

**Figure 8:**
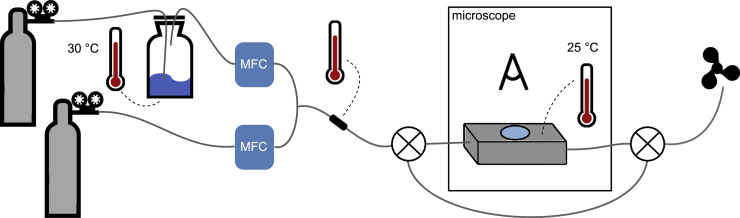
Quantitative VOC sensing setup Sketch of the setup used to monitor the fiber response to different concentrations of VOC. Concentrations are adjusted by dilution of a stream of VOC vapor with pure nitrogen. Mass flow controllers (MFCs) allow to set the flow of the two streams, and a temperature-controlled gas washing bottle filled with liquid VOC is used to yield higher concentrations. The fiber sample is placed in a temperature-controlled holding cell and can be observed with a POM using transmitted light. The temperature of the gas mix can be measured before entering the cell, and a bypass with two switching valves allows leading the stream directly to the fume hood without passing the sample if needed.

The gas sensing test setup is shown in Figure 8. The fiber sample was placed on a glass slide in a custom-built, gas-tight holding cell of aluminum (see Supplemental experimental procedures), with windows on top and bottom allowing optical monitoring of the response to VOC exposure at varying concentrations. The cell was placed between crossed polarizers of a POM (Nikon LV100, operating in transmission mode) with a digital camera (Canon EOS 706) attached for recording. A working temperature of 25°C was assured using an electronic temperature sensor and two heating resistors fixed to the cell.

Different concentrations of toluene vapor were realized by controlled dilution of a flow of nitrogen carrier gas with known toluene concentration with pure nitrogen. To this end, mass flow controllers (MFCs; MKS, models GM50A013502MAV020 and GE50A013502MAV020) were used to adjust both individual flows, always maintaining a constant overall flow. In order to reach higher concentrations than that of the pre-mixed toluene test gas, a gas washing bottle filled with liquid toluene was placed in between the test gas bottle and the corresponding MFC. The gas washing bottle was placed in a water bath at 30°C for constant evaporation conditions. Before the mixed gas stream entered the sample cell, the temperature was measured and a valve allowed to decide if the gas should pass through the sample or be led into a bypass ending in the fume hood. A photo ionization detector (PID; AlphaSense PID-A1, 10.6 eV lamp) functioned as concentration control during the calibration of the system (see Supplemental experimental procedures for more details), in combination with a power supply giving 3.6 V DC and a multimeter (PeakTech 1035) for reading the PID response. For the experiments with isopropanol and cyclohexane, respectively, the test gas bottle was replaced by a second nitrogen gas bottle and the gas washing bottle was filled with the liquid analyte.

The POM appearance of fibers before and during VOC exposure was recorded to video with consistent camera settings for all experiments. After checking for director twists by rotation of the sample, VOC was led through the sample chamber. Starting with 0 mL/min of the toluene-containing gas mixture, its flow was increased in steps of 10 mL/min or 5 mL/min while the flow of pure nitrogen gas was decreased to yield a constant overall gas flow of 180 mL/min. Each ratio was maintained for 1 min. Once the exposure level for LC clearing had been reached, the holding cell was purged with pure nitrogen. The experiments were repeated multiple times with fibers produced from several electrospinning experiments. To check for quantitative repeatability of the response of an individual fiber, the whole procedure was repeated three times. This was done with two fibers for each LC. Gas flows were converted into concentrations using the calibration already mentioned, with the results shown in Supplemental experimental procedures. The experimental videos were analyzed according to Supplemental experimental procedures.

## Data Availability

The authors declare that data supporting the findings of this study are available within the article and in the Supplemental information. All other data are available from the Lead contact upon reasonable request. The only code produced was the MATLAB script for extracting RGB color pixel values from the videos of the gas sensing experiments; this script is included as Data S9.

## Supplemental information

Document S1. Figures S1–S11, Notes S1–S6, Tables S1-S4, and Supplemental experimental procedures

Data S1. Raw data from the analysis of the example 5CB-filled fiber from Figures 2, 3, and 4, including experimental details, the RGB values of a selected fiber segment extracted from the recorded experiment, as well as the conversion into birefringence

Data S2. Raw data from the analysis of the example E7-filled fiber from Figures 2, 3, and 4, including experimental details, the RGB values of a selected fiber segment extracted form the recorded experiment, as well as the conversion into birefringence

Data S3. Raw data from the analysis of response repeatability with the E7-filled fiber, shown in Figure 5 Data of the color analysis and temporal aspects are included.

Data S4. Generation of reference birefringence values from the Michel-Lévy chart See Supplemental experimental procedures for details.

Data S5. Raw data from calibration of 5CB flow for electrospinning experiments; three individual calibrations on different days The procedure is described in Supplemental experimental procedures.

Data S6. Raw data from calibration of E7 flow for electrospinning experiments; three individual calibrations on different days The procedure is described in Supplemental experimental procedures.

Data S7. Raw data from calibration of the PVP solution flow for electrospinning experiments; three individual calibrations on different days The procedure is described in Supplemental experimental procedures.

Data S8. Raw data from the gas sensing setup calibration for toluene, including the basic PID calibration and a subsequent calibration of gas flows to PID signal The procedure can be found in Supplemental experimental procedures.

Data S9. MATLAB script for RGB video analysis It was used to generate the RGB values from the video files and is explained in more detail in Supplemental experimental procedures

Document S2. Article plus supplemental information
