# Supplementary material for: Quantitative volatile organic compound sensing with liquid crystal core fibers
Source: Cell Rep Phys Sci. 2021 Dec 22;2(12):100661. doi: 10.1016/j.xcrp.2021.100661 (PMC8724680; doi:10.1016/j.xcrp.2021.100661)
Supplement: Document S1. Figures S1–S11, Notes S1–S6, Tables S1-S4, and Supplemental experimental procedures [file mmc1.pdf]

Cell Reports Physical Science, Volume *2*

Supplemental information

Quantitative volatile organic compound sensing with liquid crystal core fibers

Katrin Schelski, Catherine G. Reyes, Lukas Pschyklenk, Peter-Michael Kaul, and Jan P.F. Lagerwall

Cell Reports
Physical Science

Supplemental Information

Supplemental Figures

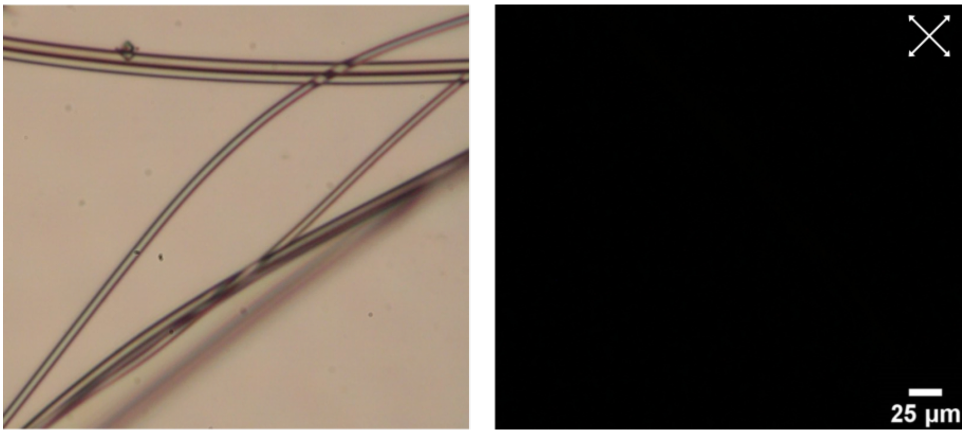

Figure S1: Electrospun fibers of pure PVP, without any core material, imaged using POM, without (left) and with (right) analyzer, related to the statement in the first Results section in the main paper, concluding that the birefringence seen in POM can be attributed entirely to the LC core. In contrast to liquid crystal filled fibers, no birefringence is detected, rendering the photo obtained with crossed polarizers uniformly black. The white scale bar of 25 μm is valid for both pictures.

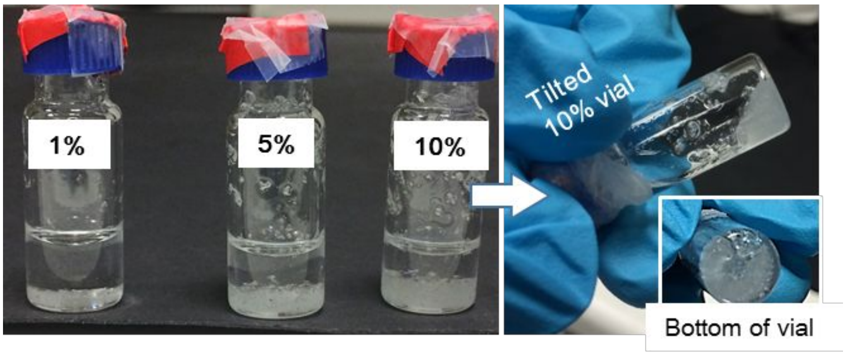

Figure S2: Demonstration of toluene being a poor solvent for PVP, relating to the discussion in the main paper on the impact of the first exposure of the fibers to toluene vapor. Different mass fractions of PVP (molar mass: 10^6 g/mol) in toluene showing that, while PVP is insoluble in toluene, it does gel. In tilting the vial containing the 10 % mass fraction of PVP we see how the gel separates and sticks to the bottom of the container.

Cell Reports
Physical Science

CellPress

Supplemental notes

Note S1 POM investigation of the ground state director orientation within the fibers

This note relates to the statements in the first Results section in the main paper, regarding the orientation of the LC director in beaded and unbeaded sections of a fiber. As demonstrated in Figure S3, the LC-core fibers generally have the director uniformly aligned along the fiber axis, although there can be local variations. In panels A–D, two nearly parallel fibers are viewed between crossed polarizers in a POM while they are oriented approximately $0°$, $45°$, $90°$ and $135°$ to the polarizer, respectively. In every situation, the fibers are photographed once with a first-order λ-plate inserted (left half, pink background) and once without this plate (right half, black background). These fibers were not used in the gas sensing experiments described in this paper, but they were spun under similar conditions with a 5CB core inside a PVP sheath, giving diameters similar to those used in the gas sensing study, hence they are comparable in many respects.

The interference color between crossed polarizers, without λ-plate (right parts of B and D), alternates between a second-order yellow and a first order orange (in the Michel-Lévy chart we may locate the colors at retardations of $r \approx 800$ nm and $r \approx 470$ nm, respectively) between the thinnest straight cylindrical segments and the beads in the fibers. It is interesting that the beads in these fibers show *lower*, not higher, retardation, indicating *less* LC filling than in the sections between beads. We speculate that this is due to the beads being formed via a Rayleigh instability acting on the PVP solution but not on the LC core while spinning these fibers. The beads would then have accumulated PVP, leaving a thinner LC core than in the cylindrical segments between.

The identification with certainty of the retardation values in the thin and thick fiber sections, respectively, is made possible by comparing with the images obtained when the λ plate is inserted in the POM.

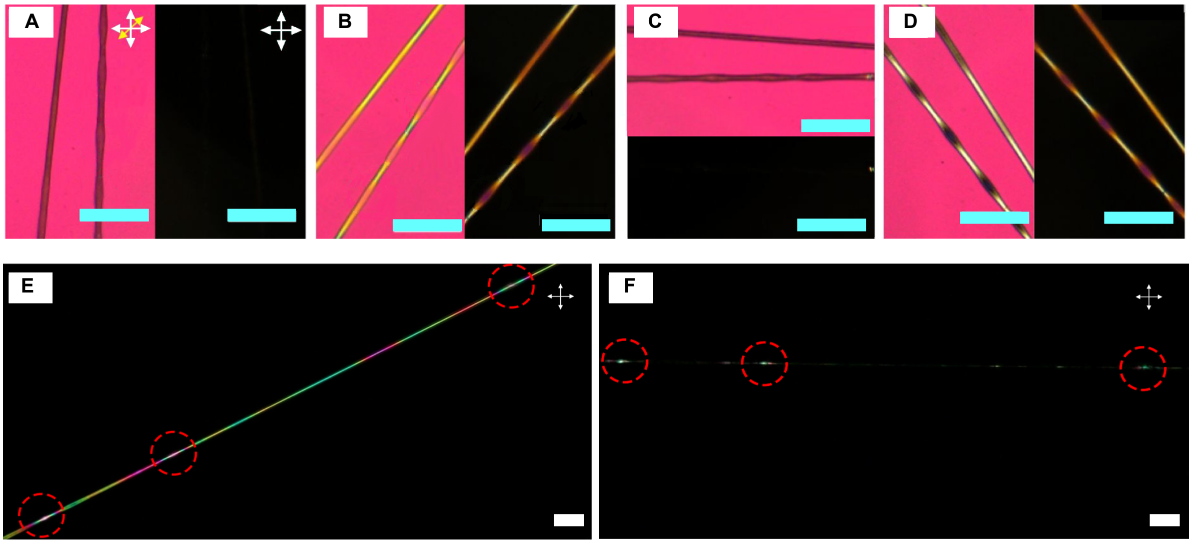

Figure S3: (**A–D**) Close-up POM images of 5CB-core PVP fibers viewed between crossed polarizers with and without a first-order λ plate inserted (pink and black background, respectively). The fibers are oriented 0° (A), 45° (B), 90°(C), and 135°(D) with respect to the polarizer (vertical in the image). (**E–F**) A longer section of a fiber with E7 core is shown between crossed polarizers at 45° (E) and 90° (F) to the polarizer. Scale bars: 20 μm (cyan), 25 μm (white). The optic axis direction of the λ plate is indicated with a yellow double arrow in A. All images are obtained at room temperature (about 25°C).

When the plate is inserted with its optic axis along the fiber axis (left half of B), the yellow segments remain yellow but gain a subtle greenish touch, while the orange beads turn pinkish orange. These shifts are expected for an additional retardation of $r = 551$ nm provided by the λ-plate: comparing with the Michel-Lévy chart we find the same colors at $r \approx 1350$ nm and $r \approx 1020$ nm, respectively. When the fiber is instead oriented perpendicular to the optic axis of the λ–plate, as in the left half of D, the originally yellow straight regions appear white and the originally orange beads are now almost perfectly dark. Inserting the λ-plate with its optic axis perpendicular to the LC director means that we subtract $r = 551$ nm from the original retardance, taking us to $r \approx 250$ nm and $r \approx -80$ nm between and in the beads, respectively. The Michel-Lévy chart is symmetric about $r = 0$, i.e., the color at $r = -80$ nm is the same as at $r = 80$ nm, thus dark grey. At $r = 250$ nm we find an almost colorless white, hence the comparison with the Michel-Lévy chart under the assumption that the director is along the fiber axis perfectly reproduces the colors seen in the fibers. We can thus conclude that the director aligns predominantly along the fiber axis throughout the fiber, and that the beads in these fibers are indeed deprived of LC compared to the unbeaded sections, although the fiber is thicker externally at the beads.

Panels E and F show an E7-filled fiber from the series that was used in the gas sensing experiment, at 45° and 90° angle with respect to the polarizer. This fiber shows a slightly different behavior in the regions with beads. While the fibers in A–D become uniformly dark when they are aligned along or perpendicular to the polarizer, the fiber in E–F has several small regions which remain bright also in F, when the fiber is perpendicular to the polarizer (thus along the analyzer). No matter how the fiber is oriented, these regions (highlighted by red circles) never go entirely dark. This demonstrates the existence of a small twist of the director field in these regions, ensuring that there is always at least part of the core in which the optic axis is neither parallel nor perpendicular to the polarizer.

In Fig. 2 in the main paper, the same type of fiber is shown both between crossed polarizers and without polarizers, and the comparison reveals that the twisted regions are found near beads in the fiber. In contrast to the beads in Figure S3A–D, the core is thicker in these beads, as can be seen by following the sequence of color between crossed polarizers going from the unbeaded to the beaded part and comparing with the Michel-Lévy chart. As a tentative explanation of the difference, we propose that in this case, the Rayleigh instability started acting primarily on the core fluid, leading to accumulation of LC in the beads. Since we need to measure the birefringence in the fiber as a function of toluene exposure, we must ensure that the director field is uniform, not twisted, where we measure. For this reason we avoid the beaded regions during all quantitative gas sensing experiments.

Note S2 Rare cases of toluene exposure-induced irreversible change to the optical properties of LC-core fibers

This note relates to the statement in the second Results section of the main paper, that the vast majority of fibers have intact LC filling and recover their original optical characteristics after VOC exposure and subsequent purging with inert gas, e.g., nitrogen. During our gas sensing studies, three fibers with 5CB core stood out from all others in showing an irreversible change in LC texture after the first toluene exposure. One of them is shown in Figure S4, before and after the first toluene exposure, up to c_{tol} concentrations sufficient to clear the LC. As is easy to see, the birefringence came back after the experiment, but the texture has changed completely, showing a speckled grey-white pattern rather than the smooth colorful texture seen prior to exposure. Since this behavior was very rare, the three fibers where it was observed were clearly malformed, and we speculate that the sheath may have ruptured, allowing the LC filling to partially leave the core during the phase transition. We did not investigate this behavior further, but it can be noted in the left panel of Figure S4 that the fiber has unusually strong color variations, both in magnitude (all colors from violet to red can be seen) and in frequency (maximum length of constant color is on the order of only 10 μm). It may be that the LC filling was too great in this fiber, giving rise to a relatively strong Rayleigh instability impact within the core and rendering the sheath exceptionally thin, thus providing too weak containment.

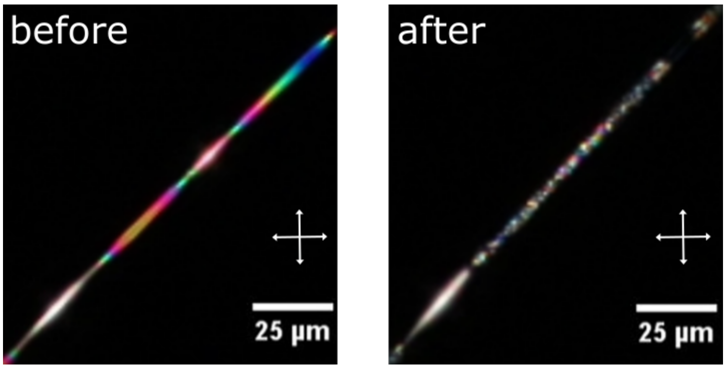

Figure S4: A malformed fiber filled with 5CB before and after exposure to toluene vapor. The concentration of toluene was high enough to induce complete clearing. After removing the VOC by flushing with pure nitrogen, birefringence came back, but the texture was highly irregular. This is an extraordinary phenomenon observed in only three fibers throughout the full study.

We may conclude that a pre-exposure of all fibers to be used as gas sensors is advisable not only in terms of speeding up the response, as discussed at length in the main paper, but also to reveal any malformed fibers. Any fibers that do not recover their pristine optical characteristics, as seen in POM, should be discarded.

Note S3 Response of 5CB-filled fibers to isopropanol vapor

This note relates to the discussion in the main paper of the response of regular LCs to multiple VOCs that have the impact of reducing their order parameter. In a single experiment, a 5CB-filled fiber was brought in contact with saturated vapor of isopropanol (IPA). This was realized with the same set-up as described in the experimental part of the main paper, but using a gas washing bottle filled with liquid IPA instead of toluene. The applied isopropanol flow (u_{IPA}) was 180 mL/min and was kept constant during the whole exposure time, which is tantamount to a constant concentration of isopropanol (c_{IPA}). As Figure S5 shows, the same color change according to the Michel-Lévy chart can be qualitatively

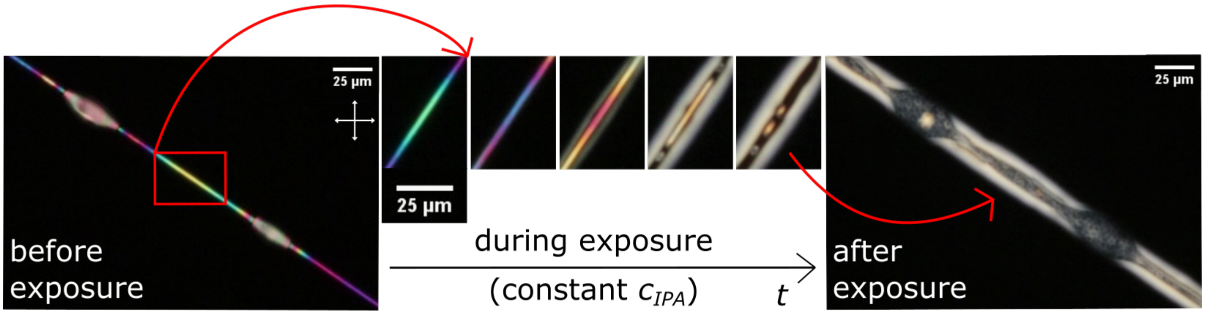

Figure S5: A 5CB-filled fiber between crossed polarizers at $25\ ^{\circ}$C. It was flushed with pure nitrogen gas for 40 s and then exposed to saturated vapor of isopropanol for ≈ 4 min, but without pre-exposure to the analyte. After the fiber core became isotropic, the sample cell was flushed with pure nitrogen again for 50 s. The colorless bright appearance after exposure is due to leaked LC, since PVP is well soluble in isopropanol, leading to an irreversible damage of the polymer sheath. Photos are extracted from a video of the entire experiment and are therefore only snapshots of an actually smooth color transition. The white scale bare of 25 µm of the first small picture is valid for the other ones of same size as well.

Cell Reports
Physical Science

observed during the exposure as for the contact with toluene vapor. However, as time progresses a pool of leaking LC grows along the fiber, appearing bright between crossed polarizers. Since PVP is well soluble in IPA, the polymer sheath is irreversibly destroyed and therefore the fiber does not go back to its former appearance during flushing with nitrogen at the end of the experiment.

Note S4 Response of 5CB-filled fibers to cyclohexane vapor

This note relates to the discussion in the main paper of the response of regular LCs to multiple VOCs that have the impact of reducing their order parameter. Different to isopropanol, cyclohexane does not dissolve the PVP sheath and therefore a more extensive, single experiment was carried out. Again, the former gas sensing set-up was used with liquid cyclohexane within the gas washing bottle and the experiment was carried out according to the procedure described in the gas sensing experimental part. Likewise to the toluene experiments, a 5CB-filled fiber was brought into contact with increasing concentrations of cyclohexane (c_{ch}), realized by different flows of nitrogen and cyclohexane (u_{ch}) under constant overall flow of 180 mL/min. Unfortunately the sensitivity of the PID to cyclohexane vapor is unknown and therefore no absolute values for the applied concentrations can be given. However, a calibration of the u_{ch}/u_{N_2} ratio with respect to the PID signal implies that a linear relationship with positive slope (increased u_{ch}/u_{N_2} increases the PID signal) is given for the full working range. A quantitative examination of the response of LC-filled fibers to cyclohexane vapor of known concentrations will be a topic for future studies, once a suitable PID calibration is available.

As Figure S6 shows, the analyte induces a color change that is qualitatively similar to the other two VOCs tested, ending with a transition from the nematic to the isotropic phase. Like for toluene exposure, complete reversibility is given when the analyte is removed by flushing with nitrogen. Unfortunately, since the actual concentrations are unknown, no statement can be made concerning a higher or lower sensitivity of the fibers to cyclohexane in comparison to toluene gas. It should be pointed out that the overall sensitivity depends both on the response of the LC and on the permability of the polymer sheath for each particular choice of VOC.

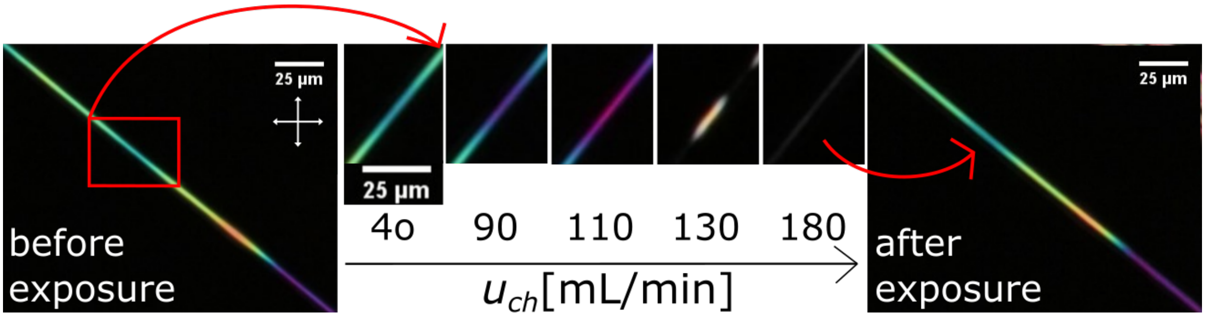

Figure S6: A 5CB-filled fiber between crossed polarizers at 25 °C. Without pre-exposure, it was exposed to different concentrations of cyclohexane vapor realized by increasing u_{ch} and equally decreasing u_{N_2} at a constant overall flow of 180 mL/min. Each flow ratio was held for 1 min, before the next higher ratio was applied (without flushing with pure nitrogen in between). Photos are extracted from a video of the entire experiment and are therefore only snapshots of an actually smooth color transition. The white 25 µm scale bar in the first small picture is valid for the other small pictures as well.

Note S5 Experiment on repeatability with higher number of exposures

This note relates to the assessment of reproducible VOC exposure response, at the end of the Results section of the main paper. To examine if a higher number of toluene vapor exposures still results in the same fiber response, we carried out a single experiment on a 5CB-filled fiber, exposed 10 consecutive times to vapor with $c_{tol} \approx 1.10 \cdot 10^3$ ppm, with flushing steps of pure nitrogen in between. As shown in Table S1, the birefringence of a distinct segment of the fiber decreases during toluene exposure in comparable extent for each repetition, and it fully recovers under nitrogen purging. This can be observed through the POM as a repeatable colour change from green (unexposed) to red (exposed). Response times of these runs lay within the range of 12 s to 21 s. In line with our findings of response time reduction by pre-exposure to the VOC of interest, the fiber was pre-exposed to the analyte; this pre-exposure is not included in Table S1.

Table S1: A single 5CB-filled fiber was exposed 10 times to toluene vapor with $c_{tol} \approx 1.10 \cdot 10^3$ ppm, each time holding for 1 min followed by 3 min of flushing with pure nitrogen gas. An initial pre-exposure step is not included in the number of runs listed. All given birefringence values display an absolute error of ± 0.01.

Exposure	$\Delta n(N_2)$	Δn (tol)	t_r [s]
1	0.22	0.18	18 ± 3
2	0.22	0.18	18 ± 3
3	0.22	0.18	17 ± 3
4	0.22	0.18	18 ± 3
5	0.22	0.18	14 ± 3
6	0.22	0.18	13 ± 2
7	0.22	0.17	12 ± 2
8	0.22	0.17	13 ± 2
9	0.22	0.17	12 ± 2
10	0.22	0.17	21 ± 3

Note S6 Description of video file

This note complements the brief description of the Supplemental Video S1 contained in the main paper. The video shows the response of an E7-filled fiber to increasing concentrations of toluene vapor and serves as an example for visualization of the smooth continuous transition in color as well as the reversibility of the process. The pictures of the E7-filled fiber in 2 and 3 show the exact same fiber, extracted from the full length original video. "Video S1" is an edited, sped up (5 times) and compressed version, starting at $c_{tol} = 1.92 \cdot 10^3$ ppm, which triggers the first visible response. The dark part showing the LC in its isotropic phase ($c_{tol} = 3.8 \cdot 10^3$ ppm) was cut to a sixth of its original duration. Table Table S2 helps to orient in the video, giving the times as they appear in the video at which a certain new concentration is introduced.

Table S2: Times in "Video S1" at which certain concentrations are introduced. Concentration values are given with accuracies of ~ 5 %.

t [min:s]	c_{tol} [10^3 ppm]
00:00	1.92
00:12	2.19
00:24	2.47
00:35	2.7
00:48	3.0
01:00	3.3
01:12	3.6
01:24	3.8
01:26	0

Cell Reports
Physical Science

CellPress

Supplemental Experimental Procedures

Procedure S1 Analysis procedure of the experiment videos by means of the Michel-Lévy chart

This supplemental procedure gives the details on how the color information from experiment videos was extracted and converted into birefringence values, relating to the second Results section and the Experimental procedures section of the main paper. Figure S7a shows the first and second order interference colors of the Michel-Lévy chart plotted as red, green and blue values (R, G, B) versus retardation. These values were obtained by analyzing the chart given by Zeiss [52] using ImageJ Fiji. The triangles are the actual data points that were connected by lines for better visualization. In order to get a single parameter value that can be directly correlated to the optical retardation, the sets of R, G, B values were converted to Hue values (H), using a free to download[1] add-in for Microsoft Excel, producing the H versus r curve in Figure S7b. The conversion works only for the colorful region with retardation greater than 300 nm, because Hue values cannot be used to express gray values. Likewise, r values greater than 750 nm are not shown, as they never occurred during the experiments.

For convenience in fitting a continuous function to the (H, r) data, we added 360 to the H values corresponding to 300 nm$<r<$500 nm. This is valid since hue values lie on a circle, hence H and $H + 360$ describe the exact same point. The advantage of this procedure is that it allows a fitting that contains the whole range of interest. The fitting function determined here is $r = -1.54 \cdot 10^{-9} \cdot H^5 + 1.47 \cdot 10^{-6} \cdot H^4 - 5.34 \cdot 10^{-4} \cdot H^3 + 9.251 \cdot 10^{-2} \cdot H^2 - 8.36 \cdot H + 1059$.

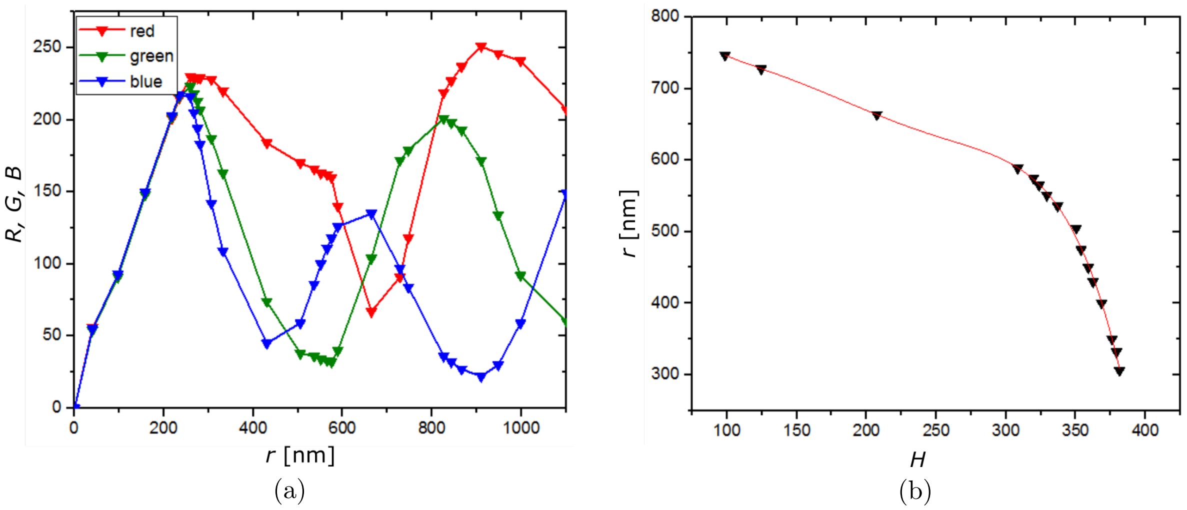

Figure S7: (a) R, G and B values obtained from the Michel-Lévy chart [52]. The triangles represent the actual data points, the points in between not being recorded. For better visualization, the triangles are connected by straight lines, although the real curves are assumed to procede much smoother than how they are presented here. (b) Michel-Lévy chart in hue values (H) for retardation between 300 and 750 nm, obtained from R, G and B values shown in (a). The function $r = -1.537 \cdot 10^{-9} \cdot H^5 + 1.465 \cdot 10^{-6} \cdot H^4 - 5.337 \cdot 10^{-4} \cdot H^3 + 9.251 \cdot 10^{-2} \cdot H^2 - 8.36 \cdot H + 1059$ was fitted to the data. In order to simplify the fitting, $H = 360$ was added for 300 nm$<r<$500 nm (see text).

For the analysis of the videos recorded of the fiber responses to toluene vapor, segments of fibers that show uniform coloring and no twisting in director were chosen. This means that segments must appear dark with 90° intervals during rotation about the vertical microscope axis. ImageJ Fiji was used to measure the diameter of the fiber at the chosen section for five times and the values were averaged. Therefore, POM pictures with no filters inserted were used. A self-written script in MATLAB

[1] https://exceloffthegrid.com/convert-color-codes/#Convert_from_RGB_to_HSL; accessed 11.02.2021.

Cell Reports
Physical Science

CellPress

(MathWorks) allowed to track the color change during the whole video of an experiment. This means, that a rectangular area, labelled by the pixels of its two opposite corners, was marked and for every twelfth frame of the video (∼ 0.5 s), the red (R), green (G) and blue (B) values of each pixel within this zone were measured and averaged. In a few cases, the position pixels of the rectangular box were shifted during the analysis of a single video, in order to chase the segment of interest, which moved because of minor shaking of the unfixed fibers during the experiment. In order to have only one value, R, G and B were converted to H, following the procedure described above. Again, this was done for data with retardations higher than 300 nm only. The function obtained from Figure S7b was used to transfer H into r and in a final step, equation 1 in the main paper was used to calculate Δn of the fibers under toluene exposure. This was plotted both continuously and as a single value for each concentration step. For the latter case, the last 15 s of the exposure time (1 min) were averaged, since this yields a value closer to that of the equilibrium state.

The response time (t_r) of a fiber to toluene vapor is defined as the time needed after a step change in c_{tol} for the optical response to go from 10 % to 90 % of the saturated equilibrium value. In order to calculate it for the individual concentration steps, the raw data were smoothed over 10 points and the averaged value of the last 15 s of the exposure were set to be 100 %, and the saturation value for the previous c_{tol} exposure was set to 0%. However, this was done for pre-exposed fibers exclusively. Since each concentration was held for only one minute and data showed that this is not long enough to reach 90 % of the response during the first exposure, (t_r) was not calculated in those cases but stated to be > 60 s.

Data were analysed using Microsoft Excel (Microsoft), OriginPro (OriginLab) and ProFit (QuantumSoft), the latter two used for producing the graphs shown in the paper.

Procedure S2 Coaxial spinneret geometries

This description complements the brief description of the electrospinning set-up in the Experimental procedures of the main paper. The spinneret for coaxial electrospinning is built up of a polypropylene Reducing T-coupler (Carl Roth; inner diameter: 3.2 mm for longitudinal openings, 1.6 mm for the lateral) with a stainless steel tubing (Unimed, Lausanne, Switzerland; inner diameter: 0.70 mm, outer diameter: 1.10 mm, length: 50 mm) inserted longitudinally into the T-coupler. The exposed end of the steel tubing represents the needle tip and is connected to the high voltage supply. A flexible, 25 cm long silica capillary coated by polyimide (www.bgb-shop.com, Part #: TSP-250350; inner diameter: 250 μm, outer diameter: 360 μm) is inserted through the steel tubing, positioned with its orifice flush with that of the steel tubing. The other end extends from the other longitudinal opening of the T-coupler. All parts are fixed tight with shrinking tubes and polytetrafluoroethylene (PTFE) tape.

The free end of the silica capillary is fitted to a PTFE tube that leads to a Flow Unit (Fluigent; see below). Further PTFE tubing leads to a 2-way bidirectional valve, which connects (again via PTFE tubing) the reservoirs for LC and ethanol, respectively. The total tubing length from LC container to the exit orifice of the spinneret is roughly 36 cm. The lateral opening of the T-coupler is connected to a PTFE tube (length ~ 62 cm) as well, leading to the reservoir of the PVP solution.

All reservoirs are connected to a microfluidics pressure control unit for dynamic flow control.

Procedure S3 Flow calibration

This description complements the brief description of the electrospinning set-up in the Experimental procedures of the main paper. To measure the flow velocity u_{LC} of the LCs during spinning, we used a Fluigent Flow Unit (size M), with a scale factor determined according to the instructions from the manufacturer[2]. The determined scale factor for 5CB and E7 was 0.8 and 0.9, respectively. The calibrated

[2] https://www.fluigent.com/product/microfluidic-components-3/frp-flow-rate-platform/, accessed 01.02.2021]

Cell Reports
Physical Science

CellPress

reference medium of the Flow Unit was isopropanol and for each LC a 3-point calibration was carried out over three days, whilst each pressure (p) level was measured three times. Calculations were based on the densities[3] $\rho_{5CB} = 1.008$ g/mL and $\rho_{E7} = 1.03$ g/mL.

For the PVP solution we avoided using Flow Units for measuring flow velocities, as PVP residues in the Flow Unit can impact its performance negatively. Instead, we resorted to applying the well-known Hagen-Poiseuille equation:

$$u_{PVP-sol} = \frac{p\pi r^4}{8\eta L} \tag{S3}$$

for calculating the flow velocity $u_{PVP-sol}$ of the PVP solution from the applied pressure p (in practice being equal to a pressure difference between the atmospheres on the two sides of the fluid volume). The equation assumes a constant radius r of the channel, a constant viscosity η and a well-defined length L of the channel. While in our system r varies with location, we are not varying any parameters other than the pressure during our experiments (within the flow rate window probed in the experiments, we deem variations in the PVP solution viscosity negligible), hence we simply identify a proportionality constant describing the linear relationship between $u_{PVP-sol}$ and p. To this end, we carry out an external 3-point calibration, measuring flow rates at each p level three times over three days. A certain pressure was applied for one minute and a glass vial was placed over the needle tip to collect the pumped-out PVP solution. It was weighed using a precision balance and $u_{PVP-sol}$ was calculated. Figure S8 shows the calibration curve. While a good linear fit was obtained, a correlation coefficient of R^2= 1 resulting from weighted linear regression, it required having a non-zero pressure-independent contribution. This may at first may seem unreasonable since it would lead to non-zero flow at zero applied pressure. We believe the reason is imperfect sealing in the system for pressurizing the vial, leading to a non-negligible pressure leakage. We thus write the function obtained from fitting as $u_{PVP-sol} = 0.03074 \cdot (p - 86.6)$ which can be considered valid in the range of applied pressures investigated, thus $250 < p < 800$ mbar.

Data were analysed using Microsoft Excel (Microsoft) and further analysis as well as graphing was done with OriginPro (OriginLab).

[3] The source of the density of 5CB was the Sigma-Aldrich safety data sheet for this chemical (found by searching for product #: 328510, product name: 4'-Pentyl-4-biphenylcarbonitrile, CAS: 40817-08-1; accessed 04.02.2021). For E7, the density was taken from Kim, Y.K.; Senyuk, B.; Lavrentovich, O.D. Molecular reorientation of a nematic liquid crystal by thermal expansion. *Nat. Commun.* (2012). https://www.doi.org/10.1038/ncomms2073.

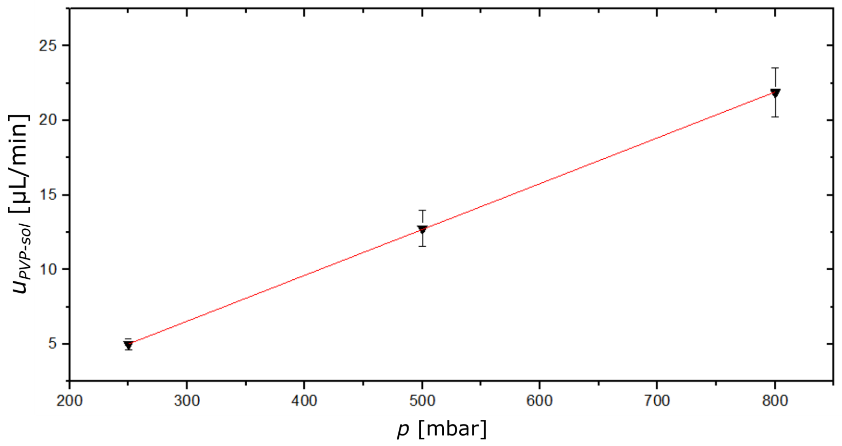

Figure S8: Calibration curve for flow of PVP solution against applied pressure used during electrospinning. Linear regression yields the relationship $u_{PVP-sol} = 0.03074 \cdot (p - 86.6)$.The calibration was repeated on three individual days.

Procedure S4 Holding cell geometries

This description complements the brief description of the gas sensing set-up in the Experimental procedures of the main paper. The custom-built aluminum cell serving as holder for fiber samples during the gas sensing experiments can be seen in Figure S9. It is proven to be gas-tight and designed in a way, that a commercially available glass slide, which is used as substrate for the fibers, fits in perfectly. The inner volume is about 15 mL, including the volume of the in- and outlet. The inset carved in the bottom of the cell is ~ 2.5 mm in height. Removable glass cover slips seal the window on top and bottom such that they can easily be replaced. The photo in Figure S9 was taken before two heating resistors were fitted into the top piece and an electrical temperature sensor on one side of the bottom piece. These additions allowed temperature control of the cell during experiments.

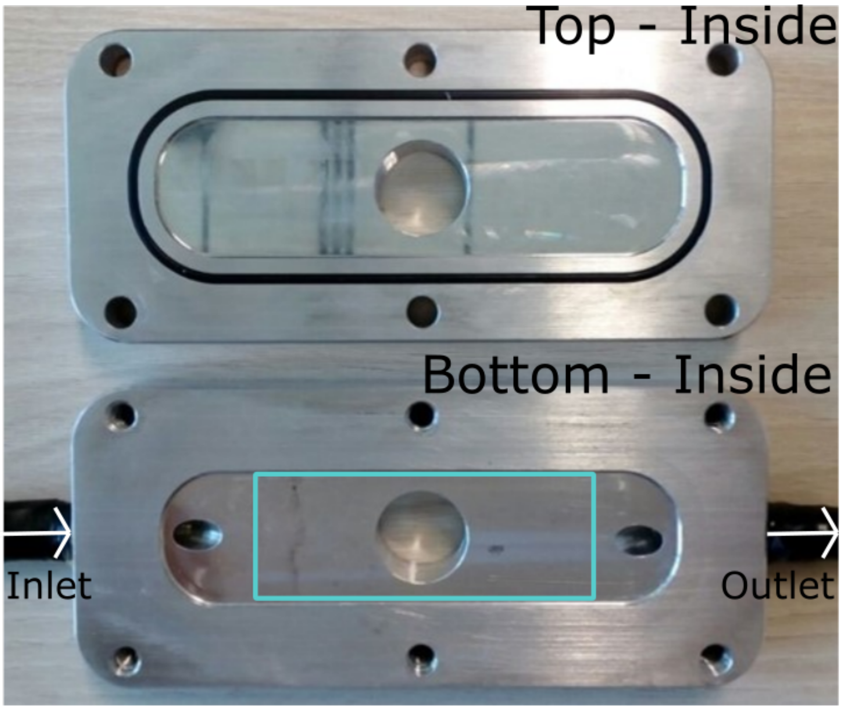

Figure S9: Holding cell for fiber samples during gas sensing experiments. The inside is designed to fit a commercially available glass slide (light blue rectangle) which serves as substrate for fibers.

Procedure S5 Calibration of the photo ionization detector (PID)

This description complements the brief description of the gas sensing set-up in the Experimental procedures of the main paper. As mentioned in the main paper, different concentrations of toluene vapor were realized by dilution of a toluene gas stream with a stream of pure nitrogen, and both flows were adjusted using mass flow controllers (MFC). In this subsection as well as in the following one, all steps needed to calibrate the whole set-up and therefore to allow a translation between toluene-nitrogen-flow ratios and toluene concentration are described in detail and accuracies are discussed. The tubings leading nitrogen only are out of polyurethane (Festo, inner diameter: 5 mm, outer diameter: 6 mm), the remaining ones are of polyamide (Landefeld, inner diameter: 5 mm, outer diameter: 6 mm).

To measure concentrations of toluene, a photo ionization detector (PID) was used. In the first step, the PID response to known concentrations of toluene was checked. Therefore, the testing gas bottle

with 216 ppm of toluene was connected to an MFC directly, and the same was done with a gas bottle of pure nitrogen. The streams were combined behind the MFCs and led through the PID. The baseline signal was determined by measuring the response from pure nitrogen flow (u_{N_2}) for three times a day over six days in total. The flow ratios (u_{N_2} : u_{tol} in mL/min) used for the calibration were 500:0, 400:100, 250:250, 100:400 and 0:500. Since the PID was encapsulated in a chamber of high volume in relation to the preceding tubing system, the reading needed some time (a few minutes) to stabilize. After reaching this point, the PID signal (V, [mV]) was noted every 15 s for a total of 90 s. This whole procedure was repeated over three days. To convert V into toluene concentration (ppm), the baseline signal was subtracted and the result was multiplied, first, by a correction factor (provided by the supplier[4]) of 0.56 specific for toluene, since the PID is originally calibrated to isobutylene, and then by a sensitivity factor.

For the evaluation of accuracies and error propagation, the reading error of the multimeter[5] (Peak-Tech 1035, DCV $\pm$ 0.5 % + 2 dgt.), accuracy of the MFCs[6] (MKS, $\pm$ 1 % of set point for 20 to 100 % of full scale, $\pm$ 0.2 % of full scale for 2 to 20 % of full scale) and standard deviations of the measurements were considered where needed. Table S3 shows that the experimental data fit the expected theoretical values very well. Figure S10 gives the resulting calibration curve of PID signal to toluene concentration with the function $c_{tol} = 0.41745 \cdot V - 29.61528$. Here, the concentration of pure nitrogen flow was assumed to equal zero since this is reasonable from a physical point of view. Because of the slightly different standard deviations of each ratio value, a weighted linear regression was done. However, the weighting does not show any measurable effect. The correlation coefficient R^2 = 1 illustrates a highly linear correlated behavior. The standard error resulting from this PID-calibration equation was calculated under assumption of normal distribution.

Table S3: Theoretical values for toluene concentrations and values obtained experimentally using the PID for specific flows of toluene vapor u_{tol} (compensated with pure nitrogen to yield an overall flow of 500 mL/min). Experimental data match the theoretical within the margin of deviation.

u_{tol} [mL/min.]	Theoretical c_{tol} [ppm]	Experimental c_{tol} [ppm]
0	0.00	4 ± 2.8
100	43.2	47 ± 4.1
250	108	110 ± 8.5
400	173	172 ± 7.3
500	216	$2.1 \cdot 10^2 \pm 10.4$

Procedure S6 Calibration of the gas sensing set-up

This description complements the brief description of the gas sensing set-up in the Experimental procedures of the main paper. Because it takes a few minutes until the PID shows stable values, due to the large volume of the PID chamber, the toluene concentration cannot be measured directly during the experiments and an external calibration of the set-up is needed. The construction is shown in Fig. 8 of the main paper. For the calibration, the PID was placed at the outlet of the set-up and the switching valves were in a position allowing the flow to pass the sample chamber (temperature-regulated to $25\ ^\circ$C). As calibration points, we chose the flow ratios (u_{N_2} : u_{tol} in mL/min) 180:0, 160:20, 150:30, 140:40, 130:50 and 125:55. The total flow of 180 mL/min was chosen because this is the total flow during the experiments as well. After the signal became stable, values were taken every minute for three minutes in total. The whole procedure was repeated during three days.

[4] http://www.alphasense.com/WEB1213/wp-content/uploads/2017/05/AAN-305-06.pdf; accessed 01.02.2021.
[5] https://www.peaktech.de/produktdetails/kategorie/digital---handmultimeter/produkt/p-1035.html; accessed 01.02.2021.
[6] https://www.mksinst.com/f/ge50a-mass-flow-controller; accessed 01.02.2021.

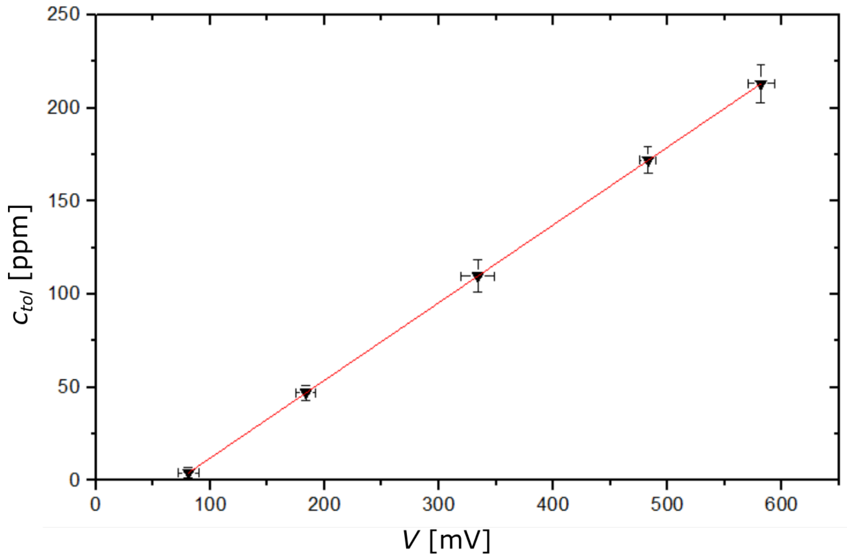

Figure S10: Calibration curve of toluene concentration (c_{tol}) to PID signal (V) with $c_{tol} = 0.41745 \cdot V - 29.61528$ and R^2 = 1. The calibration was repeated on three individual days.

Accuracies were determined considering errors resulting from the linear regression of the PID-calibration, standard deviations of the measurements and reading errors of the equipment (see previous section). Weighted linear regression gave the function $c_{tol} = 27.40048 \cdot u_{tol} + 0.49901$ and a correlation coefficient of R^2 = 0.9962 still expresses a significant linear correlation between toluene flow u_{tol} and concentration. Again, the first calibration point was set to zero for physical reasons.

Since the PID was operated at 3.6 V, the maximum reading is limited to this value, which corresponds to slightly more than 55 mL/min of toluene, at an overall flow of 180 mL/min (compensated with pure nitrogen). Unfortunately, our experiments required us to increase u_{tol} up to a practical maximum value of $u_{tol} = 140$ mL/min, thus exceeding the practical read-out scale of our PID. To verify that linearity still holds for $u_{tol} > 55$ mL/min (at a constant overall flow of 180 mL/min), three flow combinations with constant ratio were flown, differing only in overall flow. The combinations were 357:100, 429:120 and 500:140 and experiments were repeated on three different days. The obtained concentrations were compared to the calculated concentration resulting from the combination 141:39, that shows the same ratio but lies within the calibration range and has an overall flow of 180 mL/min, which allows to apply the received calibration function. The results are presented in table Table S4. Since all three data points match the theoretical value within the given margins of deviation, linearity is proven for flows up to 140 mL/min and concentrations corresponding to toluene flows higher than 55 mL/min can be extrapolated by the given function.

Table S4: Toluene concentrations measured for three flow combinations of same ratio but different overall flows and one combination of the same ratio, which is calculated using the obtained calibration function, since it is within the calibration range at an overall flow of 180 mL/min. The measured values match the calculated one within the margin of deviation and therefore linearity is proven for u_{tol} up to 140 mL/min.

Type	$u_{N_2} : u_{tol}$ [each flow in mL/min.]	c_{tol} [10^2 ppm]
Experimental	357:100	10.9 ± 0.83
Experimental	429:120	11.0 ± 0.75
Experimental	500:140	10.7 ± 0.23
Calculated	141:39	10.8 ± 0.38

Cell Reports
Physical Science

CellPress

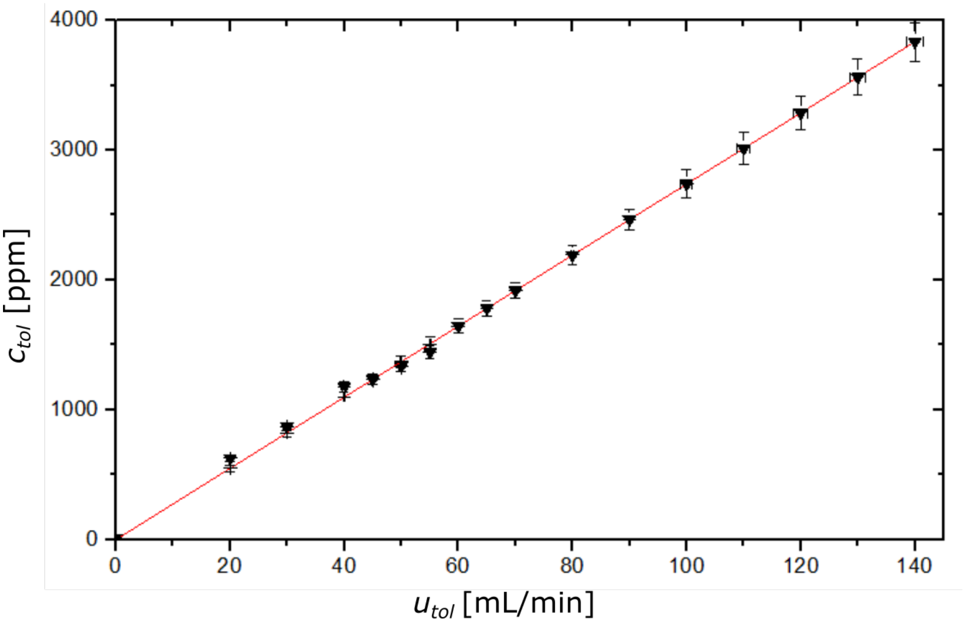

Figure S11: Calibration curve used to convert u_{tol} (at a constant overall flow of 180 mL/min, compensated with respective flows of nitrogen) to toluene concentration in ppm, following the function $c_{tol} = 27.40048 \cdot flow_{tol} + 0.49901$. Data points at $u_{tol} > 55$ mL/min are extrapolated, since linearity is proven for u_{tol} up to 140 mL/min (see table Table S4).The calibration was repeated on three individual days.

Figure S11 shows the final calibration curve used to convert u_{tol} (at a constant overall gas flow of 180 mL/min, compensated with respective flows of nitrogen) to toluene concentration in ppm. Data points at $u_{tol} > 55$ mL/min are extrapolated. Accuracies are determined under consideration of the errors resulting from linear regression and a factor for t-distribution (68 % confidence interval).

In this work we give values of c_{tol} in the commonly used unit ppm (parts per million). To avoid ambiguity concerning its interpretation, we point out that 1 ppm toluene means 1 mol toluene in 10^6 moles of all gases in the atmosphere. With our gas mixtures being solely toluene and nitrogen, 1 ppm would thus be defined as 1 mol of toluene and 999,999 moles of nitrogen. The ppm value can be converted[7] to mg/m^3 (referring to the mass of toluene in the volume of the gas mixture) by multiplication by 3.83 mg/m^3/ppm.

[7] https://gestis.dguv.de/data?name=010070; accessed 01.02.2021.
