# Supplementary material for: Quantitative volatile organic compound sensing with liquid crystal core fibers
Source: Cell Rep Phys Sci. 2021 Dec 22;2(12):100661. doi: 10.1016/j.xcrp.2021.100661 (PMC8724680; doi:10.1016/j.xcrp.2021.100661)
Supplement: Document S2. Article plus supplemental — information [file mmc12.pdf]

Cell Reports
Physical Science

CellPress
OPEN ACCESS

Article

Quantitative volatile organic compound sensing with liquid crystal core fibers

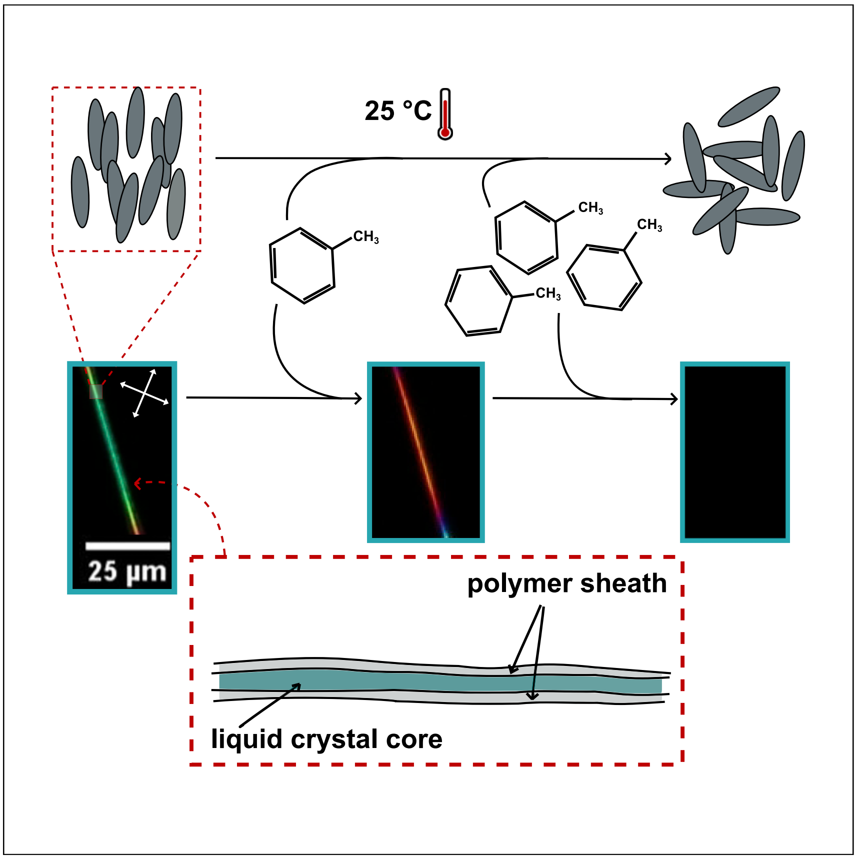

Katrin Schelski, Catherine G. Reyes, Lukas Pschyklenk, Peter-Michael Kaul, Jan P.F. Lagerwall

jan.lagerwall@lcsoftmatter.com

Highlights

Fully quantitative demonstration of VOC sensing with liquid crystal (LC) core fibers

Threshold VOC concentration depends sensitively on the clearing point of the LC

Response time is reduced from minutes to seconds by pre-exposure to VOC

Strategy for realizing competitive autonomous VOC sensors based on LC core fibers

Schelski et al. demonstrate that liquid crystal core-functionalized polymer fibers produced by electrospinning can sense volatile organic compounds (VOCs) rapidly, quantitatively, and fully autonomously, with highly repeatable performance. Their textile form factor is ideal for integrating in clothing or furniture, enabling new opportunities in ubiquitous VOC monitoring, at home and in public spaces.

Schelski et al., Cell Reports Physical Science *2*, 100661
December 22, 2021 © 2021 The Author(s).
https://doi.org/10.1016/j.xcrp.2021.100661

Cell Reports
Physical Science

CellPress
OPEN ACCESS

Article

Quantitative volatile organic compound sensing with liquid crystal core fibers

Katrin Schelski,[1,2,3,4] Catherine G. Reyes,[1,2,4,5] Lukas Pschyklenk,[2] Peter-Michael Kaul,[2] and Jan P.F. Lagerwall[1,6,*]

SUMMARY

Polymer fibers with liquid crystals (LCs) in the core have potential as autonomous sensors of airborne volatile organic compounds (VOCs), with a high surface-to-volume ratio enabling fast and sensitive response and an attractive non-woven textile form factor. We demonstrate their ability to continuously and quantitatively measure the concentration of toluene, cyclohexane, and isopropanol as representative VOCs, via the impact of each VOC on the LC birefringence. The response is fully reversible and repeatable over several cycles, the response time can be as low as seconds, and high sensitivity is achieved when the operating temperature is near the LC-isotropic transition temperature. We propose that a broad operating temperature range can be realized by combining fibers with different LC mixtures, yielding autonomous VOC sensors suitable for integration in apparel or in furniture that can compete with existing consumer-grade electronic VOC sensors in terms of sensitivity and response speed.

INTRODUCTION

The air that we breathe is a complex mixture of many chemicals, with the fraction composed by volatile organic compounds (VOCs) being of particular interest. The presence of certain VOCs in exhaled air can be a sign of severe health conditions,[1] and there is thus a strong interest in quantitative monitoring of VOCs in air for medical diagnostics.[2] Airborne VOCs can indicate imminent danger, being released by bacterial growth in food[3,4] or by explosive devices;[5] hence, their detection can alert people of spoiled meals or of attempted terrorist attacks. Moreover, they can have serious health effects upon inhalation, causing disease and/or allergenic reactions. The increasing awareness of these negative health effects has led to a surge in public interest in detecting VOCs, and there are now several electronic products for home usage on the market. Unfortunately, they rarely distinguish between different VOCs, as this is highly challenging for consumer-grade electronics-based sensors.[6–8] Better selectivity may be provided from specific chemical interactions between a target VOC and a dye or other responsive material, which reports the VOC presence through a change in appearance.[9–11] An interesting class of such optically VOC-responsive materials is given by liquid crystals (LCs),[12,13] which despite their liquid nature exhibit long-range orientational order along a direction called the "director."[14] This gives them properties normally seen only in crystalline solids, such as birefringence and, in case of chiral LCs, structural color.[15] LCs have been shown to signal exposure to toluene,[16–20] acetone,[19,21] NO_2,[22] CO_2,[23,24] O_2,[24] amines,[25] cyclohexane and acetic acid,[26] chloroform and ethanol,[27,28] isopropanol,[29] tetrahydrofuran, methanol, tetrachloroethylene,[27] pyridine, hexane, and benzene[21] and to

[1]Department of Physics and Materials Science, University of Luxembourg, 162a Avenue de la Faiencerie, 1511 Luxembourg, Luxembourg

[2]Institute of Safety and Security Research, University of Applied Sciences Bonn-Rhein-Sieg, von-Liebig-Straße 20, 53359 Rheinbach, Germany

[3]Lawrence Livermore National Laboratory, Livermore, CA, USA

[4]These authors contributed equally

[5]Present address: Lawrence Livermore National Laboratory, Materials Engineering Directorate, 7000 East Avenue, Livermore, CA 94550, USA

[6]Lead contact

*Correspondence:
jan.lagerwall@lcsoftmatter.com

https://doi.org/10.1016/j.xcrp.2021.100661

This is an open access article under the CC BY license (http://creativecommons.org/licenses/by/4.0/).

VOCs mimicking the nerve gas sarin.[30–33] Apart from making selectivity to specific VOCs easier than for typical consumer-grade electronic sensors, the non-electronic response of LCs, being fundamentally a VOC-induced change of optical characteristics driven by thermal energy alone, renders them ideal for autonomous sensors that require no power source.

A problem for LC-based sensors is, however, to find a convenient form factor, as the liquid nature renders an unprotected LC sensitive to mechanical impact, and an open flat LC sample has only one surface exposed to the VOC, giving a low surface-to-volume ratio, which increases the response threshold concentration and response time. An interesting approach to address these challenges is to encapsulate the LC into the core of thin fibers with a polymeric sheath produced by electrospinning.[14,16] This ensures a high surface-to-volume ratio with access to VOC on all sides, as well as a convenient containment, mechanically protecting the LC while still allowing VOCs to permeate through the thin polymer sheath, and giving access to the optical properties of the LC. A further attractive aspect is the textile form factor, ideal for incorporation in apparel as wearable devices or within home/office furniture.

In brief (Figure 1A), electrospinning[34] relies on the charging of a drop of polymer solution or melt (we use the former) by a strong electric field until the attraction to a grounded or oppositely charged collector is so strong that it overcomes the surface tension. A thin jet ejects and moves rapidly, under strong simultaneous stretching, toward the collector, eventually forming the dry fiber if the polymer molar mass is high enough. If not, the Rayleigh instability breaks the jet into droplets (electrospray) before solidification. The LC is often incorporated as a distinct core already at the spinneret level[35] using a coaxial spinning geometry,[36] but it may also be dissolved in the polymer solution, relying on phase separation during solvent evaporation for the appearance of the coaxial geometry.[37] We refer readers interested in the details pertaining to LC-functionalized fiber spinning to the now quite rich body of prior research (see, e.g.,[19,35,37–47]).

Much of the work so far focused on fabricating the fibers reproducibly, as the many steps of the procedure entail challenges from a general materials science and physical chemistry point of view. These range from the complex phase behavior when the LC comes into contact,[48] or is co-dissolved,[19,39] with polymer solutions, followed by the strongly non-equilibrium situation as solvent evaporates under the sometimes counterintuitive impact of humidity in the air, affecting the process from the emergence of the Taylor cone at the spinneret[46] to the wetting of the collected fibers on the substrates.[43] Even the effects on the LC self-assembly by the strong cylindrical confinement are far from trivial.[35,38,40–42] This focus has left the demonstrations of the gas sensing functionality largely on a qualitative level, with only two reports showing semiquantitative (arbitrary units) data.[19,20]

Moreover, almost all experiments have been done with the same single-component LC, 4-cyano-4-pentylbiphenyl (5CB),[16,18–20] exhibiting a nematic LC phase at room temperature, with a nematic-isotropic transition (clearing) at T_{NI} = 35.6°C. Since these studies relied on triggering the nematic-isotropic transition by the VOC exposure, the role of T_{NI} clearly plays an important role, yet its impact has not been investigated. One study used cholesteric LC mixtures with higher clearing points,[23] but in that case the response was in terms of a change in the pitch of the cholesteric helix, without triggering clearing. In this study, we spin coaxial fibers with core consisting of 5CB or the 5CB-based nematic mixture E7 (T_{NI} ≈ 58°C), and we follow a path

CellPress
OPEN ACCESS

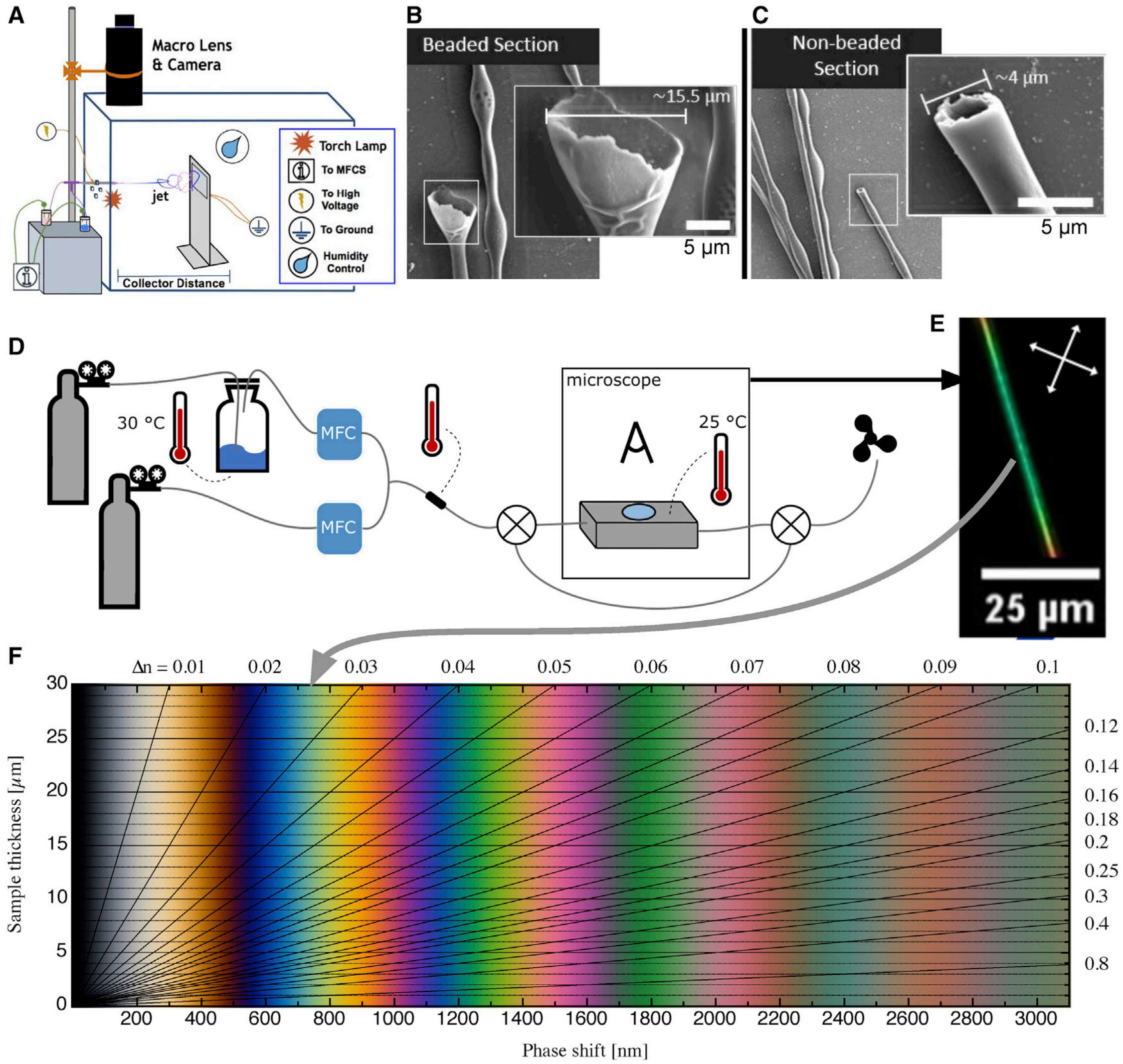

Figure 1. Concept graphic for quantitative VOC sensing using LC-functionalized electrospun fibers

The key components of our horizontal electrospinning setup are shown in (A); MFCS is a pneumatic flow control unit. Two examples of fractured fibers imaged by scanning electron microscopy (SEM) show cross sections of beads (B) and non-beaded fiber segments (C), respectively. The hollow interior was filled with LC prior to evacuation for SEM imaging. During VOC sensing tests (D), the fibers are kept in a gas-tight cell with glass windows, placed on a polarizing optical microscope (POM), and connected to a mixed flow of inert carrier gas and VOC at controlled concentration. The LC birefringence (Δn) gives the fiber a characteristic color in POM (E), the identification of which in the Michel-Lévy diagram (F) allows us to establish Δn. Since Δn reflects the LC orientational order S, this allows us to quantify the VOC impact on S, which is the basis for measuring the VOC concentration.

toward quantitative analysis that we initiated earlier.[20] We carefully and continuously monitor the magnitude of birefringence (Δn) of the fiber-encapsulated LC in response to varying VOC concentration. While we confirm the response to toluene, isopropanol, as well as cyclohexane, we focus primarily on toluene, correlating Δn to the concentration of toluene vapor (c_{tol}) as measured by a calibrated laboratory-grade electronic toluene sensor.

RESULTS

Polarizing microscopy on unexposed electrospun fibers

Before exposing our fibers to VOCs, we investigate them in the pristine state in polarizing optical microscopy (POM). The morphology of LC-filled fibers often varies

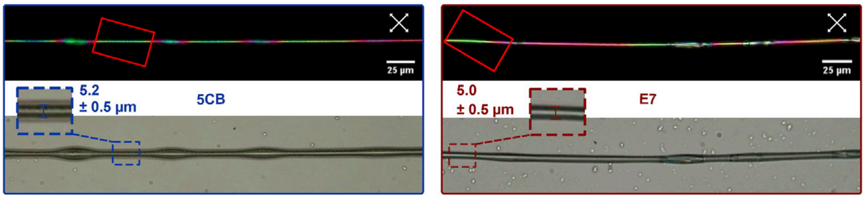

Figure 2. Pristine electrospun PVP sheath fibers filled with 5CB and E7, respectively
Between crossed polarizers (upper row; white double arrows indicate polarizer orientations), interference colors due to LC birefringence can be observed. The red rectangles mark the regions monitored during toluene exposure in Figures 3 and 4. The lower row shows the same fiber sections without analyzer, allowing the distinction between beaded and non-beaded parts. These images are also used to measure the diameters (see insets; accuracy limited to ± 0.5 µm by resolution of POM). The outermost dark-gray region surrounding the 5CB-filled fibers is not the fiber edge but a thin coating on the substrate. Most likely, this coating formed as condensation water spread on the glass substrate,[43] bringing some dissolved PVP with it. Scale bars, 25 µm (valid for images with and without analyzer).

somewhat, e.g., with "beads" of locally increased diameter (Figure 1B) appearing with semi-regular intervals along a fiber that is otherwise cylindrical (Figure 1C). We see this with E7 as well as with 5CB core (see Figure 2 for representative examples). While non-beaded sections have an average diameter of ~4 µm, beads vary in size, from the most common marginal expansion to more extreme cases where the local bead diameter surpasses 10 µm.

When viewed at 25°C between crossed polarizers, the LC-filled fibers show intense interference colors reflecting the relatively high Δn of the encapsulated LC. This is in strong contrast to homogeneous fibers of pure polyvinylpyrrolidone (PVP), which appear dark in POM, since the type of PVP used in our study does not crystallize (see Figure S1). Occasionally, weak birefringence of pure PVP fibers can be observed and arises due to the strong stretching of the jet in-flight, aligning the polymer chains to some extent along the final dry fiber.[49] However, this alignment may not occur in the LC-filled fibers: the strain experienced by the drying polymer solution is very different in the coaxial geometry, around a non-volatile core, and the tensile strain along the jet is shared between core and sheath. With coaxial fibers we may thus expect low Δn for the sheath, which, together with the fact that the imaged PVP is much thinner than in pure PVP fibers (because the same volume of PVP is distributed around the LC core), leads to negligible impact from the sheath in POM. Indeed, as will be clear from later figures, a 5CB- or E7-filled fiber imaged in POM appears black if the core goes isotropic. Any birefringence remaining in the polymer sheath can thus safely be ignored in comparison to that of the LC core. This is important for the validity of the quantitative measurements described below.

The director, and thus the optic axis, of nematic LCs incorporated within electrospun fibers generally orients along the fiber. This can be verified by investigating the fibers in POM, orienting them along and perpendicular, respectively, to the optic axis of an inserted first-order λ plate (Note S1). Upon rotating the fibers in the POM without the λ plate we can also confirm to what extent the director orientation is uniform within the fibers: in Figures S3E and S3F we find that the straight segments between beads appear perfectly dark every time the fiber is oriented along one of the crossed polarizers, while the beads never go dark, regardless of fiber orientation. They only shift their interference color as the sample is rotated. This indicates that the director orientation is uniform in the segments between beads, while in beads, particularly large ones, we have a (partially) twisted director

CellPress
OPEN ACCESS

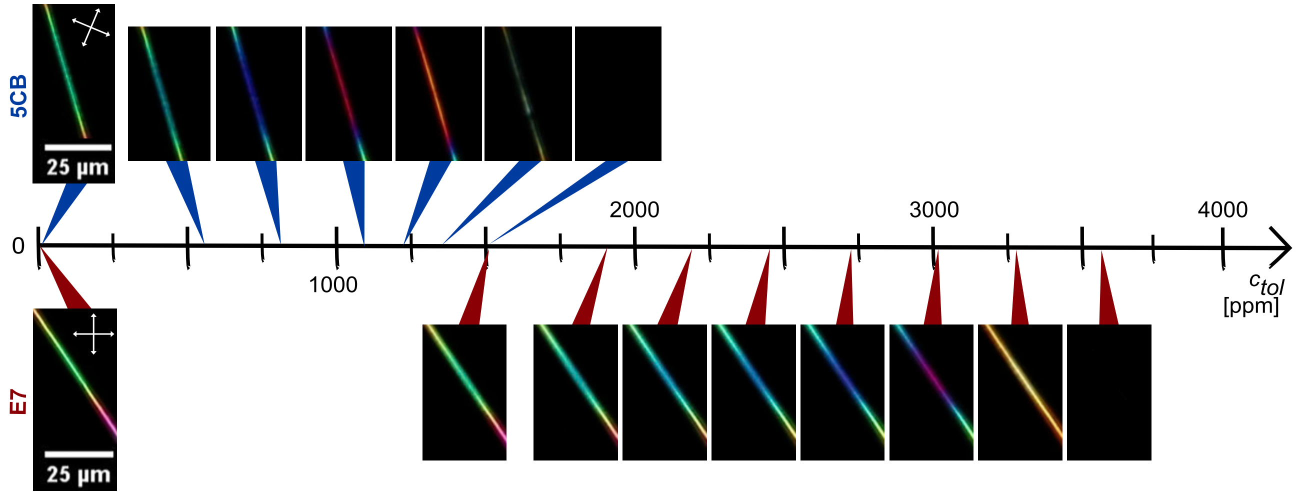

Figure 3. Optical response to toluene exposure

Representative non-beaded segments of two electrospun fibers (within red rectangles in Figure 2) filled with 5CB (top) and E7 (bottom), respectively, responding to their first exposure to toluene vapor, at successively increasing concentration c_{tol} (without purging with pure nitrogen in between), at a temperature of 25°C. The diameters, including the PVP sheath, are 5.2 ± 0.5 μm (5CB core) and 5.0 ± 0.5 μm (E7 core). Each image shows the appearance between crossed polarizers (white double-headed arrows) at the end of a 1-min exposure at constant c_{tol}. The white scale bar applies to all images, extracted from video recordings of the experiments (Video S1).

field, where the optic axis at the top of the fiber is not parallel to that at the bottom, hence precluding extinction. Its optics is significantly more challenging to analyze quantitatively than that of a uniform director field. For this reason, the gas sensing measurements presented below do not show data from the beaded sections of the fibers, as they cannot be directly compared with the classic Michel-Lévy chart describing the interference color of birefringent samples with uniform optic axis directions.

The untwisted regions between beads are, alternatively, very well suited for such a comparison, forming the basis for our quantitative analysis. We thus limit this analysis to the fiber segments in red rectangles in Figure 2, showing the exact same segments that are analyzed in more detail in Figures 3 and 4. With matching diameters between the two fiber types, those segments are well suited to compare the behavior of the two LCs exposed to toluene vapor (Figure 1D). Furthermore, because Δn at room temperature is only slightly greater in E7 than in 5CB, the fact that the segments appear with similar color confirms that the two selected sample sections have a similar cross section not only in the sample plane but also perpendicular to it, along the viewing direction. As will become clear below, the green color of these segments in their unexposed state corresponds to a second-order green in the Michel-Lévy chart (see gray arrow in Figures 1E and 1F), thus an optical retardation of $r \approx 700$ nm.

The response to controlled toluene vapor exposure

In contrast to the previous reports of VOC response of LC-functionalized fibers, which mainly studied entire fiber mats showing varying scattering,[16] brightness between crossed polarizers,[18,19] or color changes,[23] in this study we use a tailor-made gas exposure cell mounted on a polarizing optical microscope, inspired by Hunter et al.[33] and proposed in 2018,[20] to investigate the response at the level of a single LC filled fiber exposed to a specified concentration of VOC (see Figure 1 and Supplemental experimental procedures for details).

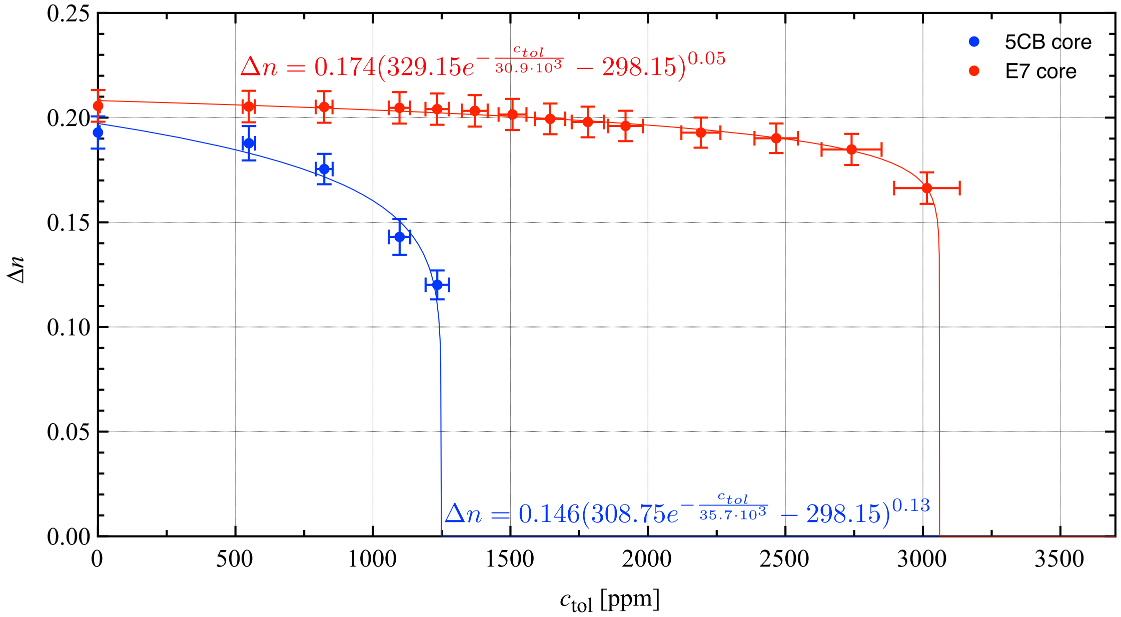

Figure 4. Quantitative analysis of birefringence as a function of toluene exposure

Birefringence Δn of segments of 5CB- (blue) and E7-filled (red) fibers (same as in Figures 2 and 3) as a function of toluene concentration c_{tol} in the atmosphere (first exposure), at a temperature of 25°C. The blue and red curves are best fits of Equation 2 to the experimental data. Each data point is an average of all Δn values during the last 15 s of 1 min of exposure at constant c_{tol}. Each individual Δn value is calculated following Equation 1, assuming d to be 70% of the outer fiber diameter. Error bars (see Supplemental experimental procedures) for the concentrations arise from the calibration of the gas sensing setup, described and shown in Supplemental experimental procedures

When fibers filled with either 5CB or E7 are exposed to toluene vapor, a gradual change in interference color in POM proceeds under increasing c_{tol} (see Figure 3) The progression of color shift is leftward in the Michel-Lévy chart (see Figure 1F), thus toward reduced Δn. The color changes from the original second-order green to second-order blue, then entering the first order with purple, red, orange, and yellow, before the fiber suddenly turns black, indicating a complete loss of birefringence, i.e., the core has undergone the first-order phase transition from nematic to isotropic. Importantly, this transition is fully reversible, and the LC regains its birefringence once the toluene is removed. Note that the color change is perfectly smooth and continuous throughout the process up to the nematic-isotropic transition, with the images shown being representative snapshots from videos tracking the experiment (see Video S1 for the case of the E7-filled fiber).

The experiments are successfully repeated about 30 times with fibers produced from several electrospinning processes. In very rare cases, fibers that are apparently malformed are encountered, recognized by the fact that the original texture does not recover after complete clearing and following removal of the analyte (see Note S2). The birefringence returns but with a highly non-uniform texture. Since this phenomenon is rare (studying on the order of 100 fibers, we see this irreversible behavior only in 3), we did not investigate it further. We hypothesize that the sheath may not be fully intact in these fibers, allowing LC flow outside the core during the phase transition, changing the LC configuration irreversibly.

Interestingly, while the sequence of color change is qualitatively identical for 5CB- and E7-filled fibers, we see in Figure 3 that it is shifted to significantly higher c_{tol} with E7 in the core. For the fiber filled with 5CB (upper row), the first clearly

detectable color shift (from the first to the second photo) can be seen at $c_{tol} \approx 0.6 \times 10^3$ ppm, whereas for the one filled with E7 (bottom row), the corresponding threshold is more than 1,000 ppm higher, at $c_{tol} \approx 1.9 \times 10^3$ ppm. In fact, at $c_{tol} = 1.50 \times 10^3$ ppm, where the 5CB-filled fiber turns black, indicating the loss of nematic order and $\Delta n = 0$, the E7-filled fiber still shows no visible change in POM appearance. For the E7-filled fiber to lose its birefringence, an exposure to $c_{tol} = 3.6 \times 10^3$ ppm is required. We also observe that the c_{tol} range from the first detectable color change to the loss of nematic order is almost twice as large for E7 ($c_{tol} \approx 1.7 \times 10^3$ ppm) compared to 5CB ($c_{tol} \approx 0.9 \times 10^3$ ppm).

To make the analysis of the response quantitative, we turn the detected interference colors into numerical values for Δn at each value of c_{tol}. The full procedure is described in detail in Supplemental experimental procedures. The red, green, and blue values (R, G, B) in the digital image file are first converted into hue values (H) of the HSL (hue, saturation, lightness) color space. Translation into the optical retardation (r) by comparison with a Michel-Lévy chart with reliable color representation[50] follows. Since hue values cannot express different shades of gray, the analysis is only performed for retardation values greater than $r = 300$ nm. Assuming that any variation of the LC core diameter (d) due to toluene exposure in the experiment can be neglected, we can then calculate the birefringence Δn (dimensionless) as:

$$\Delta n = \frac{r}{1000d}, \quad (1)$$

where we for practical convenience consider the values of r in nm and d in μm.

It remains to establish d, which is not simply equal to the outer fiber diameter as seen in POM. Based on scanning electron microscopy (SEM) investigations of fractured LC-filled fibers of the same type as used in this study, Reyes concluded as a rough estimate that the LC core in non-beaded electrospun fibers with PVP sheath and 5CB core comprises 60%–80% of the total fiber diameter.[49] We thus estimate that d for both the 5CB and E7 fibers equals ~70% of the outer fiber diameter measured by optical microscopy in the non-beaded sections. We confirm that this is a reasonable assumption by comparing our Δn produced by Equation 1 for unexposed fibers with values reported in the literature. For the unexposed 5CB- and E7-filled fibers at a temperature of $T = 25°$C we obtain $\Delta n_{5CB} = 0.193 \pm 0.008$ and $\Delta n_{E7} = 0.206 \pm 0.008$, fitting well with earlier reports of $\Delta n^{litt}_{5CB} = 0.197$ and $\Delta n^{litt}_{E7} = 0.227$ at $T = 20°$C.[51] While the match for 5CB is excellent, our slightly lower value for E7 suggests that we may have overestimated d slightly for this fiber, yet the discrepancy is fully acceptable. When calculating error bars for the data shown below, any error introduced by the estimation of d is not considered, since it is a systematic error affecting all absolute values to the same extent, thus not the relative changes as c_{tol} is increased.

We are now in a position to provide a fully quantitative depiction of the toluene sensing response. The plot in Figure 4 shows Δn of the 5CB- and E7-filled fibers, respectively, as a function of the c_{tol} values to which they are exposed. Since the fibers need some time to reach complete response (see the next section for an analysis of the temporal behavior), i.e., to reach the equilibrium state under a new level of toluene exposure, the Δn value at each plotted data point is obtained as an average of all Δn values measured during the last 15 s out of the full 60 s of exposure (see Supplemental information for details).

The LC orientational order parameter S—and thus Δn—is theoretically predicted[52] to follow a power law dependence on T of the type $S = a + b(T_{NI} - T)^{\beta}$. Mean-field

behavior would predict β = 0.5, but experiments indicate that β ≈ 0.25 better describes the real temperature dependence.[52] Hypothesizing that the primary function of toluene exposure is to depress the clearing point T_{NI}, we therefore fit a function,

$$\Delta n = a + b\left(T_{NI}^{0} e^{-\frac{c_{tol}}{\sigma}} - T\right)^{\beta}, \quad (2)$$

to our experimental data, where we let T_{NI}^{0} be the clearing point in the absence of toluene exposure and we assume, as a first simple model, an exponential decrease of T_{NI} with increasing c_{tol}, with a characteristic concentration σ. Fitted to the toluene response data, this function reproduces the data very well (Figure 4), albeit with even lower values of β, on the order of β = 0.1. We fix T_{NI}^{0} = 35.6°C for 5CB while for E7, being a mixture, we release T_{NI}^{0} as a fitting parameter around 58°C, obtaining a value T_{NI}^{0} = 56°C from the fitting. The measuring temperature is fixed at T = 25°C. The characteristic concentration σ, representing the sensitivity of the LC to toluene exposure, is similar but not identical for 5CB and E7.

While we focus our work on non-beaded fiber segments, to enable the quantitative analysis, we also make qualitative observations of the response of beads, in particular concerning the loss of birefringence at T_{NI}. We find that the critical c_{tol} value to clear the LC is similar in and between beads, at least for bead diameters no larger than twice that of the unbeaded segment. For lower c_{tol}, during which the LC is in the nematic state, the bead response is complicated by the twist and, most likely, topological defects related to reversal of twist handedness.[53–55] A detailed investigation of this response will be a topic for future studies.

As discussed later, the underlying principle of the fiber response is not specific to toluene but may occur in comparable manner in the presence of other VOCs. A few simple experiments with vapors of isopropanol (Note S3) and cyclohexane (Note S4), respectively, on 5CB-filled fibers qualitatively show that both gases initiate a color change following the Michel-Lévy chart, as toluene does. However, isopropanol dissolves the PVP sheath, leading to leaking out of the LC filling.

Temporal aspects of the response and test of repeatability with an individual fiber

As mentioned in the previous section, the vanished LC birefringence reappears when removing toluene from the atmosphere, something we accomplish by flushing the sample cell with pure nitrogen. To test whether the response to toluene exposure is quantitatively reproducible, two individual fibers filled with 5CB and E7, respectively, are exposed to increasing c_{tol} during three consecutive experiments, with nitrogen flushing in between (see Supplemental experimental procedures for details). A fiber filled with E7, shown in Figure 5 during three consecutive runs at c_{tol} = 0 and at c_{tol} = 3.0 × 10^3 ppm, respectively, here serves as an example. The experiment is repeated twice for each LC type, yielding similar results regarding repeatability.

A first comparison of the POM pictures does not show any major differences between the responses of the individual runs: $\Delta n \neq 0$ reappears after nitrogen flushing, and the fiber in the absence of toluene always shows the same color as before the first exposure. However, looking at Δn plotted over time t (measured in the segment marked with a red rectangle in the images from the first exposure), the first run clearly differs from the following ones. While there is a continuous, smooth decrease in Δn

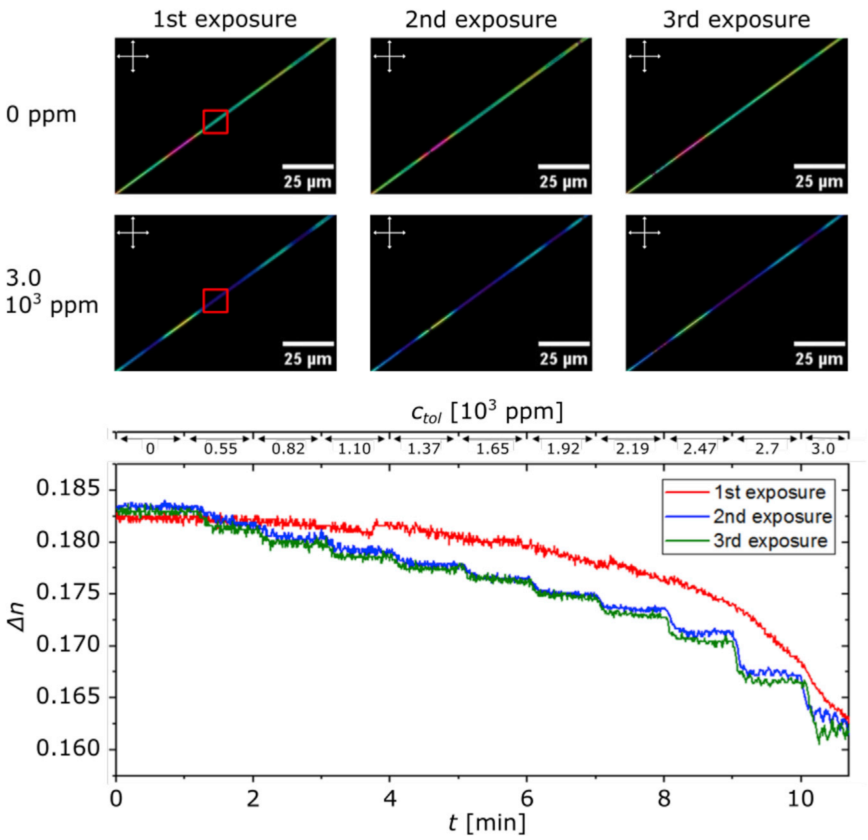

Figure 5. Response during repeated VOC exposures

(Top) An E7-filled fiber exposed to increasing c_{tol} (accuracy, ~5%) during three consecutive experiments, viewed between crossed polarizers. After each run, the sample chamber is flushed with pure nitrogen for 1 min, followed by ~1.5 h of pause before the next run is initiated. Images are extracted from videos of each entire experiment and show, for each run, the fiber appearance prior to exposure or during pure nitrogen flow, and at $c_{tol} = 3.0 \times 10^3$ ppm, respectively. (Bottom) Δn of the segment marked with a red rectangle in the images above, plotted against time t while every minute c_{tol} is increased in discrete steps (the values are indicated along the top). In the segment, the outer fiber diameter is 5.3 ± 0.5 μm.

throughout the entire first run (the small jump after 3.7 min is most likely an artifact from the conversion from R, G, B color representation into Δn) despite the fact that c_{tol} is increased in discrete steps, the decrease in Δn mimics the steps in c_{tol} during the second and third runs.

Table 1 expresses this phenomenon in numbers and allows comparison between the runs. We define a response time (t_r) as the time needed after a step change in c_{tol} for the fiber to go from 10% to 90% of its saturated equilibrium response at the new c_{tol} level. With this definition we cannot determine t_r for the first run, because it is much greater than the 60-s step length over which c_{tol} is kept constant, i.e., the response does not reach 90% of saturation before c_{tol} is changed. In contrast, t_r is well within the step window during runs 2 and 3, and it remains nearly unchanged between these runs. To find their respective 10% and 90% levels, we assume that the last 15 s of the full minute of exposure to constant c_{tol} shows the equilibrium state, thus defining the level corresponding to 100% response. Additionally, a single experiment on a 5CB-filled fiber exposed to toluene vapor suggests an even higher repeatability of at least 10-fold after pre-exposure (see Note S5).

Table 1. Response time as function of repeated exposure

Δc_{tol} [10^3 ppm]	t_r^1 [s]	t_r^2 [s]	t_r^3 [s]
0.82 → 1.10	>60	14 ± 2	12 ± 2
1.65 → 1.92	>60	19 ± 3	17 ± 3
2.47 → 2.7	>60	12 ± 2	10 ± 2

Response times t_r^i of the E7-filled fiber shown in Figure 5 for three representative step changes in c_{tol} and three consecutive repeated exposures, numbered i = 1, 2, 3. Concentration values are given with accuracies of ~5%.

DISCUSSION

By mapping the variation in Δn as a function of c_{tol}, we not only turn the LC-functionalized fibers into true continuous sensors, capable of quantifying the concentration of a VOC to which they are exposed, but we also demonstrate that the range in c_{tol} over which the fibers respond depends greatly on the clearing point, T_{NI}, of the LC used. Since E7 to 50% consists of 5CB, and since the other three components (7CB, 8OCB, and 5CT) are chemically very similar, the difference in response behavior is unlikely to reflect different specific molecular interactions between LC and analyte. Likewise, the fact that both fiber types are spun following identical procedures under identical conditions, using the same polymer, and as both fiber samples appear with very similar morphology during POM investigations, we consider any impact of the fiber sheath and overall fiber morphology on the response differences to be negligible. We can also rule out that temperature variations during exposure would have impacted our observations, as our sensing experiments are performed under constant gas flow with the fibers contained in a specially designed cell with temperature control, maintaining the fibers at T = 25°C.

The higher T_{NI} of E7 compared to 5CB is often said to mean a greater stability of the nematic order, and it is this that gives rise to the different response range: toluene dissolving into the LC acts as an impurity that disturbs the long-range orientational ordering of the nematic phase, required for birefringence. The more impurity is added, the more T_{NI} is depressed compared to the pure LC. Since T_{NI} of 5CB is near room temperature already for the undisturbed LC, the clearing point is brought close enough to room temperature already at the low (compared to E7) toluene exposure level of $c_{tol} \approx 0.6 \times 10^3$ ppm, and with $c_{tol} = 1.50 \times 10^3$ ppm, T_{NI} is reduced to 25°C, thus clearing the core material at the temperature of our experiments. Even if the relative impact of toluene exposure is comparable with E7, the starting point there is a more robustly ordered nematic, requiring much greater c_{tol} to bring down the clearing point far enough from the original $T_{NI} \approx 58$°C to observe an effect at a temperature of 25°C.

Future extensions need to carry out the quantitative gas exposure experiments as a function of temperature, in order to produce a richer set of data that allows reliable fitting of β and σ in Equation 2, and testing whether an exponential decay of T_{NI} with increasing c_{tol} is appropriate or if another function better describes the response. However, the excellent fits in Figure 4 strengthen the hypothesis that the effect of the toluene is primarily to suppress the clearing point. An interesting conclusion from these observations is that an LC core with lower T_{NI} than that of 5CB would give fibers that respond to lower VOC exposure levels, reaching truly competitive sensor performance. The regulatory permissible exposure limit (PEL) for toluene given by the Occupational Safety and Health Administration (OSHA) of the US for an 8-h time-weighted average (TWA) is 200 ppm, with 300 ppm as an acceptable ceiling concentration.[56] Recommended limits are half as high. PELs released by

the European Commission (Commission Directive 2006/15/EC[57]) are even lower, giving 50 ppm for TWA and 100 ppm for short time exposure. Setting $a = 0$, $b = 0.15$, $\beta = 0.1$, and $\sigma = 3.5 \times 10^3$ ppm in Equation 2, in line with our fitting results, we can estimate that the response threshold of LC-functionalized fiber sensors would meet such criteria at $T = 25°C$ if an LC mixture with $T_{NI} \approx 28°C$ is used.

The problem with this strategy of working with T_{NI} near ambient temperature is that the temperature window of operation becomes very narrow: on a warm day the LC may have cleared even without any exposure. To exploit this opportunity one would thus need to combine multiple LCs in a single sensor. A useful strategy would be to combine patches of mats spun with different LCs in the fiber cores, the different T_{NI}s of which cover the full operation temperature range with relatively small intervals. Since LC mixtures can be used (as demonstrated by the successful use of E7 as core), such tuning of T_{NI} is easily achieved, with commercially available and quite low-cost materials. In this way, we could ensure that one subset mat is always at operation conditions that ensure high sensitivity regardless of temperature. Since the nematic-isotropic transition is fully reversible, the compound sensor could be continuously in use as the temperature varies throughout the day, throughout the year, or in zones of operation with varying climate.

A garment-integrated sensor of this type would typically be extended along one dimension, with a temperature axis along it that guides the user to the right patch to observe. Even better, if the nematic core LCs are exchanged for suitably adapted cholesteric mixtures, the temperature responsiveness of the cholesteric reflection color[40] could dynamically provide this kind of guidance, while simultaneously rendering the use of POM for readout unnecessary. We will come back to the latter aspect below.

The strong reduction in response time t_r after the first toluene exposure run in Figure 5 shows that the exposure to toluene introduces structural or compositional modifications to the fiber sheath that are irreversible on the timescale of the full experiment. Interestingly, these modifications are highly beneficial, because they are apparently completed after the first exposure, ensuring repeatable behavior during subsequent experiments, and they do not negatively impact the response. Quite on the contrary, they lead to a dramatically faster response, reducing t_r from minutes to seconds. What could these sheath changes be and why do they have such beneficial impact? Toluene is a poor solvent for PVP, unable to dissolve the polymer used in our study ($\overline{M_w} \approx 10^6$g/mol). However, the interactions are still favorable enough for PVP to form a gel when immersed in liquid toluene (see Figure S2). In our experiments, we can thus assume that toluene condensing on the fiber sheath swells the PVP. As the exposure level increases, more and more toluene is incorporated, until the sheath approaches a state of a compact gel. Above a certain threshold we may expect channels of toluene that connect the outside atmosphere with the LC core.

While purging the fibers with pure nitrogen, toluene fully leaves the LC core, within experimental accuracy, as suggested by the fact that we recover the pristine optical properties. It is more difficult to know whether also the PVP sheath is free of toluene, as complete drying of solvent-swelled polymers is a slow process. However, here we have a very thin layer of PVP with a high surface-to-volume ratio, exposed to a rather poor solvent, and hence we do think that most of the toluene leaves also the sheath. If the removal of toluene is sufficiently fast, the polymer may have no chance to relax from its swelled state, leaving nanoscopic pores throughout the sheath, which would

enable much faster access of toluene vapor to the LC core during subsequent exposures. As a reference, extraction of the 10% by mass gelled PVP in Figure S2, and drying at room temperature overnight in a desiccator left a stiff, porous PVP film. If a similar porous structure would prevail in the sheath of the toluene-exposed fibers, it would explain the much reduced t_r. Future experiments should test this by high-resolution electron microscopy on fibers before and after exposure.

Another explanation may be in a change in sheath composition rather than structure. In our previous work,[23] we found that E7-filled PVP fibers liquefy completely at $T \approx 73^{\circ}C$, suggesting a much reduced glass transition temperature T_g compared to that of pure PVP fibers, which do not lose their shape even at 200°C. This means that E7 can enter the sheath, functioning as a plasticizer and softening the PVP considerably. By swelling the sheath with toluene during the first gas sensing experiment, additional LC is likely to mix into the sheath, remaining there even after the toluene has evaporated. This then softens the sheath further and also increases the solubility of toluene during subsequent exposures, which would be another way of explaining the reduced t_r. A differential scanning calorimetry (DSC) analysis of pristine mats and mats exposed once to toluene may be a good way of testing this hypothesis: if the first toluene exposure leaves the PVP mixed with more LC than in the pristine fibers, T_g should be reduced.

Long-term studies are needed to test whether the dramatic reduction in t_r is transient, which may be the case if toluene remains in the PVP sheath between runs, or permanent, which would be expected if the sheath is free of toluene but rendered either porous via rapid solvent evaporation, or plasticized with additional LC. Support of the change being permanent, with no toluene remaining, is given by the fact that the original fiber appearance was recovered immediately after 1 min of nitrogen purging, with identical appearance 1.5 h later, when the next run was started.

Moreover, we have confirmed long-term impact on the PVP sheath of toluene exposure on a microscopic scale, by studying the morphology of discontinuously filled fibers with strongly beaded character by POM before and after repeated toluene exposure (see Figure 6). We show the exact same fiber regions without (A and B) and with analyzer (C and D), clearly revealing irreversible shape changes in the beads in the form of depressions that are large enough to detect optically. Comparing C and D, we note that the region between the beads has more uniform LC filling after the toluene exposure. These two effects suggest that the PVP sheath was softened during toluene exposure, as expected during solvent swelling, allowing capillary forces from the LC to suck LC from the beaded regions to cavities in the cylindrical ones, giving rise to the depressions in the beads and filling the fibers more uniformly. The effect on the sheath of the initial exposure to toluene suggests that fibers intended for use in gas sensing should always be pre-exposed to any vapors with which they may come into contact and which may swell the polymer.

A simple way of bringing down the clearing point of a nematic LC is to add a chiral dopant, which forms an isotropic liquid on its own at room temperature, such as the commonly used 5CB-related molecule CB15. The chiral dopant has the additional benefit of turning the nematic into a chiral nematic, or cholesteric, phase, inducing a helical modulation of the orientational order. With enough chiral dopant, the pitch of the helix can be short enough to give rise to visible Bragg reflection, rendering the mixture strongly colored. By mixing a chiral dopant such as CB15 with a high-T_{NI} mixture such as E7, one can easily achieve a cholesteric mixture that reflects visible colors across a wide temperature range, and also tune T_{NI} accordingly. This will thus

CellPress
OPEN ACCESS

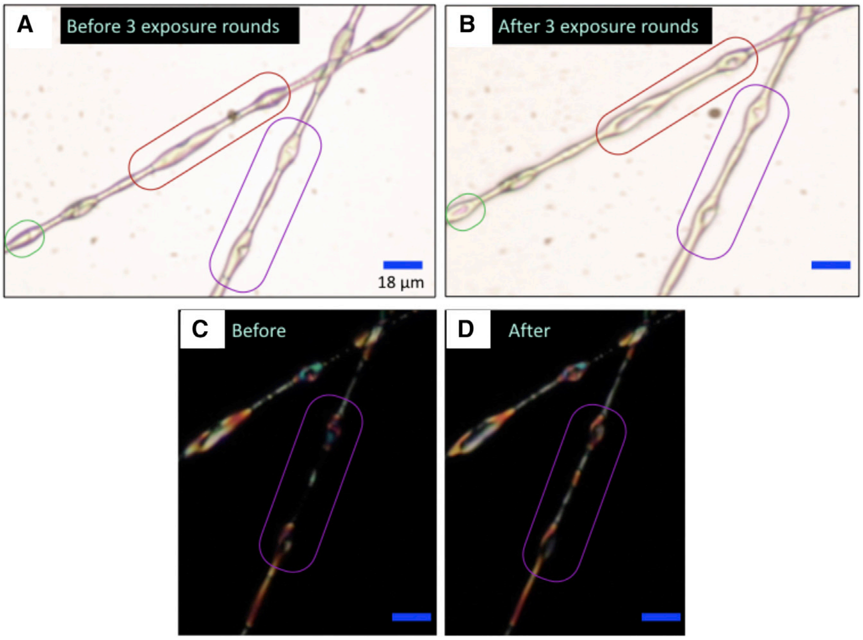

Figure 6. Irreversible changes to PVP sheath upon toluene exposure
Two beaded 5CB-filled fibers with discontinuous cores before (A) and immediately after (B) three rounds of toluene exposure. The red and purple outlined regions in (B) show areas where the fiber sheath deflates after toluene exposure. Additionally, LC moves from beads to non-beaded regions, as seen with the fibers between crossed polarizers in (C) and (D).

be an interesting path to explore, on the one hand bringing down T_{NI} enough to get highly sensitive response to VOC exposure, and on the other hand switching from a response amounting to a change in Δn, which normally requires a POM for analysis, to a change in reflection color, which could be detected by the naked eye. The ideal situation is one where the helix pitch varies from deep red to deep violet in the temperature range just below T_{NI}, because that might provide a sensor mat segment that both indicates when the temperature is appropriate for it to be used as a VOC sensor, and a VOC response that is easily readable to the naked eye.

We see two further alternatives to meeting the crucial requirement of removing the dependence on a POM for the analysis. First, one can use the classic "guest-host" principle[58] of colored LCDs (common, e.g., in railway stations) in which a dichroic dye is mixed into a nematic LC host. The appropriate dye has a mesogen-like shape, such that it is aligned along the LC director, maximizing its absorption and thus the color effect when light passes through the fiber. If an analyte causes a transition to the isotropic phase, the alignment control is lost, and the color intensity should decrease, giving a response that can be visible to the naked eye. With a smart combination of dichroic dyes,[58] the effect may be enhanced to give strong contrast. The second possibility might be to make a layered composite that encompasses the key elements of the POM setup that we are using here, but in a wearable format. While this is clearly more challenging, linear polarizers have been made by electrospinning,[59] and hence the LC-functionalized fibers could be placed between two such sheets of polarizing fibers to create the crossed-polarizer geometry, while still ensuring good contact with the air in order that the VOC can enter the fibers. The lamp of the POM would most naturally be replaced by ambient light, and the detector and colorimetric analysis could be conveniently carried out by using organic

photovoltaic cells on the inside of the composite structure: Kang et al.[60] demonstrated a self-powered electronic sensor of NO_2 by sandwiching a film that changes its transparency in response to NO_2 exposure over an organic photovoltaic cell, tuned for operation in the wavelength range of maximum response of the film. The same principle might be used to turn the variations in Δn of our LC core into an electrical signal, fueled by ambient light.

The next critical issue is to add selectivity to the response. Since the response we are dealing with here is simply related to the action of a VOC as impurity in the LC, reducing the degree of orientational order, any solute with this effect (e.g., acetone, chloroform, ethanol) will have a similar impact, as demonstrated qualitatively on the examples of isopropanol (Note S3) and cyclohexane (Note S4). To bring in selectivity, we thus need to mix in an additive that reacts specifically to a certain analyte in such a way that its interaction with the LC changes, causing the phase to change in a way that can easily be detected in the presence of this analyte. A powerful choice is the already mentioned chiral dopant that turns a nematic host phase into a cholesteric exhibiting selective reflection, if a chiral dopant is identified that changes its helical twisting power (HTP) when exposed to the target analyte. Several teams have demonstrated this principle, using such cholesteric mixtures to selectively detect CO_2, NO_2, O_2, and H_2O.[22–24,61] On its own, this may not bring the desired high sensitivity, since the analyte-induced change in HTP is a bulk effect, and even for samples with high surface-to-volume ratio such as fibers, the required VOC concentration may be high. However, as the HTP is dependent not only on the dopant but also on the host, not least on the elastic constants, which are strongly related to its orientational order, it may be possible to combine this approach with a mixture that has low clearing point, in order to get high sensitivity and selective response simultaneously.

One of the most successful approaches to achieve selective and sensitive VOC response with LCs is to use a VOC-responsive aligning layer.[12,32,33] If this layer changes its influence on the LC, imposing tangential rather than normal alignment or vice versa, upon exposure to a certain analyte, this can give a strong optical response at very low VOC concentration, since only the bounding surface needs to be saturated, not the bulk LC. This approach may be transferred to LC-filled fibers if the specific aligning agent (for instance perchlorate salts[32,33]) can be made to aggregate at the interface between the polymer sheath and LC. Alternatively, a simpler approach may be to make use of the VOC-induced change in the order parameter studied in this paper in combination with an alignment layer that changes its aligning impact as the LC order parameter reduces upon approaching the clearing transition. We recently demonstrated such an effect using spherical shells of nematic 5CB and E7, stabilized against aqueous phases using the amphiphilic block copolymer Pluronic F-127.[62] While the alignment is tangential deep in the nematic phase, it turns normal as the nematic-isotropic transition is approached. Spinning LC-filled fibers with a block copolymer such as F-127 incorporated in the sheath, or adsorbing at the LC-sheath interface, may be easier than incorporating inorganic salts, as the F-127 can be co-dissolved with the sheath polymer. With a color-reflecting cholesteric LC core, the change from tangential to normal alignment is easily recognized by eye, since only tangential alignment provides the geometry that yields color reflection. Also, with modifications of the amphiphilic block copolymer structure that incorporate analyte-responsive moieties, a specific response might be achieved.

Finally, a critical requirement is to replace the PVP in the sheath of the fibers studied here with a better suited polymer. PVP is highly humidity sensitive and mechanically

delicate, precluding the use of PVP sheath fibers in garments. The reason that it is frequently used in LC-core fiber spinning is that it is easy to spin fibers with ethanol-dissolved PVP around LC cores such as 5CB, while other polymers, dissolved in other solvents, are much more challenging to spin with good LC filling. In a parallel research thrust we are currently exploring a method to spin LC-filled fibers with a polymer sheath that can be crosslinked to achieve stability. Using carefully selected solvents and polymers we obtained very promising results, which will be reported separately. Once fibers can be spun that have a sufficient filling of LC yet use a polymer sheath that is robust enough to incorporate into garments, it remains to be seen whether the pre-exposure to the gaseous analyte that had such a beneficial effect in increasing response speed can still be used. Overall, the impact of variations of the air quality apart from VOCs, in addition to humidity also including issues such as suspended particulates, needs to be investigated in future studies. While there are thus many challenges that remain to be addressed, we hope this study also shows that there are many promising routes to achieve wearable VOC sensors based on LC-functionalized fibers that may find practical use.

EXPERIMENTAL PROCEDURES

Resource availability

Lead contact

Further information and requests for resources and reagents should be directed to and will be fulfilled by the lead contact, Jan P. F. Lagerwall (jan.lagerwall@lcsoftmatter.com).

Materials availability

This study did not generate new unique materials.

Data and code availability

The authors declare that data supporting the findings of this study are available within the article and in the Supplemental information. All other data are available from the Lead contact upon reasonable request. The only code produced was the MATLAB script for extracting RGB color pixel values from the videos of the gas sensing experiments; this script is included as Data S9.

Materials

To produce a suitable homogeneous polymer solution for the fiber sheath, 12.5 wt% PVP ($\overline{M_w} = 1.3 \times 10^6$g/mol; Sigma-Aldrich) was dissolved in ethanol ($\geq$99.8%; Merck) by stirring overnight at room temperature. The nematic LC 5CB was obtained from Yantai Xianhua Chem-Tech, and the nematic LC mixture E7 was obtained from Synthon Chemicals. Gas bottles of premixed toluene test gas (216 mol ppm in nitrogen) and of pure nitrogen, respectively, were purchased from Messer. Additionally, liquid toluene ($\geq$99.5%; Merck), cyclohexane (Merck), and isopropanol (Merck) were used.

Electrospinning setup and parameters for fiber production

Figure 7 is a schematic representation of the electrospinning setup, with a microfluidic pumping system and an insulating chamber of polymethylmethacrylate (PMMA), containing the coaxial spinneret (see Supplemental experimental procedures) and collector. The latter had two ring-shaped copper wires connected to ground, fixed to a PMMA holder, allowing the collection of free-hanging fibers. The metallic outer needle of the spinneret was connected to the positive pole of the high-voltage power supply (Gamma High Voltage ES30P-5 W/DAM) to apply voltages between 7.75 and 8.5 kV. The distance between the needle tip and collector was 10 cm. To flow the

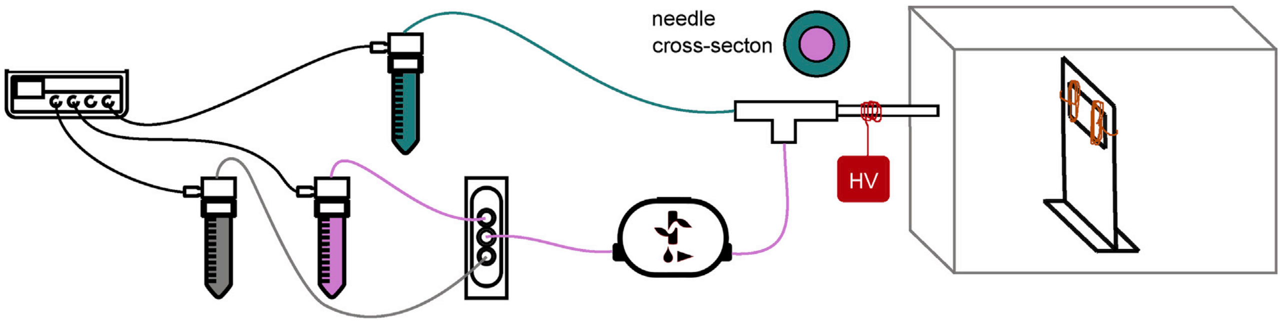

Figure 7. Electrospinning setup

Schematic sketch of electrospinning setup with coaxial needle for production of LC-filled PVP fibers. A microfluidics pressure control unit is used to pump PVP solution (turquoise), LC (pink), and/or ethanol (gray, for cleaning) through the system. High voltage (HV) is applied to the spinneret's needle and allows fiber formation on a grounded collector, placed in an insulating chamber. Images from Fluigent are used with permission.[63]

LC (5CB and E7, respectively) and PVP solution, specially designed reservoirs (Fluigent P-CAP and Fluiwell-4C) were pressurized using a pressure control unit (Fluigent MFCS-EZ, maximum pressure 1,034 mbar) to 700–750 mbar for LC and 700 mbar for PVP solution. The resulting flow rate u of the LC was measured using a microfluidic flow control unit (Fluigent Flow Unit M), yielding 18–38 μL/min. For the polymer solution, the flow rate was determined to ~18 μL/min by an external calibration (see Supplemental experimental procedures for details). Furthermore, a two-way bidirectional valve (Fluigent, 2-SWITCH) allowed easy, half-automated cleaning of the setup after use. Temperature (22.5°C–23.5°C) and relative humidity (31%–40%) were monitored by a thermo-hygrometer (TFA Dostmann 30.5002), and a digital camera (Zarbeco Z505-OR2) with a macro lens monitoring the Taylor cone. The whole setup was placed in a fume hood.

Gas sensing setup and experimental procedure

The gas sensing test setup is shown in Figure 8. The fiber sample was placed on a glass slide in a custom-built, gas-tight holding cell of aluminum (see Supplemental experimental procedures), with windows on top and bottom allowing optical monitoring of the response to VOC exposure at varying concentrations. The cell was placed between crossed polarizers of a POM (Nikon LV100, operating in transmission mode) with a digital camera (Canon EOS 706) attached for recording. A working temperature of 25°C was assured using an electronic temperature sensor and two heating resistors fixed to the cell.

Different concentrations of toluene vapor were realized by controlled dilution of a flow of nitrogen carrier gas with known toluene concentration with pure nitrogen. To this end, mass flow controllers (MFCs; MKS, models GM50A013502MAV020 and GE50A013502MAV020) were used to adjust both individual flows, always maintaining a constant overall flow. In order to reach higher concentrations than that of the pre-mixed toluene test gas, a gas washing bottle filled with liquid toluene was placed in between the test gas bottle and the corresponding MFC. The gas washing bottle was placed in a water bath at 30°C for constant evaporation conditions. Before the mixed gas stream entered the sample cell, the temperature was measured and a valve allowed to decide if the gas should pass through the sample or be led into a bypass ending in the fume hood. A photo ionization detector (PID; AlphaSense PID-A1, 10.6 eV lamp) functioned as concentration control during the calibration of the system (see Supplemental experimental procedures for more details), in combination with a power supply giving 3.6 V DC and a multimeter

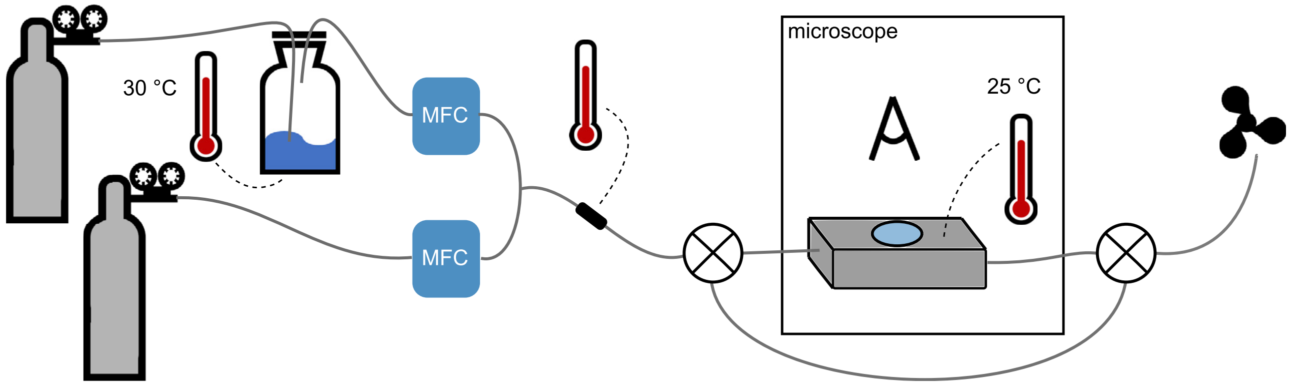

Figure 8. Quantitative VOC sensing setup

Sketch of the setup used to monitor the fiber response to different concentrations of VOC. Concentrations are adjusted by dilution of a stream of VOC vapor with pure nitrogen. Mass flow controllers (MFCs) allow to set the flow of the two streams, and a temperature-controlled gas washing bottle filled with liquid VOC is used to yield higher concentrations. The fiber sample is placed in a temperature-controlled holding cell and can be observed with a POM using transmitted light. The temperature of the gas mix can be measured before entering the cell, and a bypass with two switching valves allows leading the stream directly to the fume hood without passing the sample if needed.

(PeakTech 1035) for reading the PID response. For the experiments with isopropanol and cyclohexane, respectively, the test gas bottle was replaced by a second nitrogen gas bottle and the gas washing bottle was filled with the liquid analyte.

The POM appearance of fibers before and during VOC exposure was recorded to video with consistent camera settings for all experiments. After checking for director twists by rotation of the sample, VOC was led through the sample chamber. Starting with 0 mL/min of the toluene-containing gas mixture, its flow was increased in steps of 10 mL/min or 5 mL/min while the flow of pure nitrogen gas was decreased to yield a constant overall gas flow of 180 mL/min. Each ratio was maintained for 1 min. Once the exposure level for LC clearing had been reached, the holding cell was purged with pure nitrogen. The experiments were repeated multiple times with fibers produced from several electrospinning experiments. To check for quantitative repeatability of the response of an individual fiber, the whole procedure was repeated three times. This was done with two fibers for each LC. Gas flows were converted into concentrations using the calibration already mentioned, with the results shown in Supplemental experimental procedures. The experimental videos were analyzed according to Supplemental experimental procedures.

SUPPLEMENTAL INFORMATION

Supplemental information can be found online at https://doi.org/10.1016/j.xcrp.2021.100661.

ACKNOWLEDGMENTS

All experiments were undertaken at the University of Luxembourg. We thank V. Reis-Adonis for his assistance in advising us on the design and construction of the gas sensing cell, and U. Siegel and N. Tournier for assistance in adapting components for the gas sensing setup. The contribution of C.G.R. was made possible by Lawrence Livermore National Laboratory (LLNL), operated by Lawrence Livermore National Security, LLC, for the U.S. Department of Energy, National Nuclear Security Administration under contract DE-AC52-07NA27344. This article's release no. is LLNL-JRNL-819466. Financial support from the European Research Council under the European Union's Seventh Framework Programme (FP/2007-2013)/ERC (CoG

project INTERACT, grant agreement no. 648763) and from the Federal Ministry of Education and Research of Germany (project OptoSpin [FKZ 13FH023IX6]) is gratefully acknowledged.

AUTHOR CONTRIBUTIONS

Conceptualization, J.P.F.L., C.G.R., and K.S.; methodology, all authors; software, K.S.; validation, K.S. and C.G.R.; formal analysis, J.P.F.L., C.G.R., K.S., and L.P.; investigation, K.S. and C.G.R.; data curation, K.S. and C.G.R.; writing – original draft, K.S., C.G.R., and J.P.F.L.; writing – review and editing, K.S. and J.P.F.L.; visualization, K.S. and C.G.R.; supervision, P.-M.K., L.P., C.G.R., and J.P.F.L. All authors have read and agreed to the published version of the manuscript.

DECLARATION OF INTERESTS

J.P.F.L. is a member of the Advisory Board of Cell Reports Physical Science.

Received: May 6, 2021
Revised: October 1, 2021
Accepted: November 3, 2021
Published: November 22, 2021

REFERENCES

1. Nakhleh, M., Amal, H., Jeries, R., Broza, Y., Aboud, M., Gharra, A., Ivgi, H., Khatib, S., Badarneh, S., Har-Shai, L., et al. (2017). Diagnosis and classification of 17 diseases from 1404 subjects via pattern analysis of exhaled molecules. ACS Nano *11*, 112–125.

2. Konvalina, G., and Haick, H. (2014). Sensors for breath testing: From nanomaterials to comprehensive disease detection. Acc. Chem. Res. *47*, 66–76.

3. Klein, D., Maurer, S., Herbert, U., Kreyenschmidt, J., and Kaul, P. (2018). Detection of volatile organic compounds arising from chicken breast filets under modified atmosphere packaging using TD-GC/MS. Food Anal. Methods *11*, 88–98.

4. Xu, Y., Cheung, W., Winder, C.L., and Goodacre, R. (2010). VOC-based metabolic profiling for food spoilage detection with the application to detecting *Salmonella typhimurium*-contaminated pork. Anal. Bioanal. Chem. *397*, 2439–2449.

5. Ostmark, H., Wallin, S., and Ang, H.G. (2012). Vapor pressure of explosives: A critical review. Propellants Explos. Pyrotech. *37*, 12–23.

6. Spinelle, L., Gerboles, M., Kok, G., Persijn, S., and Sauerwald, T. (2017). Review of portable and low-cost sensors for the ambient air monitoring of benzene and other volatile organic compounds. Sensors (Basel) *17*, E1520.

7. Szulczyński, B., and Gębicki, J. (2017). Currently commercially available chemical sensors employed for detection of volatile organic compounds in outdoor and indoor air. Environments *4*, 21.

8. Kohl, D. (2001). Function and applications of gas sensors. J. Phys. D Appl. Phys. *34*, R125–R149.

9. Azzouz, A., Vikrant, K., Kim, K.H., Ballesteros, E., Rhadfi, T., and Malik, A.K. (2019). Advances in colorimetric and optical sensing for gaseous volatile organic compounds. Trends Analyt. Chem. *118*, 502–516.

10. Owyeung, R.E., Panzer, M.J., and Sonkusale, S.R. (2019). Colorimetric gas sensing washable threads for smart textiles. Sci. Rep. *9*, 5607.

11. Wang, G., Cai, Z., and Dou, X. (2021). Colorimetric logic design for rapid and precise discrimination of nitrate-based improvised explosives. Cell Rep. Phys. Sci. *2*, 100317.

12. Carlton, R.J., Hunter, J.T., Miller, D.S., Abbasi, R., Mushenheim, P.C., Tan, L.N., and Abbott, N.L. (2013). Chemical and biological sensing using liquid crystals. Liq. Cryst. Rev. *1*, 29–51.

13. Nayani, K., Yang, Y., Yu, H., Jani, P., Mavrikakis, M., and Abbott, N. (2020). Areas of opportunity related to design of chemical and biological sensors based on liquid crystals. Liq. Cryst. Today *29*, 24–35.

14. Urbanski, M., Reyes, C.G., Noh, J., Sharma, A., Geng, Y., Subba Rao Jampani, V., and Lagerwall, J.P. (2017). Liquid crystals in micron-scale droplets, shells and fibers. J. Phys. Condens. Matter *29*, 133003.

15. Schütz, C., Bruckner, J.R., Honorato-Rios, C., Tosheva, Z., Anyfantakis, M., and Lagerwall, J.P.F. (2020). From equilibrium liquid crystal formation and kinetic arrest to photonic bandgap films using suspensions of cellulose nanocrystals. Crystals (Basel) *10*, 199.

16. Kim, D.K., Hwang, M., and Lagerwall, J.P.F. (2013). Liquid crystal functionalization of electrospun polymer fibers. J. Polym. Sci. B Polym. Phys. *51*, 855–867.

17. Bedolla Pantoja, M.A., and Abbott, N.L. (2016). Surface-controlled orientational transitions in elastically strained films of liquid crystal that are triggered by vapors of toluene. ACS Appl. Mater. Interfaces *8*, 13114–13122.

18. Reyes, C.G., Sharma, A., and Lagerwall, J.P. (2016). Non-electronic gas sensors from electrospun mats of liquid crystal core fibers for detecting volatile organic compounds at room temperature. Liq. Cryst. *43*, 1986–2001.

19. Wang, J., Jákli, A., and West, J.L. (2018). Liquid crystal/polymer fiber mats as sensitive chemical sensors. J. Mol. Liq. *267*, 490–495.

20. Reyes, C.G., and Lagerwall, J.P. (2018). Advancing flexible volatile compound sensors using liquid crystals encapsulated in polymer fibers. Proc. SPIE 10555, Emerging Liquid Crystal Technologies XIII, 105550O (February 8, 2018). https://doi.org/10.1117/12.2292533.

21. Winterbottom, D.A., Narayanaswamy, R., and Raimundo, I.M. (2003). Cholesteric liquid crystals for detection of organic vapours. Sens. Actuators B Chem. *90*, 52–57.

22. Spengler, M., Pschyklenk, L., Niemeyer, J., Kaul, P., and Giese, M. (2021). Photonic NO_2 gas sensing with binaphthyl-based dopants. Adv. Opt. Mater. *9*, 2001828.

23. Pschyklenk, L., Wagner, T., Lorenz, A., and Kaul, P. (2020). Optical gas sensing with encapsulated chiral-nematic liquid crystals. ACS Appl. Polym. Mater. *2*, 1925–1932.

24. Han, Y., Pacheco, K., Bastiaansen, C.W.M., Broer, D.J., and Sijbesma, R.P. (2010). Optical monitoring of gases with cholesteric liquid crystals. J. Am. Chem. Soc. *132*, 2961–2967.

25. Sutarlie, L., Qin, H., and Yang, K.L. (2010). Polymer stabilized cholesteric liquid crystal arrays for detecting vaporous amines. Analyst (Lond.) *135*, 1691–1696.

26. Shibaev, P., Carrozzi, D., Vigilia, L., and DeWeese, H. (2019). Liquid crystal nose, chiral case: Towards increased selectivity and low detection limits. Liq. Cryst. *46*, 1309–1317.

27. Mujahid, A., Stathopulos, H., Lieberzeit, P.A., and Dickert, F.L. (2010). Solvent vapour detection with cholesteric liquid crystals—Optical and mass-sensitive evaluation of the sensor mechanism. Sensors (Basel) *10*, 4887–4897.

28. Hussain, A., Semeano, A.T.S., Palma, S.I.C.J., Pina, A.S., Almeida, J., Medrado, B.F., Pádua, A.C.C.S., Carvalho, A.L., Dionísio, M., Li, R.W.C., et al. (2017). Tunable gas sensing gels by cooperative assembly. Adv. Funct. Mater. *27*, 1700803.

29. Kim, I., Kim, W.S., Kim, K., Ansari, M.A., Mehmood, M.Q., Badloe, T., Kim, Y., Gwak, J., Lee, H., Kim, Y.K., and Rho, J. (2021). Holographic metasurface gas sensors for instantaneous visual alarms. Sci. Adv. *7*, eabe9943.

30. Sridharamurthy, S., Cadwell, K., Abbott, N., and Jiang, H. (2008). A microstructure for the detection of vapor-phase analytes based on orientational transitions of liquid crystals. Smart Mater. Struct. *17*, 012001.

31. Bungabong, M.L., Ong, P.B., and Yang, K.L. (2010). Using copper perchlorate doped liquid crystals for the detection of organophosphonate vapor. Sens. Actuators B Chem. *148*, 420–426.

32. Hunter Jacob, T., and Abbott Nicholas, L. (2013). Dynamics of the chemo-optical response of supported films of nematic liquid crystals. Sens. Actuators B Chem. *183*, 71–80.

33. Hunter, J.T., Pal, S.K., and Abbott, N.L. (2010). Adsorbate-induced ordering transitions of nematic liquid crystals on surfaces decorated with aluminum perchlorate salts. ACS Appl. Mater. Interfaces *2*, 1857–1865.

34. Reneker, D., and Yarin, A. (2008). Electrospinning jets and polymer nanofibers. Polymer (Guildf.) *49*, 2387–2425.

35. Lagerwall, J.P.F., McCann, J.T., Formo, E., Scalia, G., and Xia, Y. (2008). Coaxial electrospinning of microfibres with liquid crystal in the core. Chem. Commun. (Camb.) *42*, 5420–5422.

36. Yarin, A. (2011). Coaxial electrospinning and emulsion electrospinning of core-shell fibers. Polym. Adv. Technol. *22*, 310–317.

37. Wang, J., Jákli, A., and West, J.L. (2016). Morphology tuning of electrospun liquid crystal/polymer fibers. ChemPhysChem *17*, 3080–3085.

38. Enz, E., Baumeister, U., and Lagerwall, J. (2009). Coaxial electrospinning of liquid crystal-containing poly(vinylpyrrolidone) microfibres. Beilstein J. Org. Chem. *5*, 58.

39. Buyuktanir, E.A., Frey, M.W., and West, J.L. (2010). Self-assembled, optically responsive nematic liquid crystal/polymer core-shell fibers: Formation and characterization. Polymer (Guildf.) *51*, 4823–4830.

40. Enz, E., and Lagerwall, J. (2010). Electrospun microfibres with temperature sensitive iridescence from encapsulated cholesteric liquid crystal. J. Mater. Chem. *20*, 6866–6872.

41. Scalia, G., Enz, E., Calò, O., Kim, D.K., Hwang, M., Lee, J.H., and Lagerwall, J.P.F. (2013). Morphology and core continuity of liquid-crystal-functionalized, coaxially electrospun fiber mats tuned via the polymer sheath solution. Macromol. Mater. Eng. *298*, 583–589.

42. Enz, E., La Ferrara, V., and Scalia, G. (2013). Confinement-sensitive optical response of cholesteric liquid crystals in electrospun fibers. ACS Nano *7*, 6627–6635.

43. Kim, D.K., and Lagerwall, J.P.F. (2014). Influence of wetting on morphology and core content in electrospun core-sheath fibers. ACS Appl. Mater. Interfaces *6*, 16441–16447.

44. Lin, J.D., Chen, C.P., Chen, L.J., Chuang, Y.C., Huang, S.Y., and Lee, C.R. (2016). Morphological appearances and photo-controllable coloration of dye-doped cholesteric liquid crystal/polymer coaxial microfibers fabricated by coaxial electrospinning technique. Opt. Express *24*, 3112–3126.

45. Bertocchi, M.J., Vang, P., Balow, R.B., Wynne, J.H., and Lundin, J.G. (2019). Enhanced mechanical damping in electrospun polymer fibers with liquid cores: Applications to sound damping. ACS Appl. Polym. Mater. *1*, 2068–2076.

46. Reyes, C.G., and Lagerwall, J.P.F. (2020). Disruption of electrospinning due to water condensation into the Taylor cone. ACS Appl. Mater. Interfaces *12*, 26566–26576.

47. Thum, M.D., Ratchford, D.C., Casalini, R., Wynne, J.H., and Lundin, J.G. (2021). Azobenzene-doped liquid crystals in electrospun nanofibrous mats for photochemical phase control. ACS Appl. Nano Mater. *4*, 297–304.

48. Reyes, C.G., Baller, J., Araki, T., and Lagerwall, J.P.F. (2019). Isotropic-isotropic phase separation and spinodal decomposition in liquid crystal-solvent mixtures. Soft Matter *15*, 6044–6054.

49. Reyes, C.G. (2019). Confined in a fiber: Realizing flexible gas sensors by electrospinning liquid crystals. PhD thesis (University of Luxembourg).

50. Michel-Lévy interference color chart issued by Zeiss Microscopy. https://en.wikipedia.org/wiki/Interference_colour_chart.

51. Khoo, I. (2007). Liquid Crystals: Physical Properties and Nonlinear Optical Phenomena (Wiley).

52. de Gennes, P.G., and Prost, J. (1993). The Physics of Liquid Crystals (Clarendon Press).

53. Dietrich, C.F., Rudquist, P., Lorenz, K., and Giesselmann, F. (2017). Chiral structures from achiral micellar lyotropic liquid crystals under capillary confinement. Langmuir *33*, 5852–5862.

54. Nayani, K., Chang, R., Fu, J., Ellis, P.W., Fernandez-Nieves, A., Park, J.O., and Srinivasarao, M. (2015). Spontaneous emergence of chirality in achiral lyotropic chromonic liquid crystals confined to cylinders. Nat. Commun. *6*, 8067.

55. Jeong, J., Kang, L., Davidson, Z.S., Collings, P.J., Lubensky, T.C., and Yodh, A.G. (2015). Chiral structures from achiral liquid crystals in cylindrical capillaries. Proc. Natl. Acad. Sci. USA *112*, E1837–E1844.

56. United States Department of Labor; Occupational Safety and Health Administration (2021). Permissible exposure limits – Annotated tables. https://www.osha.gov/annotated-pels/table-z-2.

57. The Commission of the European Communities (2006). Commission directive 2006/15/EC. https://eur-lex.europa.eu/legal-content/EN/TXT/PDF/?uri=CELEX:32006L0015.

58. Ivashchenko, A.V. (1994). Dichroic Dyes for Liquid Crystal Displays (CRC Press).

59. Yao, Y., Gu, Z., Zhang, J., Pan, C., Zhang, Y., and Wei, H. (2007). Fiber-oriented liquid crystal polarizers based on anisotropic electrospinning. Adv. Mater. *19*, 3707.

60. Kang, K., Park, J., Kim, B., Na, K., Cho, I., Rho, J., Yang, D., Lee, J.Y., and Park, I. (2020). Self-powered gas sensor based on a photovoltaic cell and a colorimetric film with hierarchical micro/nanostructures. ACS Appl. Mater. Interfaces *12*, 39024–39032.

61. Saha, A., Tanaka, Y., Han, Y., Bastiaansen, C.M.W., Broer, D.J., and Sijbesma, R.P. (2012). Irreversible visual sensing of humidity using a cholesteric liquid crystal. Chem. Commun. *48*, 4579–4581.

62. Noh, J., Wang, Y., Liang, H.L., Jampani, V.S.R., Majumdar, A., and Lagerwall, J.P.F. (2020). Dynamic tuning of the director field in liquid crystal shells using block copolymers. Phys. Rev. Res. *2*, 033160.

63. Fluigent (2021). Image package for scientific use. https://www.fluigent.com/de/download/.

Cell Reports Physical Science, Volume *2*

Supplemental information

Quantitative volatile organic compound sensing with liquid crystal core fibers

Katrin Schelski, Catherine G. Reyes, Lukas Pschyklenk, Peter-Michael Kaul, and Jan P.F. Lagerwall

Cell Reports
Physical Science

CellPress

Supplemental Information

Supplemental Figures

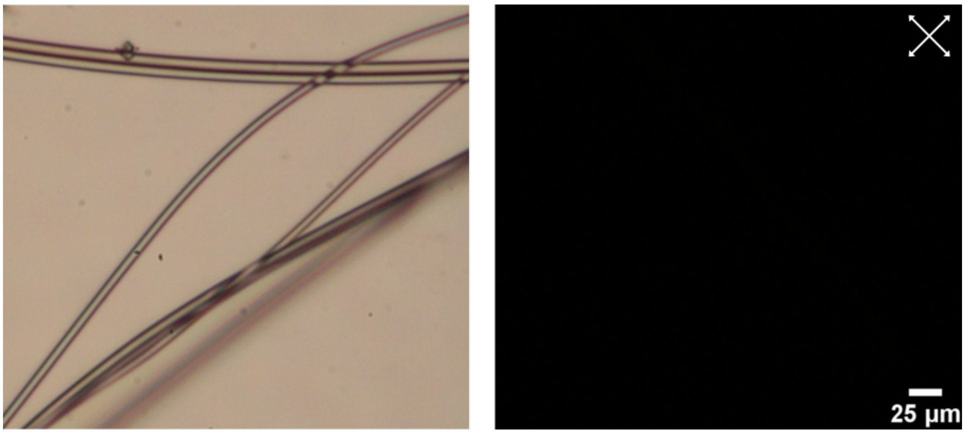

Figure S1: Electrospun fibers of pure PVP, without any core material, imaged using POM, without (left) and with (right) analyzer, related to the statement in the first Results section in the main paper, concluding that the birefringence seen in POM can be attributed entirely to the LC core. In contrast to liquid crystal filled fibers, no birefringence is detected, rendering the photo obtained with crossed polarizers uniformly black. The white scale bar of 25 μm is valid for both pictures.

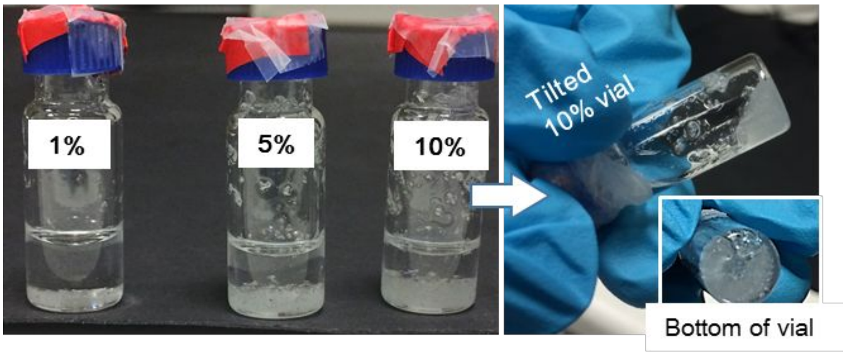

Figure S2: Demonstration of toluene being a poor solvent for PVP, relating to the discussion in the main paper on the impact of the first exposure of the fibers to toluene vapor. Different mass fractions of PVP (molar mass: 10^6 g/mol) in toluene showing that, while PVP is insoluble in toluene, it does gel. In tilting the vial containing the 10 % mass fraction of PVP we see how the gel separates and sticks to the bottom of the container.

Cell Reports
Physical Science

CellPress

Supplemental notes

Note S1 POM investigation of the ground state director orientation within the fibers

This note relates to the statements in the first Results section in the main paper, regarding the orientation of the LC director in beaded and unbeaded sections of a fiber. As demonstrated in Figure S3, the LC-core fibers generally have the director uniformly aligned along the fiber axis, although there can be local variations. In panels A–D, two nearly parallel fibers are viewed between crossed polarizers in a POM while they are oriented approximately $0°$, $45°$, $90°$ and $135°$ to the polarizer, respectively. In every situation, the fibers are photographed once with a first-order λ-plate inserted (left half, pink background) and once without this plate (right half, black background). These fibers were not used in the gas sensing experiments described in this paper, but they were spun under similar conditions with a 5CB core inside a PVP sheath, giving diameters similar to those used in the gas sensing study, hence they are comparable in many respects.

The interference color between crossed polarizers, without λ-plate (right parts of B and D), alternates between a second-order yellow and a first order orange (in the Michel-Lévy chart we may locate the colors at retardations of $r \approx 800$ nm and $r \approx 470$ nm, respectively) between the thinnest straight cylindrical segments and the beads in the fibers. It is interesting that the beads in these fibers show *lower*, not higher, retardation, indicating *less* LC filling than in the sections between beads. We speculate that this is due to the beads being formed via a Rayleigh instability acting on the PVP solution but not on the LC core while spinning these fibers. The beads would then have accumulated PVP, leaving a thinner LC core than in the cylindrical segments between.

The identification with certainty of the retardation values in the thin and thick fiber sections, respectively, is made possible by comparing with the images obtained when the λ plate is inserted in the POM.

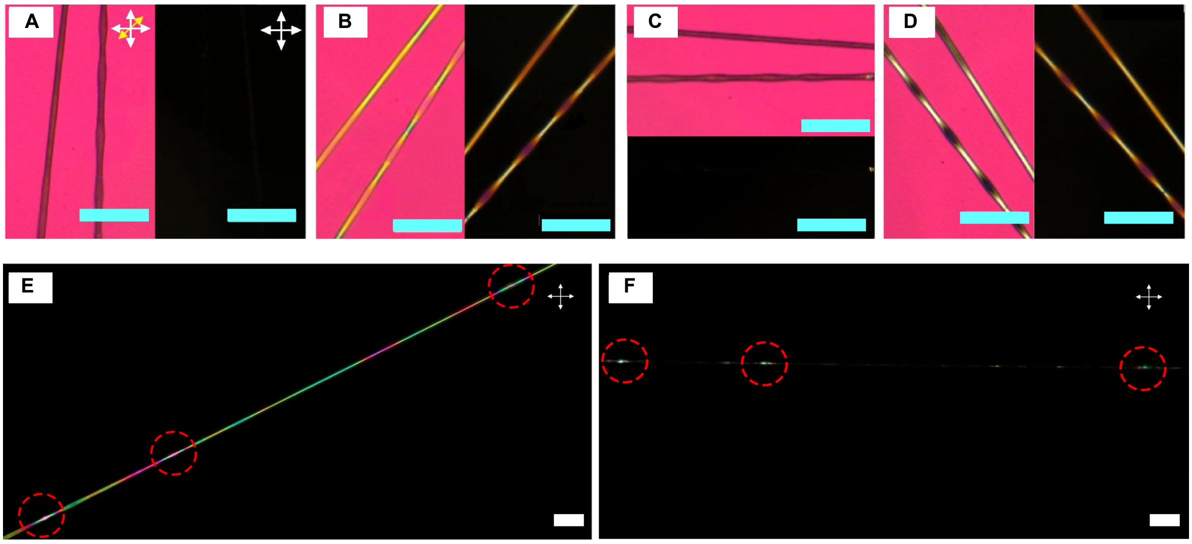

Figure S3: (**A–D**) Close-up POM images of 5CB-core PVP fibers viewed between crossed polarizers with and without a first-order λ plate inserted (pink and black background, respectively). The fibers are oriented 0° (A), 45° (B), 90°(C), and 135°(D) with respect to the polarizer (vertical in the image). (**E–F**) A longer section of a fiber with E7 core is shown between crossed polarizers at 45° (E) and 90° (F) to the polarizer. Scale bars: 20 μm (cyan), 25 μm (white). The optic axis direction of the λ plate is indicated with a yellow double arrow in A. All images are obtained at room temperature (about 25°C).

When the plate is inserted with its optic axis along the fiber axis (left half of B), the yellow segments remain yellow but gain a subtle greenish touch, while the orange beads turn pinkish orange. These shifts are expected for an additional retardation of $r = 551$ nm provided by the λ-plate: comparing with the Michel-Lévy chart we find the same colors at $r \approx 1350$ nm and $r \approx 1020$ nm, respectively. When the fiber is instead oriented perpendicular to the optic axis of the λ–plate, as in the left half of D, the originally yellow straight regions appear white and the originally orange beads are now almost perfectly dark. Inserting the λ-plate with its optic axis perpendicular to the LC director means that we subtract $r = 551$ nm from the original retardance, taking us to $r \approx 250$ nm and $r \approx -80$ nm between and in the beads, respectively. The Michel-Lévy chart is symmetric about $r = 0$, i.e., the color at $r = -80$ nm is the same as at $r = 80$ nm, thus dark grey. At $r = 250$ nm we find an almost colorless white, hence the comparison with the Michel-Lévy chart under the assumption that the director is along the fiber axis perfectly reproduces the colors seen in the fibers. We can thus conclude that the director aligns predominantly along the fiber axis throughout the fiber, and that the beads in these fibers are indeed deprived of LC compared to the unbeaded sections, although the fiber is thicker externally at the beads.

Panels E and F show an E7-filled fiber from the series that was used in the gas sensing experiment, at 45° and 90° angle with respect to the polarizer. This fiber shows a slightly different behavior in the regions with beads. While the fibers in A–D become uniformly dark when they are aligned along or perpendicular to the polarizer, the fiber in E–F has several small regions which remain bright also in F, when the fiber is perpendicular to the polarizer (thus along the analyzer). No matter how the fiber is oriented, these regions (highlighted by red circles) never go entirely dark. This demonstrates the existence of a small twist of the director field in these regions, ensuring that there is always at least part of the core in which the optic axis is neither parallel nor perpendicular to the polarizer.

In Fig. 2 in the main paper, the same type of fiber is shown both between crossed polarizers and without polarizers, and the comparison reveals that the twisted regions are found near beads in the fiber. In contrast to the beads in Figure S3A–D, the core is thicker in these beads, as can be seen by following the sequence of color between crossed polarizers going from the unbeaded to the beaded part and comparing with the Michel-Lévy chart. As a tentative explanation of the difference, we propose that in this case, the Rayleigh instability started acting primarily on the core fluid, leading to accumulation of LC in the beads. Since we need to measure the birefringence in the fiber as a function of toluene exposure, we must ensure that the director field is uniform, not twisted, where we measure. For this reason we avoid the beaded regions during all quantitative gas sensing experiments.

Note S2 Rare cases of toluene exposure-induced irreversible change to the optical properties of LC-core fibers

This note relates to the statement in the second Results section of the main paper, that the vast majority of fibers have intact LC filling and recover their original optical characteristics after VOC exposure and subsequent purging with inert gas, e.g., nitrogen. During our gas sensing studies, three fibers with 5CB core stood out from all others in showing an irreversible change in LC texture after the first toluene exposure. One of them is shown in Figure S4, before and after the first toluene exposure, up to c_{tol} concentrations sufficient to clear the LC. As is easy to see, the birefringence came back after the experiment, but the texture has changed completely, showing a speckled grey-white pattern rather than the smooth colorful texture seen prior to exposure. Since this behavior was very rare, the three fibers where it was observed were clearly malformed, and we speculate that the sheath may have ruptured, allowing the LC filling to partially leave the core during the phase transition. We did not investigate this behavior further, but it can be noted in the left panel of Figure S4 that the fiber has unusually strong color variations, both in magnitude (all colors from violet to red can be seen) and in frequency (maximum length of constant color is on the order of only 10 μm). It may be that the LC filling was too great in this fiber, giving rise to a relatively strong Rayleigh instability impact within the core and rendering the sheath exceptionally thin, thus providing too weak containment.

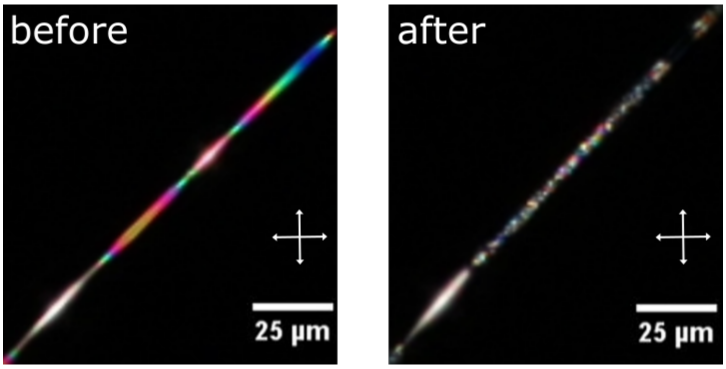

Figure S4: A malformed fiber filled with 5CB before and after exposure to toluene vapor. The concentration of toluene was high enough to induce complete clearing. After removing the VOC by flushing with pure nitrogen, birefringence came back, but the texture was highly irregular. This is an extraordinary phenomenon observed in only three fibers throughout the full study.

We may conclude that a pre-exposure of all fibers to be used as gas sensors is advisable not only in terms of speeding up the response, as discussed at length in the main paper, but also to reveal any malformed fibers. Any fibers that do not recover their pristine optical characteristics, as seen in POM, should be discarded.

Note S3 Response of 5CB-filled fibers to isopropanol vapor

This note relates to the discussion in the main paper of the response of regular LCs to multiple VOCs that have the impact of reducing their order parameter. In a single experiment, a 5CB-filled fiber was brought in contact with saturated vapor of isopropanol (IPA). This was realized with the same set-up as described in the experimental part of the main paper, but using a gas washing bottle filled with liquid IPA instead of toluene. The applied isopropanol flow (u_{IPA}) was 180 mL/min and was kept constant during the whole exposure time, which is tantamount to a constant concentration of isopropanol (c_{IPA}). As Figure S5 shows, the same color change according to the Michel-Lévy chart can be qualitatively

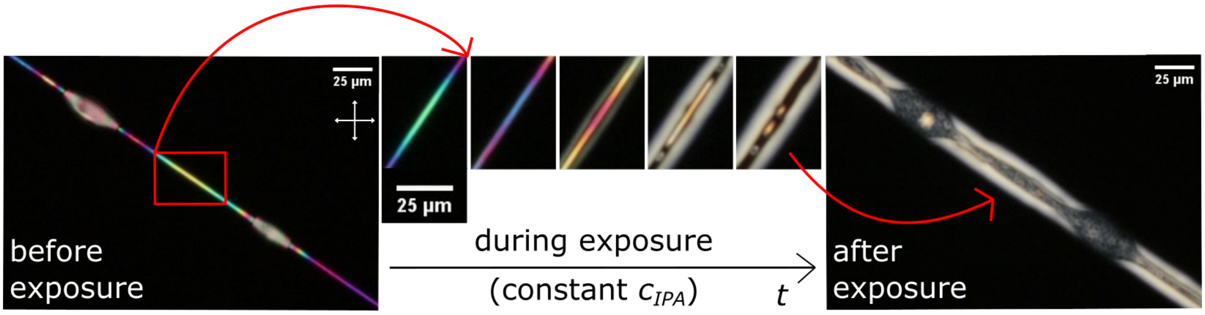

Figure S5: A 5CB-filled fiber between crossed polarizers at $25\ ^{\circ}$C. It was flushed with pure nitrogen gas for 40 s and then exposed to saturated vapor of isopropanol for ≈ 4 min, but without pre-exposure to the analyte. After the fiber core became isotropic, the sample cell was flushed with pure nitrogen again for 50 s. The colorless bright appearance after exposure is due to leaked LC, since PVP is well soluble in isopropanol, leading to an irreversible damage of the polymer sheath. Photos are extracted from a video of the entire experiment and are therefore only snapshots of an actually smooth color transition. The white scale bare of 25 μm of the first small picture is valid for the other ones of same size as well.

Cell Reports
Physical Science

CellPress

observed during the exposure as for the contact with toluene vapor. However, as time progresses a pool of leaking LC grows along the fiber, appearing bright between crossed polarizers. Since PVP is well soluble in IPA, the polymer sheath is irreversibly destroyed and therefore the fiber does not go back to its former appearance during flushing with nitrogen at the end of the experiment.

Note S4 Response of 5CB-filled fibers to cyclohexane vapor

This note relates to the discussion in the main paper of the response of regular LCs to multiple VOCs that have the impact of reducing their order parameter. Different to isopropanol, cyclohexane does not dissolve the PVP sheath and therefore a more extensive, single experiment was carried out. Again, the former gas sensing set-up was used with liquid cyclohexane within the gas washing bottle and the experiment was carried out according to the procedure described in the gas sensing experimental part. Likewise to the toluene experiments, a 5CB-filled fiber was brought into contact with increasing concentrations of cyclohexane (c_{ch}), realized by different flows of nitrogen and cyclohexane (u_{ch}) under constant overall flow of 180 mL/min. Unfortunately the sensitivity of the PID to cyclohexane vapor is unknown and therefore no absolute values for the applied concentrations can be given. However, a calibration of the u_{ch}/u_{N_2} ratio with respect to the PID signal implies that a linear relationship with positive slope (increased u_{ch}/u_{N_2} increases the PID signal) is given for the full working range. A quantitative examination of the response of LC-filled fibers to cyclohexane vapor of known concentrations will be a topic for future studies, once a suitable PID calibration is available.

As Figure S6 shows, the analyte induces a color change that is qualitatively similar to the other two VOCs tested, ending with a transition from the nematic to the isotropic phase. Like for toluene exposure, complete reversibility is given when the analyte is removed by flushing with nitrogen. Unfortunately, since the actual concentrations are unknown, no statement can be made concerning a higher or lower sensitivity of the fibers to cyclohexane in comparison to toluene gas. It should be pointed out that the overall sensitivity depends both on the response of the LC and on the permability of the polymer sheath for each particular choice of VOC.

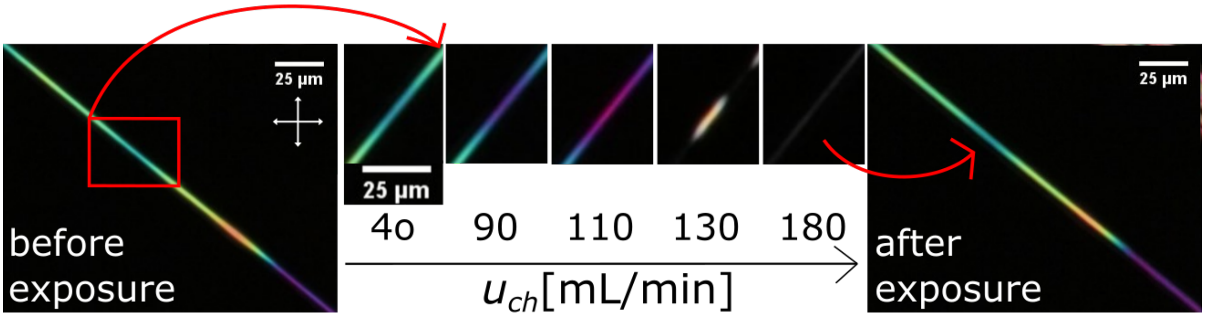

Figure S6: A 5CB-filled fiber between crossed polarizers at $25\ ^\circ$C. Without pre-exposure, it was exposed to different concentrations of cyclohexane vapor realized by increasing u_{ch} and equally decreasing u_{N_2} at a constant overall flow of 180 mL/min. Each flow ratio was held for 1 min, before the next higher ratio was applied (without flushing with pure nitrogen in between). Photos are extracted from a video of the entire experiment and are therefore only snapshots of an actually smooth color transition. The white 25 µm scale bar in the first small picture is valid for the other small pictures as well.

Note S5 Experiment on repeatability with higher number of exposures

This note relates to the assessment of reproducible VOC exposure response, at the end of the Results section of the main paper. To examine if a higher number of toluene vapor exposures still results in the same fiber response, we carried out a single experiment on a 5CB-filled fiber, exposed 10 consecutive times to vapor with $c_{tol} \approx 1.10 \cdot 10^3$ ppm, with flushing steps of pure nitrogen in between. As shown in Table S1, the birefringence of a distinct segment of the fiber decreases during toluene exposure in comparable extent for each repetition, and it fully recovers under nitrogen purging. This can be observed through the POM as a repeatable colour change from green (unexposed) to red (exposed). Response times of these runs lay within the range of 12 s to 21 s. In line with our findings of response time reduction by pre-exposure to the VOC of interest, the fiber was pre-exposed to the analyte; this pre-exposure is not included in Table S1.

Table S1: A single 5CB-filled fiber was exposed 10 times to toluene vapor with $c_{tol} \approx 1.10 \cdot 10^3$ ppm, each time holding for 1 min followed by 3 min of flushing with pure nitrogen gas. An initial pre-exposure step is not included in the number of runs listed. All given birefringence values display an absolute error of ± 0.01.

Exposure	$\Delta n(N_2)$	Δn (tol)	t_r [s]
1	0.22	0.18	18 ± 3
2	0.22	0.18	18 ± 3
3	0.22	0.18	17 ± 3
4	0.22	0.18	18 ± 3
5	0.22	0.18	14 ± 3
6	0.22	0.18	13 ± 2
7	0.22	0.17	12 ± 2
8	0.22	0.17	13 ± 2
9	0.22	0.17	12 ± 2
10	0.22	0.17	21 ± 3

Note S6 Description of video file

This note complements the brief description of the Supplemental Video S1 contained in the main paper. The video shows the response of an E7-filled fiber to increasing concentrations of toluene vapor and serves as an example for visualization of the smooth continuous transition in color as well as the reversibility of the process. The pictures of the E7-filled fiber in 2 and 3 show the exact same fiber, extracted from the full length original video. "Video S1" is an edited, sped up (5 times) and compressed version, starting at $c_{tol} = 1.92 \cdot 10^3$ ppm, which triggers the first visible response. The dark part showing the LC in its isotropic phase ($c_{tol} = 3.8 \cdot 10^3$ ppm) was cut to a sixth of its original duration. Table Table S2 helps to orient in the video, giving the times as they appear in the video at which a certain new concentration is introduced.

Cell Reports
Physical Science

CellPress

Table S2: Times in "Video S1" at which certain concentrations are introduced. Concentration values are given with accuracies of ~ 5 %.

t [min:s]	c_{tol} [10^3 ppm]
00:00	1.92
00:12	2.19
00:24	2.47
00:35	2.7
00:48	3.0
01:00	3.3
01:12	3.6
01:24	3.8
01:26	0

Cell Reports
Physical Science

CellPress

Supplemental Experimental Procedures

Procedure S1 Analysis procedure of the experiment videos by means of the Michel-Lévy chart

This supplemental procedure gives the details on how the color information from experiment videos was extracted and converted into birefringence values, relating to the second Results section and the Experimental procedures section of the main paper. Figure S7a shows the first and second order interference colors of the Michel-Lévy chart plotted as red, green and blue values (R, G, B) versus retardation. These values were obtained by analyzing the chart given by Zeiss [52] using ImageJ Fiji. The triangles are the actual data points that were connected by lines for better visualization. In order to get a single parameter value that can be directly correlated to the optical retardation, the sets of R, G, B values were converted to Hue values (H), using a free to download[1] add-in for Microsoft Excel, producing the H versus r curve in Figure S7b. The conversion works only for the colorful region with retardation greater than 300 nm, because Hue values cannot be used to express gray values. Likewise, r values greater than 750 nm are not shown, as they never occurred during the experiments.

For convenience in fitting a continuous function to the (H, r) data, we added 360 to the H values corresponding to 300 nm$< r <$500 nm. This is valid since hue values lie on a circle, hence H and $H + 360$ describe the exact same point. The advantage of this procedure is that it allows a fitting that contains the whole range of interest. The fitting function determined here is $r = -1.54 \cdot 10^{-9} \cdot H^5 + 1.47 \cdot 10^{-6} \cdot H^4 - 5.34 \cdot 10^{-4} \cdot H^3 + 9.251 \cdot 10^{-2} \cdot H^2 - 8.36 \cdot H + 1059$.

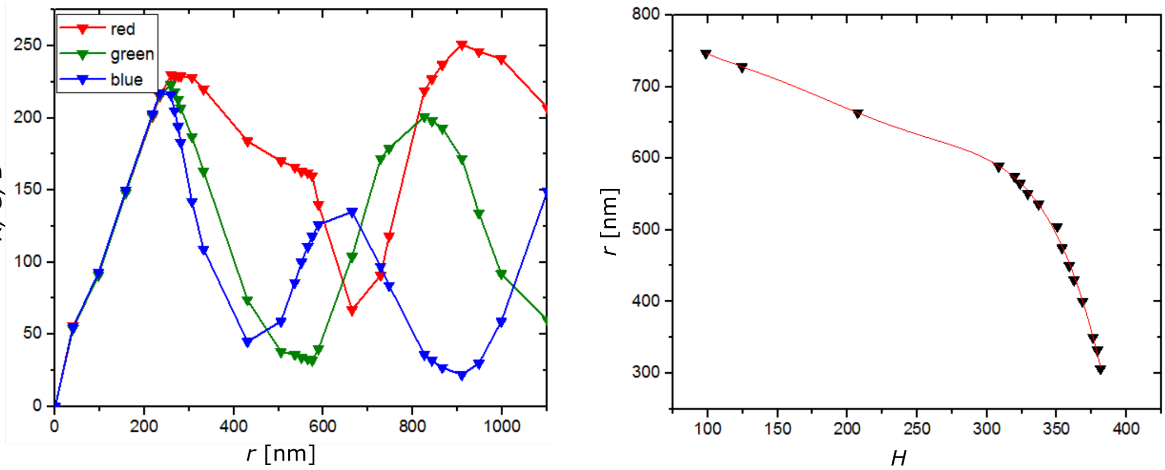

(a) (b)

Figure S7: (a) R, G and B values obtained from the Michel-Lévy chart [52]. The triangles represent the actual data points, the points in between not being recorded. For better visualization, the triangles are connected by straight lines, although the real curves are assumed to procede much smoother than how they are presented here. (b) Michel-Lévy chart in hue values (H) for retardation between 300 and 750 nm, obtained from R, G and B values shown in (a). The function $r = -1.537 \cdot 10^{-9} \cdot H^5 + 1.465 \cdot 10^{-6} \cdot H^4 - 5.337 \cdot 10^{-4} \cdot H^3 + 9.251 \cdot 10^{-2} \cdot H^2 - 8.36 \cdot H + 1059$ was fitted to the data. In order to simplify the fitting, $H = 360$ was added for 300 nm$< r <$500 nm (see text).

For the analysis of the videos recorded of the fiber responses to toluene vapor, segments of fibers that show uniform coloring and no twisting in director were chosen. This means that segments must appear dark with 90° intervals during rotation about the vertical microscope axis. ImageJ Fiji was used to measure the diameter of the fiber at the chosen section for five times and the values were averaged. Therefore, POM pictures with no filters inserted were used. A self-written script in MATLAB

[1] https://exceloffthegrid.com/convert-color-codes/#Convert_from_RGB_to_HSL; accessed 11.02.2021.

(MathWorks) allowed to track the color change during the whole video of an experiment. This means, that a rectangular area, labelled by the pixels of its two opposite corners, was marked and for every twelfth frame of the video ($\sim$ 0.5 s), the red (R), green (G) and blue (B) values of each pixel within this zone were measured and averaged. In a few cases, the position pixels of the rectangular box were shifted during the analysis of a single video, in order to chase the segment of interest, which moved because of minor shaking of the unfixed fibers during the experiment. In order to have only one value, R, G and B were converted to H, following the procedure described above. Again, this was done for data with retardations higher than 300 nm only. The function obtained from Figure S7b was used to transfer H into r and in a final step, equation 1 in the main paper was used to calculate Δn of the fibers under toluene exposure. This was plotted both continuously and as a single value for each concentration step. For the latter case, the last 15 s of the exposure time (1 min) were averaged, since this yields a value closer to that of the equilibrium state.

The response time (t_r) of a fiber to toluene vapor is defined as the time needed after a step change in c_{tol} for the optical response to go from 10 % to 90 % of the saturated equilibrium value. In order to calculate it for the individual concentration steps, the raw data were smoothed over 10 points and the averaged value of the last 15 s of the exposure were set to be 100 %, and the saturation value for the previous c_{tol} exposure was set to 0%. However, this was done for pre-exposed fibers exclusively. Since each concentration was held for only one minute and data showed that this is not long enough to reach 90 % of the response during the first exposure, (t_r) was not calculated in those cases but stated to be > 60 s.

Data were analysed using Microsoft Excel (Microsoft), OriginPro (OriginLab) and ProFit (QuantumSoft), the latter two used for producing the graphs shown in the paper.

Procedure S2 Coaxial spinneret geometries

This description complements the brief description of the electrospinning set-up in the Experimental procedures of the main paper. The spinneret for coaxial electrospinning is built up of a polypropylene Reducing T-coupler (Carl Roth; inner diameter: 3.2 mm for longitudinal openings, 1.6 mm for the lateral) with a stainless steel tubing (Unimed, Lausanne, Switzerland; inner diameter: 0.70 mm, outer diameter: 1.10 mm, length: 50 mm) inserted longitudinally into the T-coupler. The exposed end of the steel tubing represents the needle tip and is connected to the high voltage supply. A flexible, 25 cm long silica capillary coated by polyimide (www.bgb-shop.com, Part #: TSP-250350; inner diameter: 250 μm, outer diameter: 360 μm) is inserted through the steel tubing, positioned with its orifice flush with that of the steel tubing. The other end extends from the other longitudinal opening of the T-coupler. All parts are fixed tight with shrinking tubes and polytetrafluoroethylene (PTFE) tape.

The free end of the silica capillary is fitted to a PTFE tube that leads to a Flow Unit (Fluigent; see below). Further PTFE tubing leads to a 2-way bidirectional valve, which connects (again via PTFE tubing) the reservoirs for LC and ethanol, respectively. The total tubing length from LC container to the exit orifice of the spinneret is roughly 36 cm. The lateral opening of the T-coupler is connected to a PTFE tube (length ~ 62 cm) as well, leading to the reservoir of the PVP solution.

All reservoirs are connected to a microfluidics pressure control unit for dynamic flow control.

Procedure S3 Flow calibration

This description complements the brief description of the electrospinning set-up in the Experimental procedures of the main paper. To measure the flow velocity u_{LC} of the LCs during spinning, we used a Fluigent Flow Unit (size M), with a scale factor determined according to the instructions from the manufacturer[2]. The determined scale factor for 5CB and E7 was 0.8 and 0.9, respectively. The calibrated

[2] https://www.fluigent.com/product/microfluidic-components-3/frp-flow-rate-platform/, accessed 01.02.2021]

reference medium of the Flow Unit was isopropanol and for each LC a 3-point calibration was carried out over three days, whilst each pressure (p) level was measured three times. Calculations were based on the densities[3] $\rho_{5CB} = 1.008$ g/mL and $\rho_{E7} = 1.03$ g/mL.

For the PVP solution we avoided using Flow Units for measuring flow velocities, as PVP residues in the Flow Unit can impact its performance negatively. Instead, we resorted to applying the well-known Hagen-Poiseuille equation:

$$u_{PVP-sol} = \frac{p\pi r^4}{8\eta L} \tag{S3}$$

for calculating the flow velocity $u_{PVP-sol}$ of the PVP solution from the applied pressure p (in practice being equal to a pressure difference between the atmospheres on the two sides of the fluid volume). The equation assumes a constant radius r of the channel, a constant viscosity η and a well-defined length L of the channel. While in our system r varies with location, we are not varying any parameters other than the pressure during our experiments (within the flow rate window probed in the experiments, we deem variations in the PVP solution viscosity negligible), hence we simply identify a proportionality constant describing the linear relationship between $u_{PVP-sol}$ and p. To this end, we carry out an external 3-point calibration, measuring flow rates at each p level three times over three days. A certain pressure was applied for one minute and a glass vial was placed over the needle tip to collect the pumped-out PVP solution. It was weighed using a precision balance and $u_{PVP-sol}$ was calculated. Figure S8 shows the calibration curve. While a good linear fit was obtained, a correlation coefficient of R^2= 1 resulting from weighted linear regression, it required having a non-zero pressure-independent contribution. This may at first may seem unreasonable since it would lead to non-zero flow at zero applied pressure. We believe the reason is imperfect sealing in the system for pressurizing the vial, leading to a non-negligible pressure leakage. We thus write the function obtained from fitting as $u_{PVP-sol} = 0.03074 \cdot (p - 86.6)$ which can be considered valid in the range of applied pressures investigated, thus $250 < p < 800$ mbar.

Data were analysed using Microsoft Excel (Microsoft) and further analysis as well as graphing was done with OriginPro (OriginLab).

[3]The source of the density of 5CB was the Sigma-Aldrich safety data sheet for this chemical (found by searching for product #: 328510, product name: 4'-Pentyl-4-biphenylcarbonitrile, CAS: 40817-08-1; accessed 04.02.2021). For E7, the density was taken from Kim, Y.K.; Senyuk, B.; Lavrentovich, O.D. Molecular reorientation of a nematic liquid crystal by thermal expansion. *Nat. Commun.* (2012). https://www.doi.org/10.1038/ncomms2073.

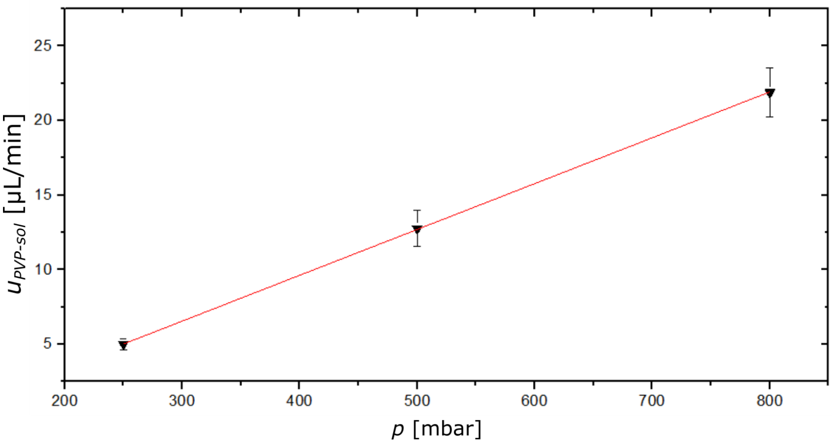

Figure S8: Calibration curve for flow of PVP solution against applied pressure used during electrospinning. Linear regression yields the relationship $u_{PVP-sol} = 0.03074 \cdot (p - 86.6)$.The calibration was repeated on three individual days.

Procedure S4 Holding cell geometries

This description complements the brief description of the gas sensing set-up in the Experimental procedures of the main paper. The custom-built aluminum cell serving as holder for fiber samples during the gas sensing experiments can be seen in Figure S9. It is proven to be gas-tight and designed in a way, that a commercially available glass slide, which is used as substrate for the fibers, fits in perfectly. The inner volume is about 15 mL, including the volume of the in- and outlet. The inset carved in the bottom of the cell is ~ 2.5 mm in height. Removable glass cover slips seal the window on top and bottom such that they can easily be replaced. The photo in Figure S9 was taken before two heating resistors were fitted into the top piece and an electrical temperature sensor on one side of the bottom piece. These additions allowed temperature control of the cell during experiments.

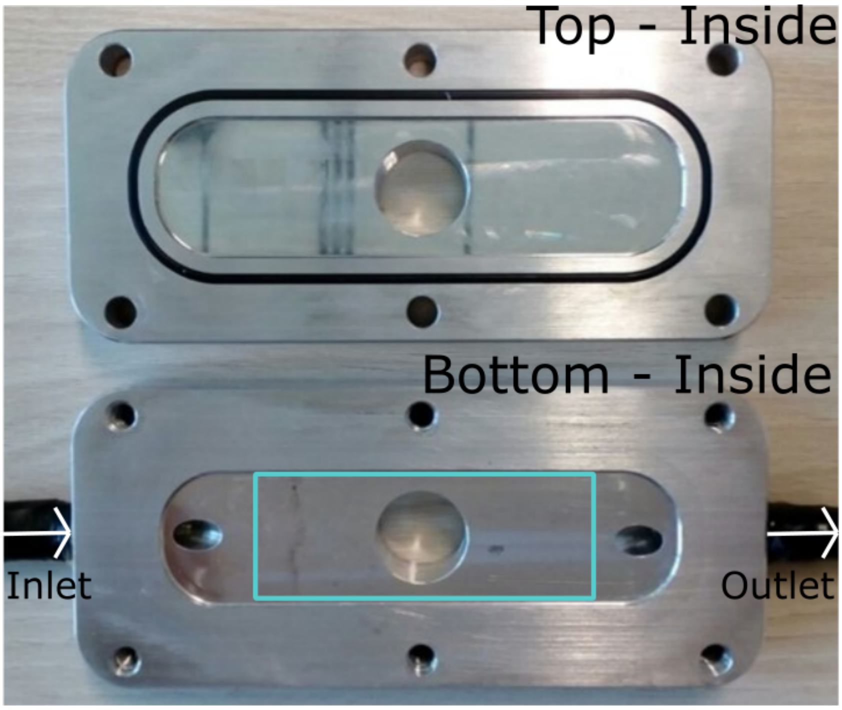

Figure S9: Holding cell for fiber samples during gas sensing experiments. The inside is designed to fit a commercially available glass slide (light blue rectangle) which serves as substrate for fibers.

Procedure S5 Calibration of the photo ionization detector (PID)

This description complements the brief description of the gas sensing set-up in the Experimental procedures of the main paper. As mentioned in the main paper, different concentrations of toluene vapor were realized by dilution of a toluene gas stream with a stream of pure nitrogen, and both flows were adjusted using mass flow controllers (MFC). In this subsection as well as in the following one, all steps needed to calibrate the whole set-up and therefore to allow a translation between toluene-nitrogen-flow ratios and toluene concentration are described in detail and accuracies are discussed. The tubings leading nitrogen only are out of polyurethane (Festo, inner diameter: 5 mm, outer diameter: 6 mm), the remaining ones are of polyamide (Landefeld, inner diameter: 5 mm, outer diameter: 6 mm).

To measure concentrations of toluene, a photo ionization detector (PID) was used. In the first step, the PID response to known concentrations of toluene was checked. Therefore, the testing gas bottle

with 216 ppm of toluene was connected to an MFC directly, and the same was done with a gas bottle of pure nitrogen. The streams were combined behind the MFCs and led through the PID. The baseline signal was determined by measuring the response from pure nitrogen flow (u_{N_2}) for three times a day over six days in total. The flow ratios (u_{N_2} : u_{tol} in mL/min) used for the calibration were 500:0, 400:100, 250:250, 100:400 and 0:500. Since the PID was encapsulated in a chamber of high volume in relation to the preceding tubing system, the reading needed some time (a few minutes) to stabilize. After reaching this point, the PID signal (V, [mV]) was noted every 15 s for a total of 90 s. This whole procedure was repeated over three days. To convert V into toluene concentration (ppm), the baseline signal was subtracted and the result was multiplied, first, by a correction factor (provided by the supplier[4]) of 0.56 specific for toluene, since the PID is originally calibrated to isobutylene, and then by a sensitivity factor.

For the evaluation of accuracies and error propagation, the reading error of the multimeter[5] (PeakTech 1035, DCV $\pm$ 0.5 % + 2 dgt.), accuracy of the MFCs[6] (MKS, $\pm$ 1 % of set point for 20 to 100 % of full scale, $\pm$ 0.2 % of full scale for 2 to 20 % of full scale) and standard deviations of the measurements were considered where needed. Table S3 shows that the experimental data fit the expected theoretical values very well. Figure S10 gives the resulting calibration curve of PID signal to toluene concentration with the function $c_{tol} = 0.41745 \cdot V - 29.61528$. Here, the concentration of pure nitrogen flow was assumed to equal zero since this is reasonable from a physical point of view. Because of the slightly different standard deviations of each ratio value, a weighted linear regression was done. However, the weighting does not show any measurable effect. The correlation coefficient R^2 = 1 illustrates a highly linear correlated behavior. The standard error resulting from this PID-calibration equation was calculated under assumption of normal distribution.

Table S3: Theoretical values for toluene concentrations and values obtained experimentally using the PID for specific flows of toluene vapor u_{tol} (compensated with pure nitrogen to yield an overall flow of 500 mL/min). Experimental data match the theoretical within the margin of deviation.

u_{tol} [mL/min.]	Theoretical c_{tol} [ppm]	Experimental c_{tol} [ppm]
0	0.00	4 ± 2.8
100	43.2	47 ± 4.1
250	108	110 ± 8.5
400	173	172 ± 7.3
500	216	$2.1 \cdot 10^2 \pm 10.4$

Procedure S6 Calibration of the gas sensing set-up

This description complements the brief description of the gas sensing set-up in the Experimental procedures of the main paper. Because it takes a few minutes until the PID shows stable values, due to the large volume of the PID chamber, the toluene concentration cannot be measured directly during the experiments and an external calibration of the set-up is needed. The construction is shown in Fig. 8 of the main paper. For the calibration, the PID was placed at the outlet of the set-up and the switching valves were in a position allowing the flow to pass the sample chamber (temperature-regulated to 25 °C). As calibration points, we chose the flow ratios (u_{N_2} : u_{tol} in mL/min) 180:0, 160:20, 150:30, 140:40, 130:50 and 125:55. The total flow of 180 mL/min was chosen because this is the total flow during the experiments as well. After the signal became stable, values were taken every minute for three minutes in total. The whole procedure was repeated during three days.

[4] http://www.alphasense.com/WEB1213/wp-content/uploads/2017/05/AAN-305-06.pdf; accessed 01.02.2021.
[5] https://www.peaktech.de/produktdetails/kategorie/digital---handmultimeter/produkt/p-1035.html; accessed 01.02.2021.
[6] https://www.mksinst.com/f/ge50a-mass-flow-controller; accessed 01.02.2021.

Cell Reports
Physical Science

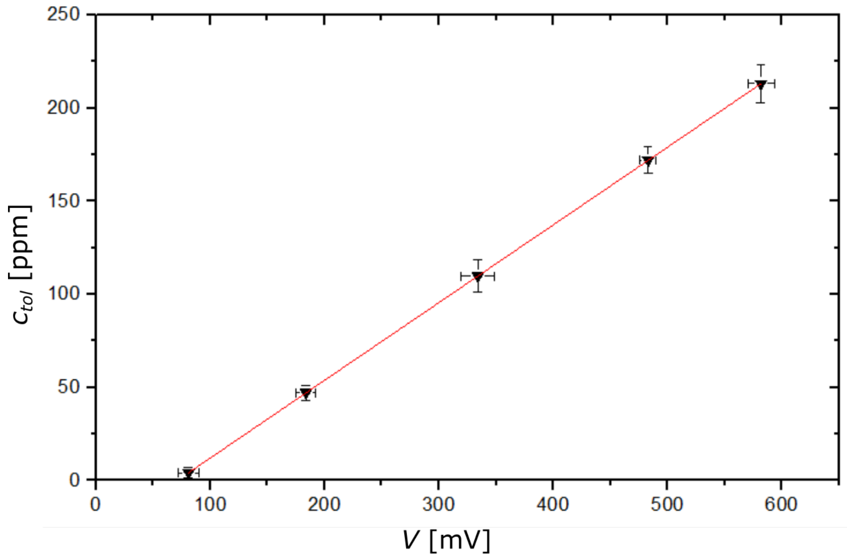

Figure S10: Calibration curve of toluene concentration (c_{tol}) to PID signal (V) with $c_{tol} = 0.41745 \cdot V - 29.61528$ and R^2 = 1. The calibration was repeated on three individual days.

Accuracies were determined considering errors resulting from the linear regression of the PID-calibration, standard deviations of the measurements and reading errors of the equipment (see previous section). Weighted linear regression gave the function $c_{tol} = 27.40048 \cdot u_{tol} + 0.49901$ and a correlation coefficient of R^2 = 0.9962 still expresses a significant linear correlation between toluene flow u_{tol} and concentration. Again, the first calibration point was set to zero for physical reasons.

Since the PID was operated at 3.6 V, the maximum reading is limited to this value, which corresponds to slightly more than 55 mL/min of toluene, at an overall flow of 180 mL/min (compensated with pure nitrogen). Unfortunately, our experiments required us to increase u_{tol} up to a practical maximum value of $u_{tol} = 140$ mL/min, thus exceeding the practical read-out scale of our PID. To verify that linearity still holds for $u_{tol} > 55$ mL/min (at a constant overall flow of 180 mL/min), three flow combinations with constant ratio were flown, differing only in overall flow. The combinations were 357:100, 429:120 and 500:140 and experiments were repeated on three different days. The obtained concentrations were compared to the calculated concentration resulting from the combination 141:39, that shows the same ratio but lies within the calibration range and has an overall flow of 180 mL/min, which allows to apply the received calibration function. The results are presented in table Table S4. Since all three data points match the theoretical value within the given margins of deviation, linearity is proven for flows up to 140 mL/min and concentrations corresponding to toluene flows higher than 55 mL/min can be extrapolated by the given function.

Table S4: Toluene concentrations measured for three flow combinations of same ratio but different overall flows and one combination of the same ratio, which is calculated using the obtained calibration function, since it is within the calibration range at an overall flow of 180 mL/min. The measured values match the calculated one within the margin of deviation and therefore linearity is proven for u_{tol} up to 140 mL/min.

Type	$u_{N_2} : u_{tol}$ [each flow in mL/min.]	c_{tol} [10^2 ppm]
Experimental	357:100	10.9 ± 0.83
Experimental	429:120	11.0 ± 0.75
Experimental	500:140	10.7 ± 0.23
Calculated	141:39	10.8 ± 0.38

Cell Reports
Physical Science

CellPress

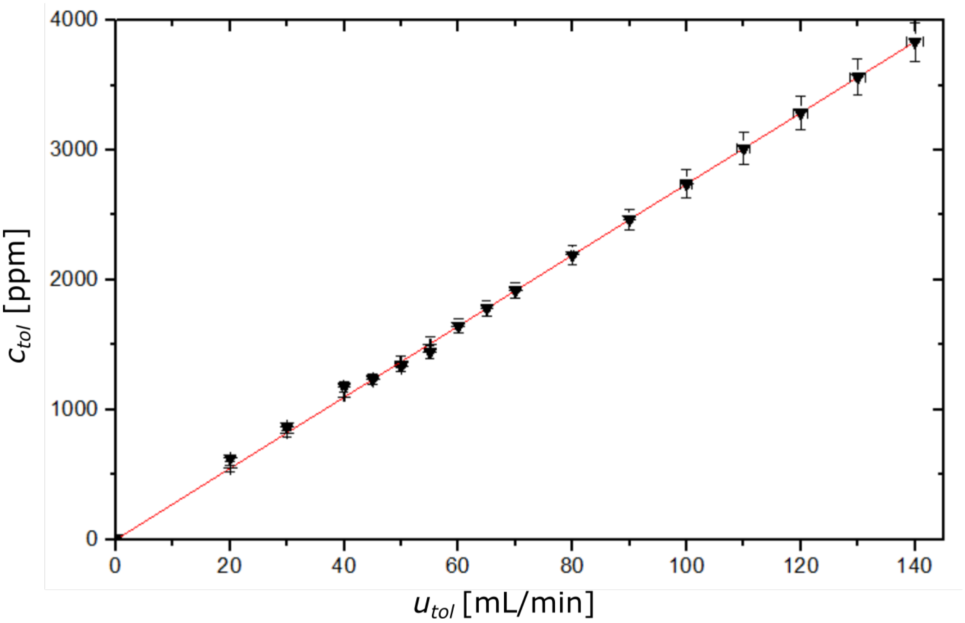

Figure S11: Calibration curve used to convert u_{tol} (at a constant overall flow of 180 mL/min, compensated with respective flows of nitrogen) to toluene concentration in ppm, following the function $c_{tol} = 27.40048 \cdot flow_{tol} + 0.49901$. Data points at $u_{tol} > 55$ mL/min are extrapolated, since linearity is proven for u_{tol} up to 140 mL/min (see table Table S4).The calibration was repeated on three individual days.

Figure S11 shows the final calibration curve used to convert u_{tol} (at a constant overall gas flow of 180 mL/min, compensated with respective flows of nitrogen) to toluene concentration in ppm. Data points at $u_{tol} > 55$ mL/min are extrapolated. Accuracies are determined under consideration of the errors resulting from linear regression and a factor for t-distribution (68 % confidence interval).

In this work we give values of c_{tol} in the commonly used unit ppm (parts per million). To avoid ambiguity concerning its interpretation, we point out that 1 ppm toluene means 1 mol toluene in 10^6 moles of all gases in the atmosphere. With our gas mixtures being solely toluene and nitrogen, 1 ppm would thus be defined as 1 mol of toluene and 999,999 moles of nitrogen. The ppm value can be converted[7] to mg/m^3 (referring to the mass of toluene in the volume of the gas mixture) by multiplication by 3.83 mg/m^3/ppm.

[7] https://gestis.dguv.de/data?name=010070; accessed 01.02.2021.
